# What do We Mean by Environmental Heterogeneity? Toward A Mechanistically Explicit and Scale‐Dependent Framework

**DOI:** 10.1002/ece3.74155

**Published:** 2026-08-14

**Authors:** Iván Hérnandez‐Chávez, Giovani Hernández‐Canchola, Celia López‐González, Livia León‐Paniagua

**Affiliations:** ^1^ Universidad Nacional Autónoma de México, Facultad de Ciencias, Departamento de Biología Evolutiva, Museo de Zoología “Alfonso L. Herrera”, Laboratorio de Mastozoología Evolutiva y Colecciones Científicas Mexico City Mexico; ^2^ Universidad Nacional Autónoma de México, Posgrado en Ciencias Biológicas Mexico City Mexico; ^3^ Instituto Politécnico Nacional, Centro Interdisciplinario de Investigación para el Desarrollo Integral Regional, Unidad Durango Durango City Durango Mexico

**Keywords:** biodiversity patterns, conceptual framework, ecological mechanisms, environmental heterogeneity, quantification methods, scale dependence

## Abstract

Environmental heterogeneity (EH) is widely recognized as a key driver of biodiversity, yet its conceptual foundations remain insufficiently structured. Here we present a semi‐systematic narrative synthesis of 360 studies retrieved from Web of Science and Scopus, spanning 2000 to 2025, to evaluate how EH has been defined, quantified, and analyzed across taxa, regions, and biological disciplines. Only 10.0% of studies (*n* = 36 of 360) provided an explicit definition of EH, and definitions were predominantly descriptive, lacking specification of scale, structural properties, or underlying mechanisms. Most studies (91.9%; *n* = 331 of 360) reported significant EH effects across taxa, regions, and disciplines. Building on our synthesis, we identify four operational interpretations of EH: environmental variability, habitat diversity, environmental differentiation, and structural heterogeneity, each linked to distinct quantification approaches and biological mechanisms. We propose an operational definition of EH as spatial and/or temporal variation in abiotic and/or biotic environmental conditions, across one or multiple scales, that influences resource distributions, environmental filtering, niche differentiation, demographic dynamics, or adaptive processes. Misalignment among EH definitions, measurement strategies, and hypothesized mechanisms emerges as the primary source of inconsistency in the literature. We provide a Research Roadmap linking EH metrics and spatiotemporal scales to detectable biological mechanisms, advancing EH from a descriptive concept toward a predictive, mechanistically explicit framework in biodiversity science, and formulate five broad mechanistic hypotheses linking environmental heterogeneity to biological responses across scales.

## Graphical abstract

We synthesized 360 studies on environmental heterogeneity (EH) across taxa, regions, and disciplines. Only 10% of studies explicitly defined EH, and definitions were predominantly descriptive. We identify four operational interpretations of EH and propose a Research Roadmap linking EH metrics to five mechanistic hypotheses with testable predictions, advancing EH toward a predictive framework in biodiversity science.

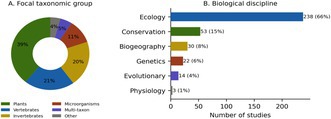

## 1 Introduction

The 21st century has witnessed profound advances in biodiversity research, alongside unprecedented rates of biodiversity loss. We are currently facing the sixth great mass extinction in Earth's history (Dasgupta and Ehrlich 2019), driven primarily by habitat loss (Pardini et al. 2018), invasive species (Parkes and Panetta 2009), and anthropogenic climate change (Magnan et al. 2021). At the same time, biology has become increasingly interdisciplinary, integrating concepts, tools, and perspectives from multiple scientific fields. This integration has enabled researchers to investigate complex ecological and evolutionary systems at finer and broader spatial, temporal, and organizational scales than ever before (Manel et al. 2003; Schoener 2011; Hampton et al. 2013).

Environmental heterogeneity (EH) has emerged as a key concept within this integrative framework. Broadly, EH encompasses variation in landscape characteristics, including spatial complexity, diversity, heterogeneity, or environmental structure (Stein et al. 2014). In the context of global change, EH has become a valuable tool for understanding biodiversity patterns, as variation in local environmental conditions can influence species distributions, community composition, and ecosystem functioning. EH‐based approaches have been used to identify areas of biodiversity loss, characterize potential climatic or ecological refugia, and inform conservation planning by highlighting the importance of heterogeneous habitats within protected areas (Starko et al. 2019; Montañez‐Reyna et al. 2023).

Numerous studies have demonstrated that EH is correlated with a wide range of ecologically relevant variables, including species richness (Stein et al. 2014), diversity (Deák et al. 2021), morphological traits (Nikzat et al. 2021), and genetics (Qian et al. 2024), among others (e.g., Waugh and Weimerskirch 2003; Schipper et al. 2008; Amaral et al. 2022). These findings suggest that EH represents an important dimension of environmental variation associated with biodiversity patterns and evolutionary processes, although the mechanisms linking EH to biological responses remain diverse and often implicit.

Despite its widespread application, EH remains conceptually and methodologically heterogeneous. Its quantification varies substantially across studies due to differences in environmental variables, spatial scales, focal taxa, and analytical approaches (Stein et al. 2014). EH has been measured using both biotic components, such as vegetation structure or land cover, and abiotic components, including climatic, edaphic, and topographic factors (Stein and Kreft 2015). This diversity of approaches has often been accompanied by ambiguous terminology and inconsistent definitions, limiting comparability among studies and constraining broader synthesis across ecological and evolutionary research.

Previous syntheses have documented the diversity of terminology and metrics used to describe environmental heterogeneity, particularly in relation to species richness (Stein et al. 2014; Stein and Kreft 2015). However, these studies did not systematically examine how EH is conceptually defined across biological disciplines, how these definitions cluster conceptually, or how they are theoretically linked to ecological and evolutionary processes. As a result, EH is often invoked as an explanatory variable without clear conceptual alignment among definitions, measurement strategies, and hypotheses.

In this study, we move beyond cataloging metrics to address three unresolved issues: (1) How is EH explicitly defined in contemporary biological research? (2) How is it quantified and analyzed? (3) To what extent are definitions, metrics, and hypothesized biological mechanisms conceptually aligned? (4) Is there a relationship between the complexity of the studies and the detection of an effect of EH? By integrating conceptual classification with mechanistic interpretation, we aim to provide a framework that enhances comparability, strengthens hypothesis formulation, and advances theory development in ecology and evolution.

## 2 Methods

### 2.1 Literature Search

We conducted a semi‐systematic narrative synthesis using a PRISMA‐informed screening workflow (Moher et al. 2009). The designation “semi‐systematic” reflects that, while we applied systematic and reproducible search and screening procedures, we did not conduct formal dual‐reviewer inter‐rater reliability assessment at all stages, nor did we perform meta‐analytic quantification of effect sizes, consistent with the scope of a narrative synthesis aimed at conceptual integration rather than statistical aggregation. The complete search, deduplication, and screening workflow is documented in Supporting Information S2.

We performed a comprehensive keyword search in Web of Science and Scopus, spanning from 2000 to December 2025, using the following Boolean string in Web of Science: TS = (“environmental heterogeneity”) AND TS = (“species richness” OR “biodiversity” OR “species diversity” OR “community composition” OR “genetic diversity” OR “genetic structure” OR “phenotypic plasticity” OR “local adaptation” OR “beta diversity” OR “abundance”). An equivalent string was applied in Scopus (TITLE‐ABS‐KEY field). No language or publication‐type filters were applied at the database level; these were applied during screening. Web of Science and Scopus were selected because of their broad interdisciplinary coverage and their ability to retrieve literature across ecological, evolutionary, and environmental sciences, including journals and regional publications not consistently indexed in other bibliographic databases. We selected published empirical (field or experimental) studies in which the term “environmental heterogeneity” or similar terms found in the title, abstract, or keywords were present. Theoretical or modeling studies without empirical data were excluded.

To manage the large volume of retrieved records efficiently, we applied a two‐stage screening procedure. In the first stage, we developed an automated keyword‐relevance filter using the title and abstract of each record: studies were provisionally excluded if environmental heterogeneity appeared only peripherally, i.e., fewer than three occurrences of the term or its derivatives in the abstract, without co‐occurrence with biological response terms such as *species richness*, *diversity*, *abundance*, *community composition*, *genetic structure*, *phenotypic plasticity*, or *trait variation*. This threshold was selected conservatively to minimize false negatives while reducing the volume of clearly irrelevant records. This automated screen excluded 2570 records, leaving 750 for manual review. In the second stage, all 750 records were independently evaluated by the authors based on their titles and abstracts against the four inclusion criteria stated above; 360 met all four inclusion criteria and were retained for data extraction.

Once we compiled the relevant studies from the literature search, we determined whether the studies provided an explicit operational definition of EH. A definition was classified as *explicit* when the authors stated, in their own words, what they understood by environmental heterogeneity in the context of their study, as opposed to simply citing a prior definition or using the term without elaboration. When a definition was identified, we extracted the verbatim text and classified it into one of four conceptual categories based on the dimension of environmental variation emphasized: (i) *spatial variability*, definitions framing EH as variation or differences in environmental conditions across space; (ii) *spatiotemporal variability*, definitions that additionally incorporated temporal fluctuation or instability; (iii) *biotic‐abiotic scope*, definitions explicitly referencing both physical and biological environmental components; and (iv) *structural complexity*, definitions emphasizing vertical or horizontal habitat structure, spatial configuration, or habitat diversity. Classification was conducted independently by two authors (I.H.‐C. and G.H.‐C.), and discrepancies were resolved by consensus.

We then extracted the following information from each paper: (1) we analyzed the introduction of each publication to identify how the authors defined EH; (2) we categorized focal organisms into five groups based on their taxonomic identity: plants (angiosperms, gymnosperms, mosses, ferns, algae, and other photosynthetic organisms), invertebrates, microorganisms (bacteria, plankton, viruses), vertebrates, and multi‐taxon studies, to identify what were the most analyzed groups; (3) we determined the biological disciplines of the studies and the subject areas that employed EH in research; (4) we identified the quantification methods, grouped into broader operational interpretations based on their statistical properties and the dimensions of environmental variation they capture, and statistical analyses employed in each work; and (5) we evaluated whether the studies detect an effect of EH on the examined variables.

Finally, to assess whether there is a relationship between the complexity of the publications and the detection of an effect of EH, we developed an Analytical Complexity Index (ACI) to characterize the analytical scope of each study, based on three dimensions: (1) number of distinct EH components analyzed; (2) number of distinct quantification methods used; and (3) statistical sophistication (score 1 = ANOVA or unspecified method; score 2 = Regression or Correlation; score 3 = Ordination/RDA or MANOVA). For each dimension, we assigned a score of 1 (single method or component), 2 (two), or 3 (three or more). Scores were added to produce a total ranging from 3 to 9. Studies with totals of 3–4 were classified as “low complexity,” those with 5 as “medium complexity,” and those with 6 or more as “high complexity.”

ACI=C+Q+S

where C, EH components; Q, quantification methods; S, statistical sophistication.

The near‐complete collapse of the quantification methods dimension of the ACI, with 99.7% of studies using a single method, is not merely a limitation of the index, it is, in itself, a substantive finding that deserves interpretation. Despite the diversity of EH components examined (climatic, topographic, aquatic, edaphic, structural) and the diversity of biological responses studied (species richness, genetic structure, phenotypic plasticity, community composition), researchers overwhelmingly rely on a single quantification approach per study. This suggests that methodological choices are driven largely by data availability and analytical convention rather than by explicit consideration of which metric best captures the dimension of EH hypothesized to drive the biological response. The standard deviation of a climatic variable, an index of habitat‐type diversity, and an environmental distance matrix are not interchangeable: each captures a different aspect of environmental variation and is suited to testing a different hypothesis. From a hypothesis‐driven perspective, the appropriate metric should follow from the biological mechanism under investigation rather than from analytical convention alone (Austin 2007; Wiens 1989). The hypothesis should specify both what aspect of the environment varies and why that variation is expected to influence the focal organism. Under this logic, the choice of EH metric becomes part of the inferential framework rather than a purely methodological decision. The homogeneity of quantification approaches in our dataset suggests that this logical sequence, from hypothesis to metric to statistical test, is more the exception than the rule in current EH research. This represents one of the most tractable targets for methodological improvement in the field.

## 3 Results

### 3.1 Patterns in Environmental Heterogeneity Research

#### 3.1.1 Temporal Trends in Environmental Heterogeneity Studies

**FIGURE 1:**
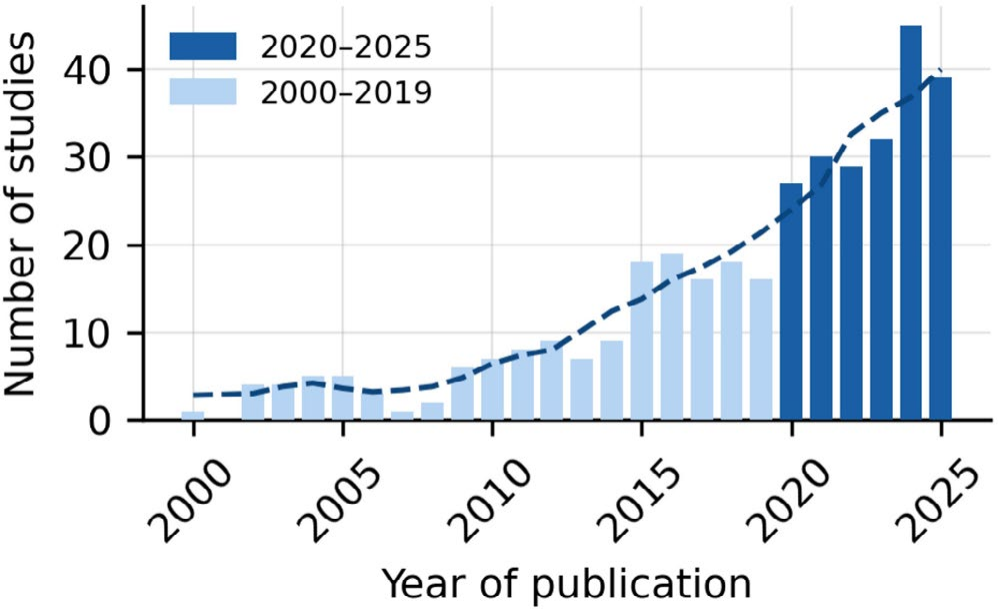
Annual number of EH studies retrieved from Web of Science and Scopus (2000–2025; *n* = 360). Light blue bars = 2000–2019; dark blue bars = 2020–2025, which account for 55.8% of all studies (*n* = 200). Dashed line = LOESS smoothing trend. The most productive years were 2024 (*n* = 45) and 2025 (*n* = 39).

A total of 360 studies meeting our inclusion criteria were retrieved from Web of Science and Scopus, covering 2000–2025. The complete list of reviewed studies is provided in Supporting Information S1. Publication output increased markedly over time (mean: 14.4 studies/year). Growth was particularly pronounced after 2019, with studies from 2020 to 2025 accounting for 55.8% of all records (*n* = 200). The most productive years were 2024 (*n* = 45) and 2025 (*n* = 39; Figure 1).

#### 3.1.2 Taxonomic Focus of EH Research

**FIGURE 2:**
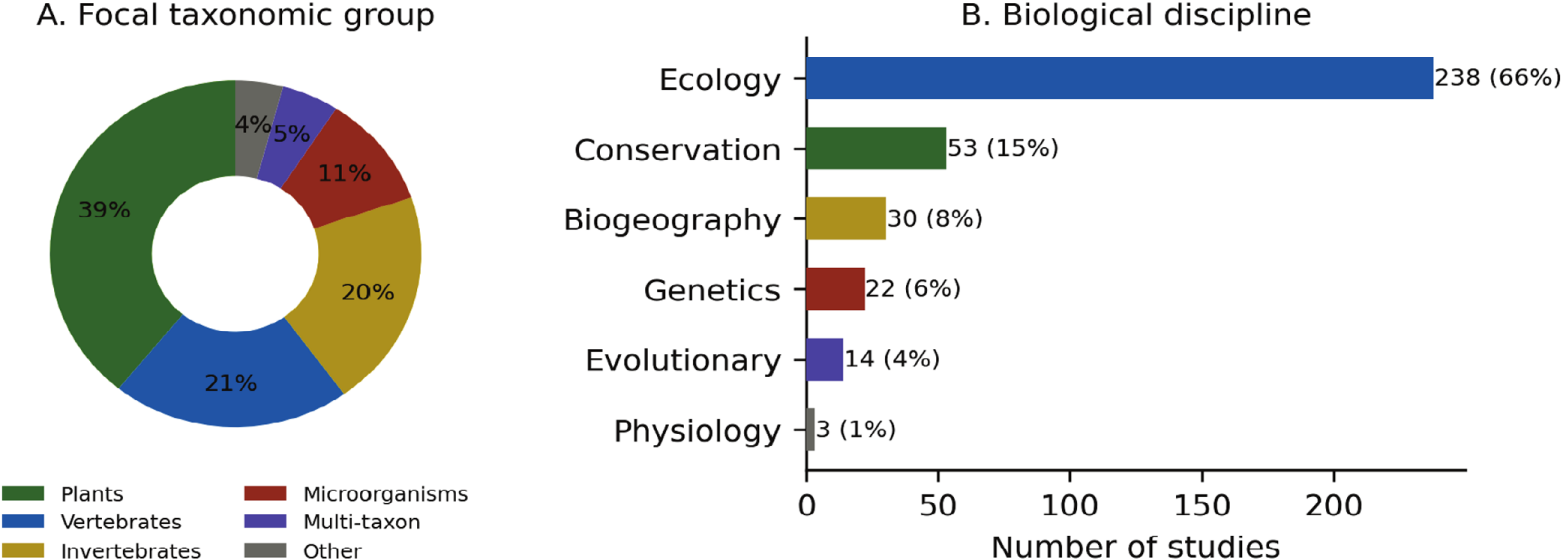
Taxonomic and disciplinary scope of EH studies (*n* = 360). (A) Proportion of studies by focal taxonomic group. (B) Number of studies per biological discipline. Multi‐taxon studies include works examining two or more taxonomic groups simultaneously.

EH research showed a diversified taxonomic scope. Plants constituted the largest group (*n* = 141; 39.2%), followed by vertebrates (*n* = 75; 20.8%), invertebrates (*n* = 73; 20.3%), microorganisms (*n* = 38; 10.6%), multi‐taxon studies (*n* = 18; 5.0%), and other organisms (*n* = 15; 4.2%; Figure 2).

#### 3.1.3 Geographic Distribution of Studies

**FIGURE 3:**
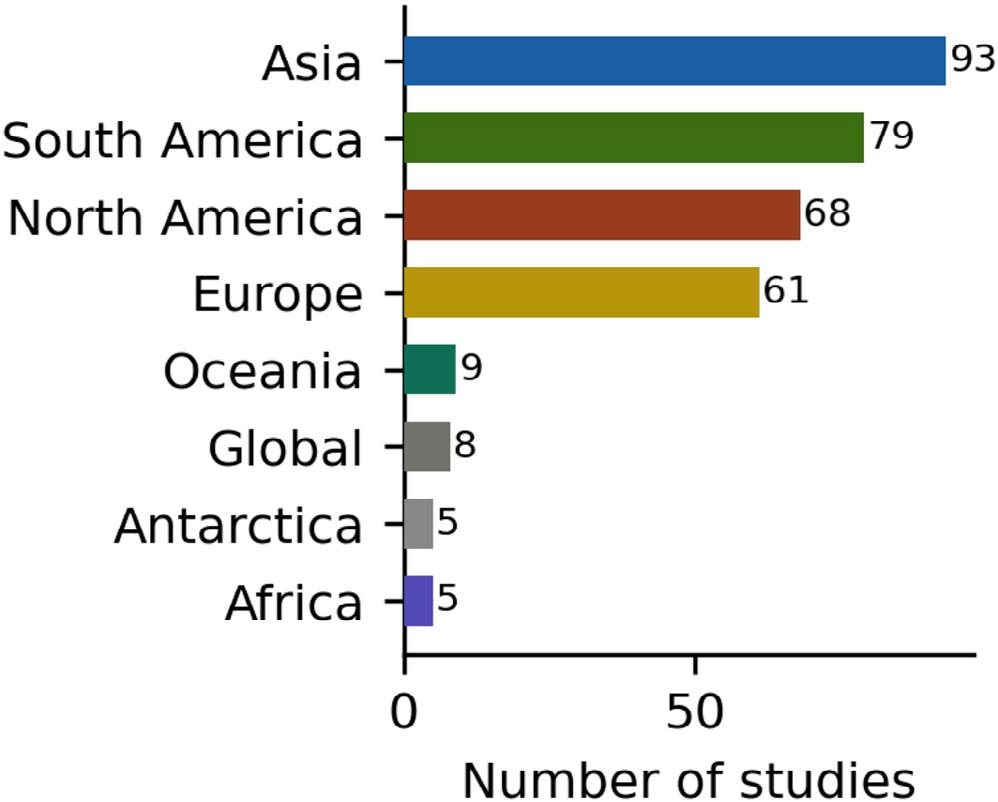
Geographic distribution of EH studies by continent (*n* = 360). One study with missing region information excluded. Note that Web of Science and Scopus proportionally index more literature from Asia, Europe, and North America, so the distribution may partly reflect differences in database indexing coverage rather than an absence of EH research in underrepresented regions.

The geographic distribution of EH studies was highly uneven. Most research was conducted in the Americas, with the United States, Brazil, and Mexico accounting for a substantial proportion of studies. Europe represented a secondary focus, whereas Africa and large regions of Asia were sparsely represented. Studies were predominantly terrestrial, with aquatic and marine systems receiving comparatively limited attention (Figure 3). We note that the geographic distribution of results may partly reflect a database indexing bias, as WoS and Scopus proportionally index more literature from Asia, Europe, and North America.

#### 3.1.4 Conceptual Nature of Explicit Definitions of Environmental Heterogeneity

**FIGURE 4:**
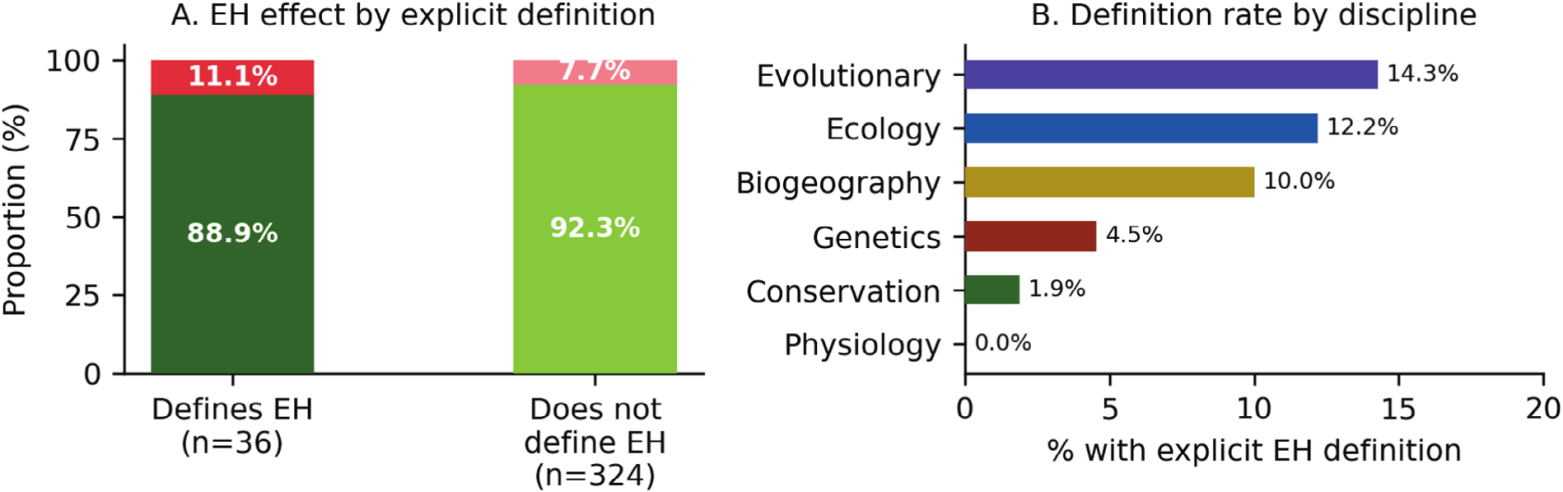
Explicit EH definitions across studies (*n* = 360; 36 with explicit definition, 324 without). (A) Proportion of studies detecting significant EH effects by whether an explicit definition was provided. (B) Percentage of studies providing an explicit EH definition by biological discipline.

Although only 10.0% of studies (*n* = 36 of 360) provided an explicit definition of EH (Supporting Information S2), this subset reveals important patterns in how the concept is operationally understood. The frequency of explicit definitions showed no significant temporal trend over the study period (Spearman ρ = −0.08, *P* = 0.71), indicating that conceptual clarity has not improved alongside the marked growth in empirical output documented in Figure 1. This stasis is noteworthy: despite a fivefold increase in annual publications between 2000 and 2025, the proportion of studies providing an operational definition of EH has remained consistently low across all periods (Figure 4).

Among the 36 studies that provided explicit definitions, we identified four conceptual categories (Table 1). The most frequent category was *spatial variability* (*n* = 18; 50.0%), encompassing definitions that framed EH as variation or differences in environmental conditions across space, without specifying the nature of the varying components or the scale at which variation was assessed. Importantly, most definitions in this category were incomplete in a specific sense: they stated *that* the environment varies spatially without specifying *what* varies, *at what scale*, or *why* that variation should be biologically relevant to the focal organism. As previously emphasized by scale‐dependent ecological theory (Wiens 1989; Levin 1992), environmental heterogeneity cannot be characterized independently of the attribute that varies, the organism perceiving that variation, and the scale at which it is measured. Heterogeneity is always heterogeneity *of something*, perceived *by someone* (or some organism), at *some scale*. Definitions that omit these specifications leave the link between measurement and mechanism implicit, undermining both replicability and comparative synthesis. The second most frequent category was *biotic‐abiotic scope* (*n* = 9; 25.0%), in which definitions explicitly recognized both physical and biological components of the environment. A smaller proportion of studies defined EH in terms of *spatiotemporal variability* (*n* = 5; 13.9%), incorporating temporal fluctuation as an additional dimension. The least frequent category was *structural complexity* (*n* = 4; 11.1%), encompassing definitions that emphasized vertical or horizontal habitat structure, spatial configuration, or habitat diversity.

**TABLE 1:** Representative explicit definitions of EH extracted from the 36 studies (10.0% of *n* = 360) that provided an operational definition.

Conceptual category	*n* (%)	Representative definition	EH component measured	Biological response
Spatial variability	18 (50.0%)	“Spatial heterogeneity in environmental factors, including soil nutrients, moisture and light availability across the landscape” (Lundholm and Larson 2003)	Soil, Vegetation, Topographic	Plant species richness and community composition
Biotic‐abiotic scope	9 (25.0%)	“Spatial and temporal heterogeneity in biotic and abiotic factors, including climate, land use and soil conditions, as well as species interactions” (Albrecht et al. 2021)	Climatic, Land cover, Soil	Species richness, ecosystem functioning, turnover
Spatiotemporal variability	5 (13.9%)	“Spatial and temporal heterogeneity in environmental conditions, including the spatial complexity, diversity and structure of abiotic and biotic factors across scales” (Esparza‐Orozco et al. 2020)	Soil, Topographic, Vegetation	Amphibian community composition
Structural complexity	4 (11.1%)	“Horizontal and vertical variation in habitat structures, including spatial configuration of microhabitats and habitat‐type diversity” (Méndez‐Toribio et al. 2016)	Land cover, Soil, Topographic	Tree community attributes and species diversity

*Note:* Most frequent terms in definitions: variation (86.1% of definitions), spatial (77.8%), conditions (61.1%), habitat (52.8%), resources (38.9%). Terms linking EH to mechanisms (niche, filtering, adaptation, gradient) appeared in < 30% of definitions. The four categories in the table correspond directly to the four operational interpretations in Table 2. Studies in the “Spatial variability” definition category predominantly used Environmental Variability metrics; “Habitat diversity” definitions were associated with Habitat Diversity quantification approaches; “Spatiotemporal variability” definitions with Environmental Variability and Differentiation metrics; and “Structural complexity” definitions with Structural Heterogeneity proxies. This correspondence, though not universal, supports the theoretical alignment proposed in Table 2. Definitions were classified into four conceptual categories by two authors independently (I.H.‐C., G.H.‐C.); discrepancies resolved by consensus. Representative definition selected to illustrate category‐typical language. EH component and biological response reflect information extracted from the same study.

In most cases (*n* = 26 of 36; 72.2%), definitions remained descriptive, focusing on variation or differences in environmental conditions without clarifying scale, structural properties, or mechanistic expectations (e.g., Douda et al. 2011; Bar‐Massada and Wood 2013). Only 10 definitions (27.8%) included explicit reference to a biological mechanism or process through which EH was expected to influence the response variable. Representative examples of each conceptual category, together with the associated EH component, quantification method, and biological response examined, are provided in Table 1.

Textually, the most frequently used terms in explicit definitions were *variation* (appearing in 31 of 36 definitions; 86.1%), *spatial* (*n* = 28; 77.8%), *conditions* (*n* = 22; 61.1%), *habitat* (*n* = 19; 52.8%), and *resources* (*n* = 14; 38.9%). Terms explicitly linking EH to biological mechanisms—such as *niche diversity*, *filtering*, *adaptation*, or *gradient*, appeared in fewer than 30% of definitions. This lexical pattern confirms that EH is predominantly framed in terms of descriptive spatial variation rather than functional ecological process, reinforcing the need for definitional frameworks that bridge description and mechanism.

Importantly, the four conceptual categories identified in Table 1 map onto the four operational interpretations in Table 2: studies whose authors defined EH as spatial variability predominantly used Environmental Variability metrics, such as standard deviation or coefficient of variation of climatic or edaphic variables, while those emphasizing structural complexity used Structural Heterogeneity approaches, suggesting that conceptual framing, though rarely explicit, does shape measurement choices.

**TABLE 2:** Operational interpretations of environmental heterogeneity (EH) identified in the semi‐systematic narrative synthesis (*n* = 360 studies).

Operational interpretation	Core EH dimension	Typical quantification metrics	Common statistical approaches	Hypothesized biological mechanism	Example studies
Environmental Variability	Magnitude of variation in abiotic or biotic conditions across space or time	Standard deviation, coefficient of variation, range, variance of environmental variables (temperature, precipitation, soil nutrients)	Regression, linear mixed‐effects models, Spearman correlation	Environmental filtering → deterministic community assembly; selection pressure → local adaptation and phenotypic plasticity	*Gianoli and González‐Teuber* *2005* *; Lázaro‐Nogal et al.* *2015* *; Esparza‐Orozco et al.* *2020*
Habitat Diversity	Number, richness, or compositional diversity of habitat types or land‐cover classes	Shannon or Simpson diversity index of habitat types, number of habitat categories, SHDI (Fragstats)	Regression, GLM, ANOVA, ordination (RDA/CCA)	Niche availability, resource diversification, species coexistence, habitat complementarity	*Dufour et al.* *2006* *; Alahuhta et al.* *2017* *; Udy et al.* *2021*
Environmental Differentiation	Degree of dissimilarity or turnover in environmental conditions among sites	Euclidean or Mahalanobis distance matrices, Bray–Curtis dissimilarity, Mantel‐based approaches	Mantel test, partial Mantel, distance‐based redundancy analysis (dbRDA)	Environmental sorting, beta‐diversity turnover, genetic isolation by environment (IBE), local adaptation	*Temunović et al.* *2012* *; Fine et al.* *2017* *; Myers et al.* *2019*
Structural Heterogeneity	Vertical or horizontal complexity of habitat architecture and spatial configuration	Vegetation height variance, canopy cover, landscape configuration indices (patch richness, edge density, contagion), LiDAR‐derived metrics	Regression, linear mixed‐effects models, spatial autoregressive models	Structural niche partitioning, resource stratification, microhabitat availability, thermal buffering	*Méndez‐Toribio et al.* *2016* *; Graham et al.* *2019* *; Gastauer et al.* *2020*

*Note:* Each interpretation reflects a distinct dimension of environmental variation, with associated quantification metrics, statistical approaches, and hypothesized biological mechanisms. Interpretations are not mutually exclusive; a single study may incorporate more than one. Example studies are drawn from the reviewed dataset. For testable predictions and recommended statistical approaches associated with each operational interpretation, see Table 3.

#### 3.1.5 Biological Disciplines and Focal Traits

Most EH studies were rooted in ecological research and focused on species‐ or community‐level responses such as species richness, diversity, abundance, and community composition. Fewer studies examined morphological, physiological, or genetic traits, and these were unevenly distributed among taxonomic groups. Genetic and physiological studies were particularly scarce relative to ecological investigations (Figure 2).

#### 3.1.6 Types of Environmental Heterogeneity Analyzed

**FIGURE 5:**
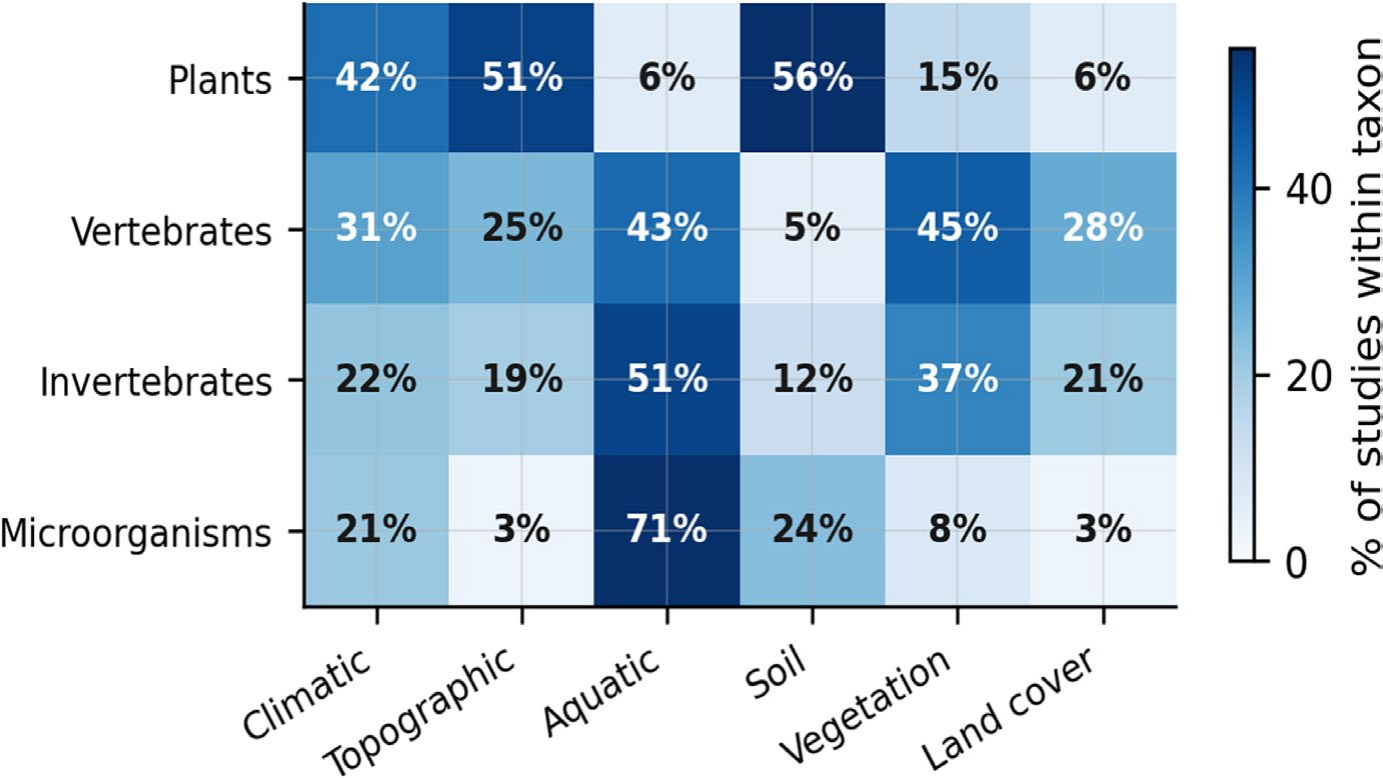
Heatmap of EH component use by focal taxonomic group. Values indicate the percentage of studies within each taxonomic group that included each EH component (multi‐value; rows sum to more than 100%). Only the four most frequently studied taxonomic groups are shown (Plants, Vertebrates, Invertebrates, Microorganisms).

**FIGURE 6:**
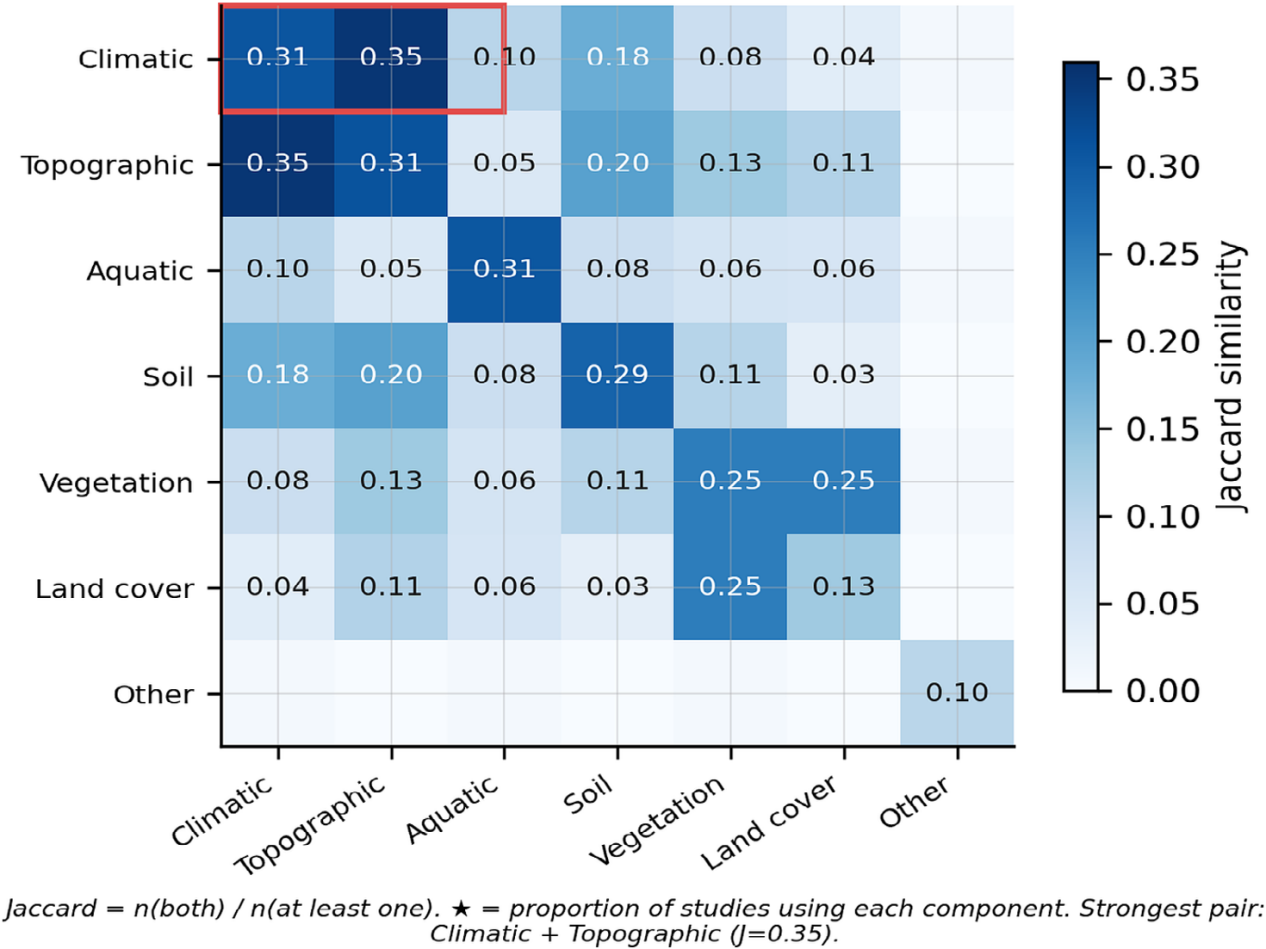
Co‐occurrence matrix of EH components across 360 studies. Values indicate Jaccard similarity (J = *n* studies with both components/*n* studies with at least one component), ranging from 0 (never co‐occur) to 1 (always co‐occur). Diagonal values indicate the proportion of all studies using each component. The strongest pair was Climatic + Topographic (J = 0.35), co‐occurring in 16.1% of studies.

**FIGURE 7:**
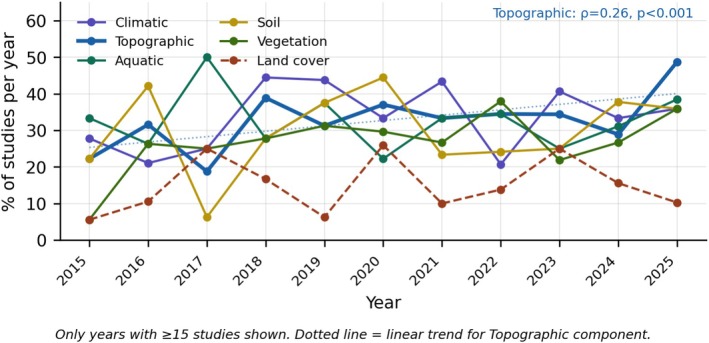
Temporal trends in EH component use (2015–2025; restricted to years with ≥ 15 studies). Values indicate the percentage of studies per year using each EH component. Dotted line = linear trend for the Topographic component, which showed a significant increase over time (Spearman ρ = 0.26, *p* < 0.001). Land cover is shown as a dashed line.

Abiotic components of EH were analyzed more frequently than biotic components. Climatic, topographic, and edaphic heterogeneity were the most commonly used abiotic variables. Biotic EH was primarily represented by land‐cover classes and vegetation structure, whereas other biotic dimensions – such as resource diversity or prey availability‐ were rarely quantified. The relative use of each EH component also varied among focal taxonomic groups (Figure 5). Of the 360 studies reviewed, 187 (51.9%) measured a single EH component, while 173 (48.1%) measured two or more simultaneously. Among multi‐component studies, the most frequent combination was Climatic + Topographic (*n* = 58; 16.1% of all studies; Jaccard similarity = 0.35), reflecting the widespread use of elevational gradients as natural experiments in EH research (Figure 6). The use of the Topographic component increased significantly over the study period, whereas other components remained comparatively stable (Figure 7).

#### 3.1.7 Quantification Methods and Statistical Analyses

**FIGURE 8:**
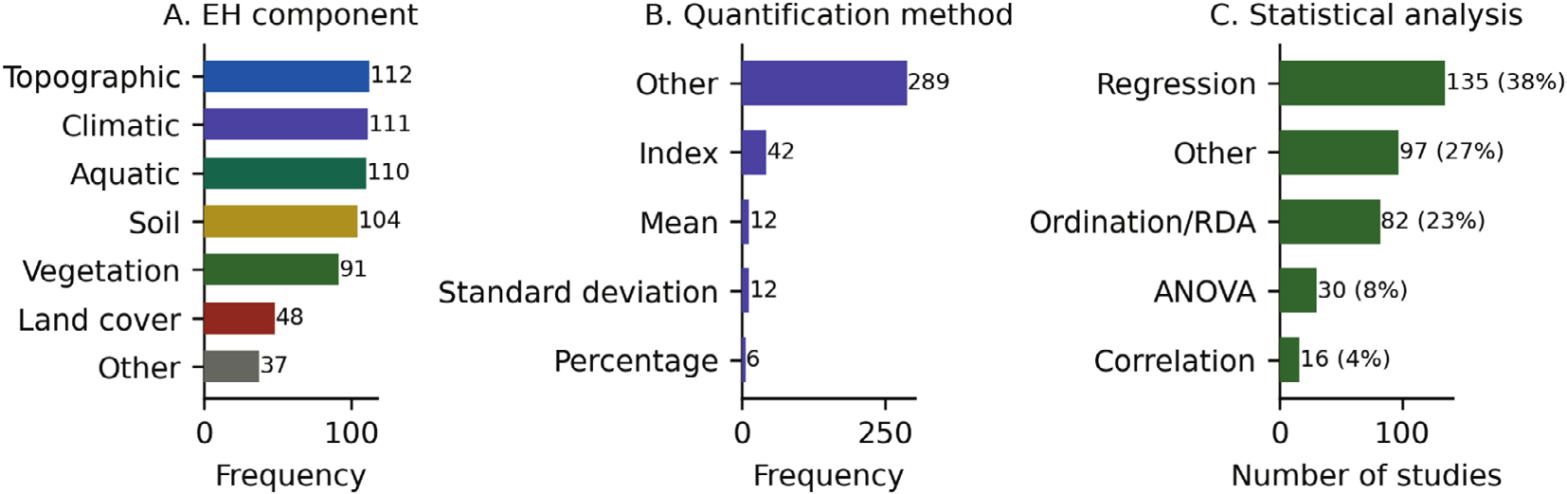
EH operationalization across 360 studies. (A) Frequency of EH components analyzed (Values sum to more than 100% because 48.1% of studies measured two or more components simultaneously). (B) Quantification methods used. (C) Primary statistical analyses applied. All statistical approaches represented are fundamentally measures of association between EH and a biological response variable.

EH was quantified using a wide range of approaches, including continuous environmental variables, categorical classifications, indices of variability, and spatial metrics. These quantification methods were subsequently grouped into broader operational interpretations based on their statistical properties and the dimensions of environmental variation they capture. The most common primary statistical approach was regression (*n* = 135; 37.5%), followed by ordination methods including RDA and CCA (*n* = 82; 22.8%), and unspecified or other approaches (*n* = 97; 26.9%). ANOVA was used in 8.3% of studies (*n* = 30) and correlation‐based methods in 4.4% (*n* = 16). Notably, all approaches represented in the dataset are fundamentally measures of association between EH and a response variable, as Legendre and Legendre (2012) note, ANOVA is a special case of regression for categorical predictors, and ordination methods are multivariate extensions of the same logic. The apparent diversity of statistical approaches therefore reflects variation in response variable type and dimensionality rather than fundamentally different inferential frameworks (Figure 8).

We identified multiple quantification approaches corresponding to distinct operational interpretations of environmental heterogeneity (Table 2). These interpretations reflect distinct ways in which EH is represented in empirical studies, including environmental variability, habitat diversity or composition, environmental differentiation among sites, and structural heterogeneity.

Although the four operational interpretations share a common concern with environmental variation, they differ in fundamental ways that have direct consequences for study design and inference. Environmental Variability and Habitat Diversity are particularly prone to conflation, yet they capture distinct dimensions of EH. Environmental Variability refers to the continuous variation in the *values* of abiotic or biotic variables across space or time, for example, the standard deviation of soil moisture across a plot, or the coefficient of variation in annual temperature across a landscape. Habitat Diversity, by contrast, refers to the richness or diversity of discrete *habitat types* within an area, the number of distinct land‐cover classes, vegetation communities, or microhabitat categories present. A site can be high in Environmental Variability but low in Habitat Diversity (e.g., a continuous gradient in soil salinity with no discrete habitat boundaries), or high in Habitat Diversity but low in Environmental Variability within each habitat type (e.g., a mosaic of climatically similar but structurally distinct vegetation types). The ecological implications also differ: Environmental Variability more directly predicts phenotypic plasticity, physiological tolerance ranges, and selection pressures on individual traits, while Habitat Diversity more directly predicts species richness through niche diversification and beta‐diversity turnover among habitat types. Choosing between them is therefore not a methodological preference but a conceptual commitment that should follow from the hypothesis being tested. It is worth noting that habitat classifications are themselves human constructs—proxies of what we assume constitutes a habitat for the focal organism—and may not capture the dimensions of environmental variation that are ecologically meaningful at the organism's scale of perception (Wiens 1989).

#### 3.1.8 Detection of EH Effects on Biological Responses

The majority of reviewed studies (91.9%; *n* = 331 of 360) reported at least one significant relationship between EH and the biological variables analyzed. We acknowledge that publication bias may partially contribute to the predominance of reported significant effects, as studies detecting null relationships may be less likely to be published. Significant effects were detected across all taxonomic groups, biological disciplines, and EH components considered. The frequency of detected effects varied among study types but showed no clear association with taxonomic focus or geographic region. Detailed results by taxonomic group are provided in Supporting Information S3.

The minority of studies that did not detect a significant EH effect (8.1%; *n* = 29) deserve particular interpretive attention. A non‐significant result in an EH study can arise from at least four distinct sources, which are not always distinguishable post hoc. First, the EH components measured may be irrelevant to the focal taxon; a study measuring soil heterogeneity for a canopy‐dwelling bird, or climatic variability at landscape scale for a soil microorganism, may find no effect simply because the measured attributes do not enter the organism's ecological or evolutionary experience. Second, the scale of measurement may not match the scale of perception: as Wiens (1989) argued, heterogeneity is not an objective property of the landscape but is defined relative to the grain and extent at which an organism responds to environmental variation. A landscape that is measurably heterogeneous at 1 km^2^ resolution may be functionally homogeneous for a species with a home range of 10 m^2^. Third, the response variable chosen may not be the one most sensitive to EH in that system: species richness may not respond to topographic heterogeneity in a taxon whose diversity is primarily limited by dispersal rather than niche availability. Finally, statistical limitations—insufficient sample size, measurement error larger than the biological signal, or the use of a heterogeneity metric that does not capture the ecologically relevant dimension of variation—can obscure real effects. The high overall detection rate in our dataset (91.9%) may itself partly reflect publication bias favoring significant results, making the interpretation of null results all the more important for future synthesis.

#### 3.1.9 Analytical Complexity of EH Studies

**FIGURE 9:**
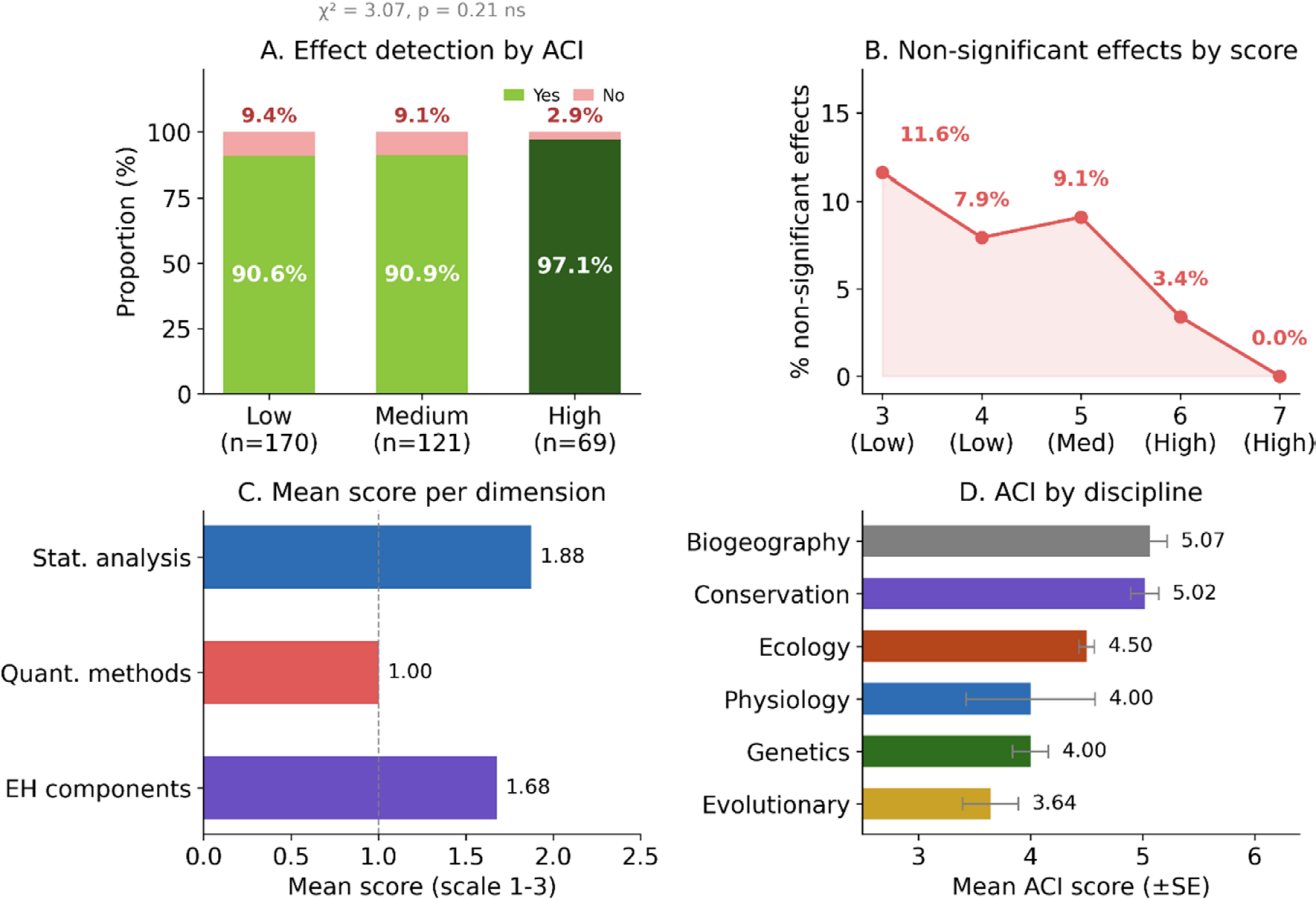
Analytical Complexity Index (ACI) across EH studies (*n* = 360). The ACI combines scores for three dimensions: Number of EH components (C), quantification methods (Q), and statistical sophistication (S), ranging from 3 (minimum) to 9 (maximum). (A) Proportion of studies detecting significant EH effects by complexity level (Low = score 3–4, *n* = 170; Medium = score 5, *n* = 121; High = score 6–7, *n* = 69); effect detection did not differ significantly among levels (χ^2^ = 3.07, *p* = 0.21). (B) Percentage of non‐significant effects by individual ACI score. (C) Mean score per ACI dimension (±SE). (D) Mean ACI score by biological discipline (±SE).

EH studies varied in their analytical complexity according to the Analytical Complexity Index (ACI; see Methods): 47.2% were low complexity (score 3–4; *n* = 170), 33.6% medium (score 5; *n* = 121), and 19.2% high (score 6–7; *n* = 69). The proportion of studies detecting significant EH effects did not differ significantly among complexity levels (Low = 90.6%, Medium = 90.9%, High = 97.1%; χ^2^ = 3.07, *P* = 0.21; Figure 9). We classified regression, GLM, and linear mixed‐effects models as moderate‐complexity statistical approaches (ACI score 2), recognizing that these methods can accommodate multiple predictors and are not strictly univariate. The distinction in our index reflects analytical scope relative to the complexity of the EH measurement rather than the technical properties of the statistical model.

## 4 Discussion

Our synthesis reveals that EH research conflates, often implicitly, three conceptually distinct questions. The first is *descriptive*: characterizing the degree and nature of environmental variation in a given site or region, describing what is heterogeneous, in which attributes, and at what scale. The second is *relational*: testing whether that variation is statistically associated with a biological response, whether more heterogeneous sites tend to support more species, more genetically diverse populations, or organisms with greater phenotypic plasticity. The third is *explanatory*: specifying the mechanism or process that would produce such a relationship, why environmental variation of a particular kind, measured at a particular scale, should influence a particular biological response in a particular taxon. The distinction matters because each question demands different study designs, different metrics, and different statistical approaches. Most studies in our synthesis address the first two questions reasonably well; they characterize EH and test its association with a response variable, but only a minority approach the third. The framework we propose below is intended to bridge this gap by making explicit the hypotheses that link EH measurement to biological mechanism.

### 4.1 Environmental Heterogeneity as a Pervasive Driver of Biodiversity

The patterns identified in our synthesis indicate that environmental heterogeneity (EH) has become an increasingly prominent framework in biodiversity research over the past two decades (Stein et al. 2014, 2015). Across taxa, regions, and biological disciplines, most studies reported significant associations between EH and biological variables. The consistency of detected effects – despite substantial variation in definitions, metrics, and analytical approaches‐ supports the view that EH represents a robust and general driver of biodiversity patterns (Stein et al. 2014; Deák et al. 2021; Wan et al. 2023). Rather than being restricted to specific taxonomic groups or ecosystems, EH appears to operate across multiple levels of biological organization, from community structure to genetic variation.

### 4.2 Conceptual Ambiguity and Methodological Diversity

Although EH research has expanded considerably, conceptual ambiguity around EH was previously documented (Stein and Kreft 2015); our synthesis extends this finding, showing that definitional ambiguity has not resolved over the subsequent decade and documenting its structural forms and inferential consequences. Only a minority of studies explicitly defined environmental heterogeneity, and terminology was often used interchangeably with related concepts such as environmental complexity or habitat diversity. This lack of definitional clarity likely contributes to the wide diversity of quantification methods observed across studies. Importantly, methodological diversity itself is not inherently problematic; rather, the absence of conceptual transparency—and the consequent inability to assess whether the methods used are appropriate for the hypothesis being tested—limits comparability and cumulative synthesis. A parallel effort in beta‐diversity research, where Jost (2007) and Chao et al. (2012) imposed conceptual order on a similarly fragmented literature, illustrates the value of explicit frameworks linking terminology, measurement, and mechanism. We therefore advocate for a unified yet flexible definition of EH that emphasizes variation in environmental characteristics across spatial scales while allowing methodological innovation tailored to specific research questions (Levin 1992; Stein et al. 2014).

The framework presented in Table 2 clarifies that much of the variability observed across studies does not simply reflect methodological heterogeneity, but rather differences in how environmental heterogeneity is operationally interpreted. Quantification methods implicitly define which dimension of environmental variation is being captured, thereby shaping the biological processes that may be inferred (Wiens 1989; Austin 2007). For instance, metrics based on statistical dispersion primarily describe environmental variability, whereas indices derived from categorical data characterize habitat diversity or compositional heterogeneity (Tuomisto 2010). Similarly, distance‐based approaches quantify environmental differentiation among sites rather than local variability (Legendre and Legendre 2012).

These distinctions indicate that EH metrics are not interchangeable, as different measurement strategies correspond to distinct ecological questions and mechanisms (Tuomisto 2010; Stein and Kreft 2015). Treating alternative quantification methods as equivalent representations of heterogeneity may obscure underlying processes and contribute to apparently inconsistent empirical findings. Much of the conceptual fragmentation surrounding EH may therefore arise from differences in operational interpretation rather than purely methodological variation.

Importantly, each operational interpretation of EH implicitly invokes different biological mechanisms. Environmental variability metrics are commonly associated with processes such as environmental filtering, selection pressures, phenotypic plasticity, or adaptive responses (Tews et al. 2003; Valladares et al. 2014). In contrast, habitat diversity measures align more closely with niche availability, resource diversification, and coexistence dynamics (Chesson 2000; Tews et al. 2003). Distance‐based measures emphasize turnover and sorting processes across environmental gradients (Legendre and Legendre 2012), while structural proxies are often linked to spatial niche partitioning and resource stratification (Tews et al. 2003). Recognizing these mechanistic distinctions helps explain why EH‐response relationships vary widely across studies and why no single metric can universally capture the multidimensional nature of heterogeneity (Stein et al. 2014).

This conceptual structure also contextualizes our findings regarding analytical complexity. Failure to detect EH effects is unlikely to reflect insufficient analytical complexity per se—as our results confirm, complexity level does not predict effect detection (Low = 90.6%, Medium = 90.9%, High = 97.1%; χ^2^ = 3.07, *P* = 0.21). Rather, inferential limitations more commonly reflect mismatches among conceptual interpretation, measurement scale, and hypothesized mechanisms (Wiens 1989; Levin 1992). Explicit alignment between EH definitions, quantification methods, and expected response patterns is therefore critical for strengthening inference and advancing predictive ecological theory (Austin 2007).

### 4.3 Taxonomic, Geographic, and Disciplinary Biases

The pronounced taxonomic and geographic biases identified in our results highlight structural asymmetries in EH research, consistent with other patterns documented in biodiversity and biogeography studies (Mittermeier et al. 1997; Raven et al. 2020). Plants ‐particularly angiosperms‐ and terrestrial ecosystems dominate the literature, whereas fungi, microorganisms, and many animal groups remain underrepresented. Similarly, research effort is concentrated in the Americas and parts of Europe, with Africa and large areas of Asia receiving comparatively little attention. These patterns likely reflect inequalities in research funding, infrastructure, and data availability, but they also preclude generalizations about EH‐biodiversity relationships globally. Expanding EH research to underrepresented taxa, ecosystems, and regions will be essential for developing a more comprehensive understanding of how environmental variability shapes biodiversity.

### 4.4 Analytical Complexity and Inferential Power

One of the most notable findings of our review concerns the relationship between analytical complexity and the detection of EH effects, echoing concerns raised in other areas of ecological modeling regarding diminishing returns of increasing model complexity (Kéry and Royle 2016; Auger‐Méthé et al. 2021). Contrary to the expectation that more complex analytical designs might yield stronger or more consistent results, we found no evidence that increasing complexity enhanced the probability of detecting significant EH effects. Simpler studies frequently reported significant relationships, suggesting that inferential strength depends more on clearly defined hypotheses, biologically meaningful variables, and appropriate spatial scales than on analytical breadth alone.

Importantly, this finding should not be interpreted as discouraging methodological innovation; rather, it emphasizes that complexity should be aligned with explicit hypotheses about the expected form and scale of EH‐response relationships. Environmental heterogeneity is frequently assumed to exert positive effects on biological responses (Tews et al. 2003). However, theoretical and empirical work indicates that threshold effects, saturation dynamics, or context‐dependent negative relationships may arise when heterogeneity reflects fragmentation, instability, or area‐heterogeneity trade‐offs (Allouche et al. 2012; Stein et al. 2014). Failure to test nonlinear, asymptotic, and scale‐dependent responses may therefore limit inferential power more than insufficient model complexity per se (Wiens 1989; Levin 1992; Austin 2007). Explicitly evaluating alternative functional forms and scale‐dependent dynamics is therefore essential for strengthening inference and advancing predictive ecological theory.

### 4.5 Integrating Abiotic and Biotic Dimensions of EH

Our results show that abiotic components of EH ‐particularly climatic, topographic, and edaphic variability‐ are more frequently analyzed than biotic components, a pattern also observed in studies emphasizing geodiversity and physical drivers of biodiversity (Comer et al. 2015; Hjort et al. 2015). Yet ecological and evolutionary processes are often shaped by interactions among biotic conditions, resource availability, and species interactions. Integrating multiple dimensions of EH, including biotic heterogeneity such as vegetation structure or resource diversity, may therefore provide a more mechanistic understanding of biodiversity responses. Future research that combines abiotic and biotic heterogeneity within coherent analytical frameworks could help bridge ecological and evolutionary perspectives.

### 4.6 Implications for Eco‐Evolutionary Research and Conservation

By documenting consistent associations between EH and ecological, morphological, physiological, and genetic traits, our synthesis underscores the potential of EH to link ecological patterns with evolutionary processes such as local adaptation, phenotypic plasticity, and genetic differentiation (Valladares et al. 2014; Nikzat et al. 2021; Dauphin et al. 2023). Moreover, the geographic and environmental structuring of EH makes it particularly relevant for conservation planning. Heterogeneous landscapes may function as biodiversity reservoirs or climatic refugia under rapid environmental change (Hjort et al. 2015; Starko et al. 2019; Wan et al. 2023). Incorporating explicit measures of EH into biodiversity monitoring and conservation prioritization frameworks may therefore improve predictions of species persistence and ecosystem resilience.

### 4.7 Toward an Operational Definition of Environmental Heterogeneity

A recurring pattern in our synthesis is that the vast majority of studies report significant associations between EH and biological responses without specifying the mechanism or process that would explain why such an association should exist. This omission is not trivial: without an explicit hypothesis, it is impossible to determine whether the absence of a significant effect reflects the true absence of an EH effect, a mismatch between the scale of measurement and the scale of perception of the focal organism, the measurement of irrelevant environmental attributes, or insufficient statistical power. Wiens (1989) made this point with particular clarity: environmental heterogeneity is a human construct, perceived at human scales—what appears heterogeneous to a researcher using 1‐km^2^ grid cells may be functionally homogeneous for a mite, and what appears homogeneous at landscape scale may be highly heterogeneous for a soil invertebrate at the grain of centimeters.

**FIGURE 10:**
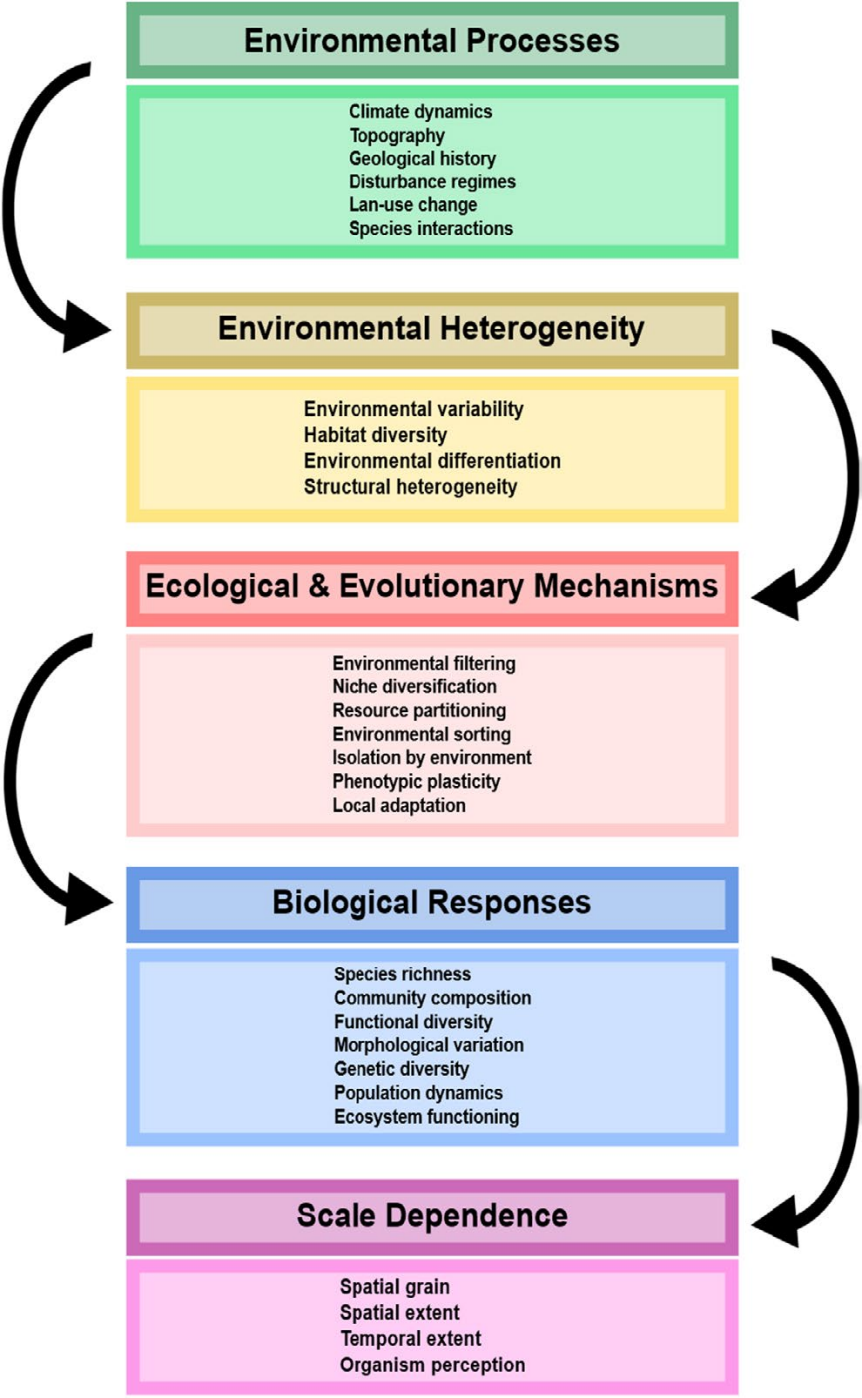
Conceptual framework linking environmental heterogeneity (EH) to biological responses. Environmental processes generate different forms of heterogeneity, including environmental variability, habitat diversity, environmental differentiation, and structural heterogeneity. These dimensions do not directly produce biological patterns; rather, they influence ecological and evolutionary mechanisms such as environmental filtering, niche diversification, resource partitioning, isolation by environment, phenotypic plasticity, and local adaptation. These mechanisms subsequently shape biodiversity patterns across multiple levels of biological organization. Scale dependence mediates all stages of the framework, as the ecological relevance of environmental variation depends on the spatial and temporal scales at which organisms perceive and respond to their environment.

To synthesize the relationships identified throughout our review, we propose a conceptual framework linking environmental processes, environmental heterogeneity, ecological and evolutionary mechanisms, and biological responses (Figure 10). This framework emphasizes that EH does not directly generate biological patterns but rather influences biodiversity through multiple ecological and evolutionary pathways whose effects are inherently scale‐dependent. Figure 10 summarizes these conceptual linkages and highlights how environmental variation can translate into biological responses through distinct mechanisms.

Building upon this framework, we suggest that many EH studies can be interpreted within five broad mechanistic hypotheses, although these hypotheses are often invoked implicitly rather than stated explicitly (Table 3; see also MacArthur and Wilson 1967; Wiens 1989; Chesson 2000; Levin 1992; Schluter 2000). Table 3 translates the general pathways depicted in Figure 10 into a set of testable hypotheses, associated predictions, and analytical approaches that can facilitate hypothesis‐driven research on environmental heterogeneity:

- The niche diversification hypothesis proposes that EH generates a diversity of potential ecological niches within a given area, promoting species coexistence through resource partitioning and reducing competitive exclusion. The testable prediction is that sites with higher EH should support more species with non‐overlapping resource use, and that this effect should be strongest when EH is measured at the grain relevant to the organism's home range or foraging behavior (MacArthur and Wilson 1967; Chesson 2000).
- The environmental filtering hypothesis proposes that EH determines which species can establish and persist in a given site by imposing abiotic tolerances as a filter on the regional species pool. The prediction is that community composition should be more deterministic (less stochastic) in sites with strong environmental gradients (used here as a proxy for high Environmental Variability), and that trait‐based analyses should reveal a non‐random match between species' environmental tolerances and local conditions (Kraft et al. 2014; HilleRisLambers et al. 2012).
- The spatial isolation hypothesis proposes that EH creates a mosaic of ecologically distinct patches that function as barriers and corridors, promoting population isolation and ultimately genetic differentiation or speciation. The prediction is that genetic distance among populations should increase with environmental dissimilarity among sites, independently of geographic distance—a pattern testable with isolation‐by‐environment (IBE) analyses using partial Mantel tests or distance‐based redundancy analysis (Wang and Bradburd 2014; Sexton et al. 2013).
- The phenotypic plasticity hypothesis proposes that organisms inhabiting heterogeneous environments experience more variable selection pressures, favoring the evolution of broader reaction norms and greater phenotypic plasticity. The prediction is that populations from more heterogeneous environments should show greater plasticity in morphological, physiological, or behavioral traits, and that plasticity should be heritable rather than solely environmentally induced (Pigliucci 2001; Valladares et al. 2014).
- The scale‐dependent perception hypothesis (Wiens 1989; Levin 1992) proposes that the ecological relevance of EH is not an intrinsic property of the landscape but depends on the spatial grain at which the focal organism perceives and responds to environmental variation. This hypothesis does not make predictions about the direction of the EH–diversity relationship, but rather predicts that effect size and significance should vary systematically with the match between measurement grain and organism‐specific perception scale—a relationship testable through multi‐scale analyses or by using organism‐based heterogeneity metrics derived from movement or foraging data (Wiens 1989; Levin 1992).

**TABLE 3:** Research roadmap linking EH measurement to explicit biological hypotheses (*n* = 360 studies reviewed).

Hypothesis	EH interpretation	EH component measured	Recommended metric	Relevant spatial scale	Statistical approach	Biological mechanism	Testable prediction	Example study
Niche diversification	*Habitat Diversity*	Land cover, Vegetation, Topographic	Habitat type richness, SHDI (Fragstats), habitat patch diversity	Home range/local (10^2^–10^4^ m^2^)	Regression, GLM, ordination (RDA/CCA)	EH generates multiple ecological niches → resource partitioning → coexistence → increased alpha diversity	Sites with higher EH support more species with non‐overlapping resource use; effect strongest when EH measured at organism's foraging grain	*Dufour et al.* *2006* *; Udy et al.* *2021*
Environmental filtering	*Environmental Variability*	Climatic, Soil, Aquatic	Standard deviation, range, or CV of abiotic variables (temperature, precipitation, soil pH, nutrients)	Population/community (10^2^–10^5^ m^2^)	Trait‐based regression, RDA with environmental matrix, mixed‐effects models	EH imposes abiotic tolerance thresholds → deterministic community assembly → trait‐environment matching	Community composition more deterministic (lower stochasticity) in high‐EH sites; non‐random trait‐environment correspondence	*Bagchi et al.* *2011* *; Barczyk et al.* *2023*
Spatial isolation (IBE)	*Environmental Differentiation*	Climatic, Topographic, Soil	Environmental distance matrix (Euclidean, Mahalanobis); Bray–Curtis dissimilarity among sites	Landscape/regional (10^4^–10^6^ m^2^)	Partial Mantel test, dbRDA, distance‐based models controlling for geographic distance	EH creates ecological barriers/corridors → population isolation → genetic differentiation → potential speciation	Genetic distance increases with environmental dissimilarity independently of geographic distance (IBE signal)	*Temunović et al.* *2012* *; Myers et al.* *2019*
Phenotypic plasticity	*Environmental Variability*	Climatic, Soil	SD or CV of environmental variables experienced by individuals across life history	Individual/population (1–10^3^ m^2^)	Reaction norm analysis, mixed‐effects models with environment × genotype interaction	Variable selection pressures favor evolution of broad reaction norms → greater plasticity in morphological, physiological, or behavioral traits	Populations from high‐EH environments show greater heritable plasticity; plasticity not fully explained by phenotypic variance alone	*Gianoli and González‐Teuber* *2005* *; Lázaro‐Nogal et al.* *2015*
Scale‐dependent perception (Wiens 1989)	*Structural Heterogeneity*	Any component—but must be measured at organism‐relevant grain	Organism‐based heterogeneity metrics: movement‐derived grain, acoustic complexity, microhabitat sampling at body‐size scale	Organism‐specific grain — must be determined empirically or from movement ecology data	Multi‐scale regression; comparison of effect size across measurement resolutions	EH is not an objective landscape property but a perception‐dependent construct; mismatch between human‐scale measurement and organism‐scale perception produces null results	Effect size and significance vary systematically with measurement grain; effects strongest when grain matches organism's home range or foraging scale	*Sargeant et al.* *2006*; *Graham et al.* *2019*

*Note:* Five hypotheses identified implicitly in the literature are made explicit, each with its recommended EH interpretation, components, metrics, spatial scale, statistical approach, hypothesized biological mechanism, and a falsifiable testable prediction. Hypotheses are not mutually exclusive. The scale‐dependent perception hypothesis (Wiens 1989; Levin 1992) applies as a cross‐cutting constraint on all others: Effect detection is only expected when measurement grain matches the organism‐specific perception scale.

Abbreviations: CV, coefficient of variation; IBE, isolation by environment; SHDI, Shannon's Diversity Index of habitat types.

Explicitly stating these hypotheses has three practical implications. First, it constrains which EH components and metrics are appropriate for a given study: measuring climatic variability with *WorldClim* at 1 km^2^ resolution is adequate for testing niche diversification in birds or mammals, but is likely too coarse for testing phenotypic plasticity in invertebrates or microorganisms. Second, it clarifies what constitutes a meaningful null result: a non‐significant EH effect does not indicate the absence of EH influence, but rather that EH, as measured, at the scale used, for the taxon studied, does not predict the response variable chosen. Third, it opens the way for cumulative science: the same hypotheses can be tested across taxa, regions, and scales, allowing genuinely comparable inference rather than the isolated case‐by‐case correlations that currently dominate the literature.

The diversity of definitions, metrics, and analytical approaches documented in our results reflects both the conceptual richness and the ambiguity surrounding environmental heterogeneity. Rather than proposing a restrictive or prescriptive definition, we build on our synthesis to articulate an operational definition that can facilitate comparability across studies while preserving methodological flexibility.

We define environmental heterogeneity as the spatial and/or temporal variation in abiotic and/or biotic environmental conditions, including structural complexity and habitat configuration, across one or multiple scales, that may influence biological processes at any level of biological organization. This definition is not intended to replace existing interpretations, but to provide a unifying structure that explicitly connects measurement strategies with hypothesized mechanisms. This definition explicitly incorporates four key elements emerging from our review. First, it recognizes that EH can arise from abiotic factors (e.g., climate, topography, soils), biotic factors (e.g., vegetation structure, resource diversity), or their interaction. Second, it integrates both spatial and temporal dimensions, acknowledging that heterogeneity may reflect environmental gradients, structural complexity, or dynamic variability through time. Third, it emphasizes scale‐dependence, recognizing that ecological and evolutionary consequences of EH may differ across spatial grains, extents, and temporal windows, as well as several levels of biological organization, from populations to phyla. Fourth, and critically, it links environmental variation to specific biological processes, moving beyond a purely descriptive characterization of landscapes to a mechanistically informed framework.

By grounding EH in its functional relevance to biodiversity ‐ through processes such as environmental filtering, coexistence, plasticity, and local adaptation‐, this definition provides a flexible yet conceptually structured foundation for future ecological, evolutionary, and conservation‐oriented research.

### 4.8 Future Directions in Environmental Heterogeneity Research

Building on the patterns identified in our synthesis, several priority areas emerge for advancing EH research. First, addressing persistent taxonomic and geographic biases will be critical. Underrepresented groups such as fungi, bryophytes, and microorganisms remain comparatively understudied despite evidence that microbial systems consistently exhibit EH effects. Expanding research into these groups may provide valuable opportunities to test EH effects on biological responses across a broad range of life histories and dispersal strategies (Franklin and Mills 2009; Yuan et al. 2017; Feeser et al. 2018). The framework proposed here provides an explicit basis for testing existing hypotheses regarding the mechanisms through which environmental heterogeneity influences biodiversity, community structure, phenotypic variation, and genetic diversity across scales. By linking EH metrics to specific ecological and evolutionary processes, it also facilitates the development of new hypotheses capable of explaining the broad range of EH–response relationships documented in the literature. Table 3 represents a starting point for this process by outlining testable predictions and analytical approaches that can improve comparability among studies.

Building upon this framework, we suggest that many EH studies can be interpreted within five broad mechanistic hypotheses, although these hypotheses are often invoked implicitly rather than stated explicitly (Table 3). Table 3 translates the general pathways depicted in Figure 10 into a set of testable hypotheses, associated predictions, and analytical approaches that can facilitate hypothesis‐driven research on environmental heterogeneity:

Second, integrating EH into eco‐evolutionary and genomic frameworks represents a promising avenue. Landscape genomics and related approaches offer powerful tools for linking environmental variation with genomic differentiation and adaptive responses. As climate and land‐use change accelerate, incorporating EH into genomic and niche‐based analyses may improve predictions of population persistence and evolutionary trajectories (Valladares et al. 2014; Supple and Shapiro 2018; Dauphin et al. 2023).

Third, methodological refinement remains an important frontier. The development of composite indicators that integrate multiple dimensions of EH could enhance comparability across studies while preserving multidimensionality. Similarly, analytical approaches that account for hierarchical structure and scale dependence, such as mixed‐effects models, may help align statistical inference with the spatial and ecological complexity inherent to EH (Kéry and Royle 2016; Salinas‐Ruiz et al. 2023).

Collectively, these directions emphasize that advancing conceptual clarity, expanding taxonomic and geographic representation, and integrating new methodological approaches will be essential for consolidating environmental heterogeneity as a predictive and unifying framework in biodiversity science.

Future studies may benefit from explicitly stating which dimension of EH is being quantified, which mechanisms are hypothesized, and which response patterns are expected, thereby aligning conceptual interpretation with measurement strategy. Such alignment would enhance comparability, strengthen inference, and facilitate the development of cumulative and predictive EH theory.

## 5 Conclusions

Our review demonstrates that environmental heterogeneity is widely invoked yet remains conceptually fragmented. The diversity of definitions and quantification methods reflects the richness of the concept but constrains comparability, cumulative synthesis, and theoretical development. Importantly, the variability observed across studies does not solely represent methodological heterogeneity; rather, it reflects fundamental differences in how environmental heterogeneity is operationally interpreted.

By systematically organizing existing definitions and linking quantification approaches to distinct ecological and evolutionary mechanisms, we provide a more coherent conceptual foundation for EH research. Our synthesis indicates that alternative EH metrics are not interchangeable, as measurement choices implicitly define the dimension of environmental variation under investigation and the biological processes that may be inferred. Conceptual ambiguity therefore represents not merely a semantic challenge but an inferential one.

Advancing research on environmental heterogeneity requires moving beyond descriptive invocation toward explicit alignment among definitions, mechanisms, quantification strategies, and expected response patterns. Establishing this conceptual clarity is essential for strengthening inference, developing predictive ecological theory, and improving the integration of EH into biodiversity and conservation frameworks under accelerating environmental change. Clarifying how environmental heterogeneity is defined and measured is therefore indispensable for transforming EH from a descriptive concept into a predictive framework.

Ultimately, environmental heterogeneity should be viewed not as a single explanatory variable, but as a multidimensional property of environmental variation whose biological consequences emerge through distinct, scale‐dependent ecological and evolutionary mechanisms.

## Author Contributions

**Iván Hérnandez‐Chávez:** conceptualization (equal), data curation (equal), formal analysis (equal), investigation (equal), methodology (equal), writing – original draft (equal). **Giovani Hernández‐Canchola:** conceptualization (equal), formal analysis (equal), writing – review and editing (equal). **Celia López‐González:** conceptualization (equal), investigation (equal), writing – review and editing (equal). **Livia León‐Paniagua:** conceptualization (equal), supervision (equal), writing – review and editing (equal).

## Funding

This work was supported by Secretaría de Investigación y Posgrado, Instituto Politécnico Nacional (IPN) – grants SIP‐2023‐RE/023 and SIP‐2024‐0163 CLG; Secretaría de Ciencia, Humanidades, Tecnología e Innovación (SECIHTI) – grant CF‐2023‐G‐222; and SEP – grant SPI‐2024‐0163.

## Conflicts of Interest

The authors declare no conflicts of interest.

## Supporting information


**Supporting Information S1:** Bibliographic database of studies on environmental heterogeneity (EH) published between 2000 and 2025 (*n* = 360 articles), with coded variables on biological discipline, study region, taxonomic group, heterogeneity component, quantification method, type of statistical analysis, detection of an EH effect, and a composite quality/relevance index (ACI).

**Supporting Information S2:** PRISMA flow diagram for the semi‐systematic narrative synthesis. Records were retrieved from Web of Science (*n* = 3188) and Scopus (*n* = 2689). After deduplication (2317 duplicates removed), language and date filtering (112 excluded), and two‐stage screening (automated + manual review of 750 records), 360 studies met all inclusion criteria and were retained for data extraction. WoS = Web of Science.

**Supporting Information S3:** Detailed results of environmental heterogeneity effect detection by taxonomic group.

## Data Availability

All data used in this study were derived from published literature. The dataset compiled for this review, including extracted definitions and classification variables of environmental heterogeneity, is available in the Supporting Information accompanying this article.
