# Supplementary material for: What do We Mean by Environmental Heterogeneity? Toward A Mechanistically Explicit and Scale‐Dependent Framework
Source: Ecol Evol. 2026 Aug 14;16(8):e74155. doi: 10.1002/ece3.74155 (PMC13473910; doi:10.1002/ece3.74155)
Supplement: Supplementary file 1 [file ECE3-16-e74155-s001.docx]

**Supporting Information S3. Detailed results of environmental heterogeneity (EH) effect detection by taxonomic group (n = 360 studies).**

**Table S3.1. Frequency of significant EH–response relationships by taxonomic group.**

| **Taxonomic group** | **n studies** | **% of total (n=360)** | **Significant EH effect, n (%)** | **No significant effect, n (%)** | **Predominant EH component(s)** | **Predominant statistical approach** |
| --- | --- | --- | --- | --- | --- | --- |
| Plants | 141 | 39.2% | 128 (90.8%) | 13 (9.2%) | Soil; Topographic | Regression (42.6%) |
| Vertebrates | 75 | 20.8% | 68 (90.7%) | 7 (9.3%) | Vegetation; Aquatic | Regression (50.7%) |
| Invertebrates | 73 | 20.3% | 66 (90.4%) | 7 (9.6%) | Aquatic; Vegetation | Regression (31.5%) |
| Microorganisms | 38 | 10.6% | 38 (100.0%) | 0 (0.0%) | Aquatic; Soil | Ordination/RDA (55.3%) |
| Multi-taxon | 18 | 5.0% | 18 (100.0%) | 0 (0.0%) | Topographic; Vegetation | Regression (44.4%) |
| Other¹ | 15 | 4.2% | 13 (86.7%) | 2 (13.3%) | Not applicable² | Not applicable² |

**Table S3.2. Analytical Complexity Index (ACI) distribution, prevalence of explicit EH definitions, and predominant biological discipline by taxonomic group.**

| **Taxonomic group** | **Low ACI, n** | **Medium ACI, n** | **High ACI, n** | **Studies with explicit EH definition, n (%)** | **Predominant biological discipline** |
| --- | --- | --- | --- | --- | --- |
| Plants | 67 | 46 | 28 | 13 (9.2%) | Ecology (66.0%) |
| Vertebrates | 34 | 26 | 15 | 5 (6.7%) | Ecology (48.0%) |
| Invertebrates | 35 | 23 | 15 | 7 (9.6%) | Ecology (69.9%) |
| Microorganisms | 11 | 21 | 6 | 3 (7.9%) | Ecology (92.1%) |
| Multi-taxon | 8 | 5 | 5 | 2 (11.1%) | Ecology (66.7%) |
| Other¹ | 15 | 0 | 0 | 6 (40.0%) | Ecology (73.3%) |

**Notes:** Percentages for “Significant EH effect” and “No significant effect” are calculated within each taxonomic group (row percentages). Association between taxonomic group and effect detection was not statistically significant (χ² = 6.13, df = 5, p = 0.294), consistent with the pattern reported in the main text (Section 3.1.8). “Predominant EH component(s)” lists the two most frequently recorded components per group; studies could record more than one component. “Predominant statistical approach” and “Predominant biological discipline” list the modal category and its within-group percentage.

¹ The “Other” taxonomic group (n = 15) comprises theoretical and simulation-based studies (e.g., metacommunity models, spatial coexistence models) that were not associated with a specific empirical focal taxon.

² EH component and statistical approach were coded as “Other/Not applicable” for all 15 studies in this group given their theoretical/simulation-based nature, which does not use empirically measured EH components or standard inferential statistics in the same sense as empirical studies.

Multi-taxon studies (n = 18) are those examining two or more taxonomic groups simultaneously (see Methods, Section 2.1) and are reported here as a combined category rather than disaggregated by taxon combination.

*Source data: Supporting Information S1 (bibliographic database of the semi-systematic narrative synthesis, n = 360 studies retrieved from Web of Science and Scopus, 2000–2025).*
