# Supplementary material for: What do We Mean by Environmental Heterogeneity? Toward A Mechanistically Explicit and Scale‐Dependent Framework
Source: Ecol Evol. 2026 Aug 14;16(8):e74155. doi: 10.1002/ece3.74155 (PMC13473910; doi:10.1002/ece3.74155)
Supplement: Supplementary file 2 [file ECE3-16-e74155-s003.docx]

**SUPPORTING INFORMATION**

Supporting Information S1. Bibliographic database of studies on environmental heterogeneity (EH) published between 2000 and 2025 (*n* = 360 articles), with coded variables on biological discipline, study region, taxonomic group, heterogeneity component, quantification method, type of statistical analysis, detection of an EH effect, and a composite quality/relevance index (ACI).

| Year | First_author | Title | DOI | Defines_EH | Biological_discipline | Study_region | Taxonomic_group | EH_component | Quantification_method | Statistical_analysis | EH_effect_detected | Journal | s_eh | s_quant | s_stat | aci | aci_level |
| --- | --- | --- | --- | --- | --- | --- | --- | --- | --- | --- | --- | --- | --- | --- | --- | --- | --- |
| 2000 | Arscott | Aquatic habitat diversity along the corridor of an Alpine floodplain river (fiume Tagliamento, Italy) | NA | No | Ecology | Italy | Invertebrates | Aquatic | Index | Ordination/RDA | Yes | Archiv Fur Hydrobiologie | 1 | 1 | 3 | 5 | Medium |
| 2002 | Schmitz M. | Substrate heterogeneity and number of plant species in Everglades savannas (Florida, USA) | 10.1023/a:1015848300802 | No | Ecology | United States | Plants | Topographic | Mean | Regression | No | Plant Ecology | 1 | 1 | 2 | 4 | Low |
| 2002 | Svenning | Mesoscale distribution of understorey plants in temperate forest (kalo, Denmark): The importance of environment and dispersal | 10.1023/a:1015885321263 | No | Ecology | Denmark | Plants | Soil | Other | Ordination/RDA | Yes | Plant Ecology | 1 | 1 | 3 | 5 | Medium |
| 2002 | Cowling | Heterogeneity, speciation/extinction history and climate: Explaining regional plant diversity patterns in the Cape Floristic region | 10.1046/j.1472-4642.2002.00143.x | No | Biogeography | South Africa | Plants | Topographic | Other | Regression | Yes | Diversity and Distributions | 1 | 1 | 2 | 4 | Low |
| 2002 | Camargo-Ricalde | Community structure of endemic Mimosa species and environmental heterogeneity in a semi-arid Mexican valley | NA | No | Ecology | Mexico | Plants | Soil | Other | Other | Yes | Journal of Vegetation Science | 1 | 1 | 1 | 3 | Low |
| 2003 | Velho | Influence of environmental heterogeneity on the structure of testate amoebae (protozoa, rhizopoda) assemblages in the plankton of the Upper Parana River floodplain, Brazil | 10.1002/iroh.200390011 | No | Ecology | Brazil | Microorganisms | Aquatic | Other | Other | Yes | International Review of Hydrobiology | 1 | 1 | 1 | 3 | Low |
| 2003 | Arnaud | Metapopulation genetic structure and migration pathways in the land snail Helix aspersa:: Influence of landscape heterogeneity | 10.1023/a:1024409116214 | No | Genetics / Population genetics | France | Invertebrates | Land cover | Other | Correlation | Yes | Landscape Ecology | 1 | 1 | 2 | 4 | Low |
| 2003 | Carbonell | Effects of habitat heterogeneity and local adaptation on the body condition of a forest passerinie at the edge of its distributional range | 10.1046/j.0024-4066.2002.00156.x | No | Ecology | Spain | Vertebrates | Climatic | Mean | Regression | Yes | Biological Journal of the Linnean Society | 1 | 1 | 2 | 4 | Low |
| 2003 | Ribas | Tree heterogeneity, resource availability, and larger scale processes regulating arboreal ant species richness | 10.1046/j.1442-9993.2003.01290.x | No | Ecology | Brazil | Invertebrates | Vegetation | Other | Regression | Yes | Austral Ecology | 1 | 1 | 2 | 4 | Low |
| 2004 | Mller | Quantifying geomorphological heterogeneity to assess species diversity of set-aside arable land | 10.1016/j.agee.2004.01.023 | No | Ecology | Germany | Plants | Topographic | Index | Regression | Yes | Agriculture Ecosystems & Environment | 1 | 1 | 2 | 4 | Low |
| 2004 | Gysels | Small-scale clinal variation, genetic diversity and environmental heterogeneity in the marine gobies Pomatoschistus minutus and P-lozanoi (gobiidae, teleostei) | 10.1038/sj.hdy.6800496 | No | Genetics / Population genetics | Belgium | Vertebrates | Aquatic | Other | Other | Yes | Heredity | 1 | 1 | 1 | 3 | Low |
| 2004 | Volis | The influence of space in genetic-environmental relationships when environmental heterogeneity and seed dispersal occur at similar scale | 10.1086/381406 | No | Genetics / Population genetics | Israel | Plants | Topographic | Other | Ordination/RDA | No | American Naturalist | 1 | 1 | 3 | 5 | Medium |
| 2004 | Holt | The importance of habitat heterogeneity, biotic interactions and dispersal in abundance-occupancy relationships | 10.1111/j.0021-8790.2004.00862.x | No | Ecology | United Kingdom | Microorganisms | Other | Other | Other | Yes | Journal of Animal Ecology | 1 | 1 | 1 | 3 | Low |
| 2004 | Flecker | Tropical fishes as biological bulldozers: Density effects on resource heterogeneity and species diversity | 10.1890/03-0194 | No | Ecology | Venezuela | Vertebrates | Aquatic | Index | Other | Yes | Ecology | 1 | 1 | 1 | 3 | Low |
| 2005 | Wijesinghe | Does pattern of soil resource heterogeneity determine plant community structure? an experimental investigation | 10.1111/j.0022-0477.2004.00934.x | No | Ecology | United Kingdom | Plants | Soil | Percentage | ANOVA | Yes | Journal of Ecology | 1 | 1 | 1 | 3 | Low |
| 2005 | Whiteman | Asymmetric reproductive isolation among polymorphic salamanders | 10.1111/j.1095-8312.2005.00537.x | No | Evolutionary biology | United States | Vertebrates | Aquatic | Other | Other | Yes | Biological Journal of the Linnean Society | 1 | 1 | 1 | 3 | Low |
| 2005 | Cimmaruta | Genetic structure and environmental heterogeneity in the European hake (Merluccius Merluccius) | 10.1111/j.1365-294x.2005.02595.x | No | Genetics / Population genetics | Europe | Vertebrates | Aquatic | Other | Correlation | Yes | Molecular Ecology | 1 | 1 | 2 | 4 | Low |
| 2005 | Camargo-Ricalde | Effect of the spatial and seasonal soil heterogeneity over arbuscular mycorrhizal fungal spore abundance in the semi-arid Valley of Tehuacan-Cuicatlan, Mexico. | NA | No | Ecology | Mexico | Microorganisms | Soil | Mean | ANOVA | Yes | Revista de Biologia Tropical | 1 | 1 | 1 | 3 | Low |
| 2005 | Fine | The contribution of edaphic heterogeneity to the evolution and diversity of Burseraceae trees in the western Amazon | NA | No | Evolutionary biology | Peru and Ecuador | Plants | Soil | Other | Other | Yes | Evolution | 1 | 1 | 1 | 3 | Low |
| 2006 | Prado | The bioengineer Perumytilus purpuratus (mollusca: Bivalvia) in central Chile: Biodiversity, habitat Structural complexity and environmental heterogeneity | 10.1017/s0025315406013282 | No | Ecology | Chile | Invertebrates | Other | Index | Other | Yes | Journal of the Marine Biological Association of the United Kingdom | 1 | 1 | 1 | 3 | Low |
| 2006 | Vanbergen | Consequences for host-parasitoid interactions of grazing-dependent habitat heterogeneity | 10.1111/j.1365-2656.2006.01099.x | No | Ecology | Scotland | Invertebrates | Vegetation | Percentage | Regression | Yes | Journal of Animal Ecology | 1 | 1 | 2 | 4 | Low |
| 2006 | Wegmann | Molecular diversity after a range expansion in heterogeneous environments | 10.1534/genetics.106.062851 | No | Genetics / Population genetics | Switzerland | Other | Other | Other | Other | Yes | Genetics | 1 | 1 | 1 | 3 | Low |
| 2007 | Veech | Habitat and climate heterogeneity maintain beta-diversity of birds among landscapes within ecoregions | 10.1111/j.1466-8238.2007.00315.x | No | Biogeography | United States | Vertebrates | Climatic, Topographic | Other | Regression | Yes | Global Ecology and Biogeography | 2 | 1 | 2 | 5 | Medium |
| 2008 | Cintra | Effects of forest heterogeneity on occurrence and abundance of the scale-backed antbird, Hylophylax poecilinotus (aves: Thamnophilidae), in the Amazon west | 10.1590/s0101-81752008000400008 | No | Ecology | Brazil | Vertebrates | Vegetation | Other | Regression | Yes | Revista Brasileira de Zoologia | 1 | 1 | 2 | 4 | Low |
| 2008 | Grewell | Hemiparasites generate environmental heterogeneity and enhance species coexistence in salt marshes | 10.1890/07-0221.1 | No | Ecology | United States | Plants | Soil | Other | Other | Yes | Ecological Applications | 1 | 1 | 1 | 3 | Low |
| 2009 | Pacini | More rich means more diverse: Extending the 'environmental heterogeneity hypothesis' to taxonomic diversity | 10.1016/j.ecolind.2009.01.003 | No | Ecology | Italy | Plants | Other | Index | Correlation | Yes | Ecological Indicators | 1 | 1 | 2 | 4 | Low |
| 2009 | Mandl | Effects of Environmental heterogeneity on species diversity and composition of terrestrial bryophyte assemblages in tropical montane forests of southern Ecuador | 10.1080/17550870903341877 | No | Ecology | Ecuador | Plants | Topographic, Climatic | Other | Ordination/RDA | Yes | Plant Ecology and Diversity | 2 | 1 | 3 | 6 | High |
| 2009 | Davies | Factors controlling community structure in heterogeneous metacommunities | 10.1111/j.1365-2656.2009.01559.x | No | Ecology | United States | Microorganisms | Other | Other | Other | Yes | Journal of Animal Ecology | 1 | 1 | 1 | 3 | Low |
| 2009 | Jiang | Species diversity and productivity: Why do results of diversity-manipulation experiments differ from natural patterns? | 10.1111/j.1365-2745.2009.01503.x | No | Ecology | China | Plants | Other | Other | Other | No | Journal of Ecology | 1 | 1 | 1 | 3 | Low |
| 2009 | Van Etten M.L. | Effects of environmental heterogeneity on the distribution of sexes within and among populations in a gynodioecious species, Geranium maculatum | 10.1111/j.1469-8137.2009.02940.x | Yes | Ecology | United States | Plants | Soil, Climatic | Mean | Correlation | Yes | New Phytologist | 2 | 1 | 2 | 5 | Medium |
| 2009 | Lundholm J.T. | Plant species diversity and environmental heterogeneity: Spatial scale and competing hypotheses | 10.1111/j.1654-1103.2009.05577.x | No | Ecology | Canada | Plants | Other | Other | Other | Yes | Journal of Vegetation Science | 1 | 1 | 1 | 3 | Low |
| 2010 | Shi | Vascular plant species richness on wetland remnants is determined by both area and habitat heterogeneity | 10.1007/s10531-009-9757-5 | Yes | Conservation biology | China | Plants | Aquatic, Topographic | Index | Regression | Yes | Biodiversity and Conservation | 2 | 1 | 2 | 5 | Medium |
| 2010 | Steets J.A. | Heterogeneous selection on trichome production in Alaskan Arabidopsis kamchatica (brassicaceae) | 10.3732/ajb.0900362 | Yes | Evolutionary biology | United States | Plants | Climatic | Percentage | Regression | Yes | American Journal of Botany | 1 | 1 | 2 | 4 | Low |
| 2010 | Gilliam | Spatial heterogeneity of soil nutrients and plant species in herb-dominated communities of contrasting land use | 10.1007/s11258-010-9725-x | No | Ecology | United States | Plants | Soil | Standard deviation | Regression | Yes | Plant Ecology | 1 | 1 | 2 | 4 | Low |
| 2010 | Verberk | Loss of environmental heterogeneity and aquatic macroinvertebrate diversity following large-scale restoration management | 10.1016/j.baae.2010.04.001 | No | Ecology | Netherlands | Invertebrates | Aquatic | Other | Other | Yes | Basic and Applied Ecology | 1 | 1 | 1 | 3 | Low |
| 2010 | Hilrio A. | High diversity of frenulates (Polychaeta: Siboglinidae) in the Gulf of Cadiz mud volcanoes: A DNA taxonomy analysis | 10.1016/j.dsr.2009.10.004 | No | Ecology | Portugal | Invertebrates | Other | Other | Other | Yes | Deep-Sea Research Part I: Oceanographic Research Papers | 1 | 1 | 1 | 3 | Low |
| 2010 | Villaescusa | A close link between bacterial community composition and environmental heterogeneity in maritime Antarctic lakes | 10.2436/20.1501.01.112 | No | Ecology | Antarctica | Microorganisms | Aquatic | Other | Ordination/RDA | Yes | International Microbiology | 1 | 1 | 3 | 5 | Medium |
| 2010 | Parviainen | NDVI-based productivity and heterogeneity as indicators of plant-species richness in boreal landscapes | NA | No | Conservation biology | Finland | Plants | Vegetation | Index | Regression | Yes | Boreal Environment Research | 1 | 1 | 2 | 4 | Low |
| 2011 | Gundale | Resource heterogeneity does not explain the diversity-productivity relationship across a boreal island fertility gradient | 10.1111/j.1600-0587.2011.06853.x | Yes | Ecology | Sweden | Plants | Soil | Standard deviation | Ordination/RDA | No | Ecography | 1 | 1 | 3 | 5 | Medium |
| 2011 | Stevens | Diversity begets diversity: Relative roles of structural and resource heterogeneity in determining rodent community structure | 10.1644/10-mamm-a-117.1 | Yes | Ecology | United States | Vertebrates | Vegetation, Other | Other | Regression | Yes | Journal of Mammalogy | 2 | 1 | 2 | 5 | Medium |
| 2011 | Ferreiro | Effects of macrophyte heterogeneity and food availability on structural parameters of the macroinvertebrate community in a Pampean stream | 10.1007/s10750-010-0599-7 | No | Ecology | Argentina | Invertebrates | Vegetation | Index | Regression | Yes | Hydrobiologia | 1 | 1 | 2 | 4 | Low |
| 2011 | Eilts | Resource heterogeneity, soil fertility, and species diversity: Effects of clonal species on plant communities | 10.1086/659633 | No | Ecology | United States | Plants | Soil | Other | ANOVA | Yes | American Naturalist | 1 | 1 | 1 | 3 | Low |
| 2011 | Lachish | Infection dynamics of endemic malaria in a wild bird population: Parasite species-dependent drivers of spatial and temporal variation in transmission rates | 10.1111/j.1365-2656.2011.01893.x | No | Ecology | United Kingdom | Vertebrates | Aquatic, Climatic | Other | Other | Yes | Journal of Animal Ecology | 2 | 1 | 1 | 4 | Low |
| 2011 | Chen | Relative importance of water, energy, and heterogeneity in determining regional pteridophyte and seed plant richness in China | 10.1111/j.1759-6831.2011.00120.x | No | Biogeography | China | Plants | Topographic, Climatic | Other | Regression | Yes | Journal of Systematics and Evolution | 2 | 1 | 2 | 5 | Medium |
| 2011 | Negro | The effect of local environmental heterogeneity on species diversity of alpine dung beetles (coleoptera: Scarabaeidae) | 10.14411/eje.2011.012 | No | Ecology | Italy | Invertebrates | Topographic, Vegetation | Other | Regression | Yes | European Journal of Entomology | 2 | 1 | 2 | 5 | Medium |
| 2011 | Brandt | Regional and decadal patterns of native and exotic plant coexistence in California grasslands | 10.1890/10-0485.1 | No | Ecology | United States | Plants | Climatic | Other | Ordination/RDA | Yes | Ecological Applications | 1 | 1 | 3 | 5 | Medium |
| 2012 | Vargas | Is genetic structure of the southern pygmy mouse Baiomys musculus (cricetidae) related to human-induced spatial landscape heterogeneity in a tropical dry forest? | 10.1007/s10709-012-9681-6 | No | Genetics / Population genetics | Mexico | Vertebrates | Land cover | Other | Other | No | Genetica | 1 | 1 | 1 | 3 | Low |
| 2012 | Sivadas | Role of environmental heterogeneity in structuring the macrobenthic community in a tropical sandy beach, west coast of India | 10.1007/s10872-011-0099-z | No | Ecology | India | Invertebrates | Aquatic, Soil | Mean | Ordination/RDA | Yes | Journal of Oceanography | 2 | 1 | 3 | 6 | High |
| 2012 | Hermsen | On the rapidity of antibiotic resistance evolution facilitated by a concentration gradient | 10.1073/pnas.1117716109 | No | Evolutionary biology | United States | Microorganisms | Other | Other | Other | Yes | Proceedings of the National Academy of Sciences of the United States of America | 1 | 1 | 1 | 3 | Low |
| 2012 | Allouche | Area-heterogeneity tradeoff and the diversity of ecological communities | 10.1073/pnas.1208652109 | No | Ecology | Spain | Vertebrates | Topographic, Land cover | Index | Regression | Yes | Proceedings of the National Academy of Sciences of the United States of America | 2 | 1 | 2 | 5 | Medium |
| 2012 | Richardson | Fine-scale spatial heterogeneity and incoming seed diversity additively determine plant establishment | 10.1111/j.1365-2745.2011.01948.x | No | Ecology | Canada | Plants | Soil | Percentage | Ordination/RDA | Yes | Journal of Ecology | 1 | 1 | 3 | 5 | Medium |
| 2012 | Knox | Mitochondrial DNA (COI) analyses reveal that amphipod diversity is associated with environmental heterogeneity in deep-sea habitats | 10.1111/j.1365-294x.2012.05729.x | No | Ecology | New Zealand | Invertebrates | Aquatic | Index | Ordination/RDA | Yes | Molecular Ecology | 1 | 1 | 3 | 5 | Medium |
| 2012 | Bittebiere | Morphological response to competition for light in the clonal Trifolium repens (fabaceae) | 10.3732/ajb.1100487 | No | Ecology | France | Plants | Vegetation | Other | ANOVA | Yes | American Journal of Botany | 1 | 1 | 1 | 3 | Low |
| 2012 | De Stefano | Relation between the environmental heterogeneity and birds distribution and the richness in two units of landscapes of the Delta of the Parana, Argentina | NA | Yes | Ecology | Argentina | Vertebrates | Vegetation | Other | Correlation | Yes | Ornitologia Neotropical | 1 | 1 | 2 | 4 | Low |
| 2012 | dos Santos | The role of environmental heterogeneity in maintenance of anuran amphibian diversity of the Brazilian mesophytic semideciduous forest | NA | No | Ecology | Brazil | Vertebrates | Other | Other | Other | Yes | Tropical Forests | 1 | 1 | 1 | 3 | Low |
| 2013 | Laanisto | Microfragmentation concept explains non-positive environmental heterogeneity-diversity relationships | 10.1007/s00442-012-2398-5 | Yes | Ecology | Estonia | Other | Other | Other | Other | No | Oecologia | 1 | 1 | 1 | 3 | Low |
| 2013 | Tamburello | Habitat heterogeneity promotes the coexistence of exotic seaweeds | 10.1007/s00442-012-2510-x | No | Ecology | Italy | Plants | Other | Percentage | ANOVA | Yes | Oecologia | 1 | 1 | 1 | 3 | Low |
| 2013 | Gazol | A negative heterogeneity-diversity relationship found in experimental grassland communities | 10.1007/s00442-013-2623-x | No | Ecology | Estonia | Plants | Soil | Other | ANOVA | No | Oecologia | 1 | 1 | 1 | 3 | Low |
| 2013 | Avolio | Correlations between genetic and species diversity: Effects of resource quantity and heterogeneity | 10.1111/jvs.12042 | No | Ecology | United States | Plants | Soil, Climatic | Standard deviation | Correlation | Yes | Journal of Vegetation Science | 2 | 1 | 2 | 5 | Medium |
| 2013 | de Juan | Counting on Î²-diversity to safeguard the resilience of estuaries | 10.1371/journal.pone.0065575 | No | Ecology | New Zealand | Invertebrates | Aquatic, Soil | Other | Regression | Yes | PLoS ONE | 2 | 1 | 2 | 5 | Medium |
| 2013 | Heino | Environmental heterogeneity and Î² diversity of stream macroinvertebrate communities at intermediate spatial scales | 10.1899/12-083.1 | No | Ecology | Finland | Invertebrates | Aquatic | Other | Ordination/RDA | No | Freshwater Science | 1 | 1 | 3 | 5 | Medium |
| 2013 | Schoolmaster | Resource competition and coexistence in heterogeneous metacommunities: Many-species coexistence is unlikely to be facilitated by spatial variation in resources | 10.7717/peerj.136 | No | Ecology | United States | Other | Other | Other | Other | No | PeerJ | 1 | 1 | 1 | 3 | Low |
| 2014 | Astorga | Habitat heterogeneity drives the geographical distribution of beta diversity: The case of New Zealand stream invertebrates | 10.1002/ece3.1124 | No | Ecology | New Zealand | Invertebrates | Aquatic, Topographic | Other | Regression | Yes | Ecology and Evolution | 2 | 1 | 2 | 5 | Medium |
| 2014 | Redon | Effects of increasing landscape heterogeneity on local plant species richness: How much is enough? | 10.1007/s10980-014-0027-x | No | Conservation biology | France | Plants | Land cover, Topographic | Index | Regression | Yes | Landscape Ecology | 2 | 1 | 2 | 5 | Medium |
| 2014 | Hai N.H. | Spatial distributions of tropical tree species in northern Vietnam under environmentally variable site conditions | 10.1007/s11676-014-0457-y | Yes | Ecology | Vietnam | Plants | Other | Other | Other | Yes | Journal of Forestry Research | 1 | 1 | 1 | 3 | Low |
| 2014 | Liang | Effects of environmental heterogeneity on predictions of tree species' abundance in response to climate warming | 10.1016/j.envsoft.2014.05.025 | No | Ecology | China | Plants | Climatic, Topographic | Other | Other | Yes | Environmental Modelling and Software | 2 | 1 | 1 | 4 | Low |
| 2014 | Satorre | Micro-geographical scale variation in Drosophila melanogaster larval olfactory behaviour is associated with host fruit heterogeneity | 10.1111/eea.12192 | No | Evolutionary biology | Argentina | Invertebrates | Vegetation | Other | ANOVA | Yes | Entomologia Experimentalis et Applicata | 1 | 1 | 1 | 3 | Low |
| 2014 | Jimnez | Diversity anomalies and spatial climate heterogeneity | 10.1111/geb.12181 | No | Biogeography | Eastern Asia, eastern North America, western North America, Cape Floristic Region in Southern Africa, Southwest Australian Floristic Region | Plants | Climatic | Other | Ordination/RDA | Yes | Global Ecology and Biogeography | 1 | 1 | 3 | 5 | Medium |
| 2014 | Souza | Habitat heterogeneity and anuran community of an agroecosystem in the Pantanal of Brazil | 10.11606/issn.2316-9079.v13i1p41-50 | No | Ecology | Brazil | Vertebrates | Aquatic, Vegetation | Other | Other | Yes | Phyllomedusa | 2 | 1 | 1 | 4 | Low |
| 2014 | Aguirre-Len | Environmental heterogeneity and its relationship with diversity and abundance of the fish community in a coastal system of Gulf of Mexico | 10.15517/rbt.v62i1.8212 | No | Ecology | Mexico | Vertebrates | Aquatic, Climatic | Index | Ordination/RDA | Yes | Revista de Biologia Tropical | 2 | 1 | 3 | 6 | High |
| 2014 | McGrann | Heterogeneity in avian richness-environment relationships along the Pacific Crest Trail | 10.5751/ace-00695-090208 | No | Ecology | United States | Vertebrates | Climatic, Topographic | Other | Regression | Yes | Avian Conservation and Ecology | 2 | 1 | 2 | 5 | Medium |
| 2015 | Yang (b) | The effect of environmental heterogeneity on species richness depends on community position along the environmental gradient | 10.1038/srep15723 | Yes | Ecology | China | Other | Other | Other | Other | Yes | Scientific Reports | 1 | 1 | 1 | 3 | Low |
| 2015 | Prasad | Macroscale intraspecific variation and environmental heterogeneity: Analysis of cold and warm zone abundance, mortality, and regeneration distributions of four eastern US tree species | 10.1002/ece3.1752 | No | Ecology | United States | Plants | Climatic, Topographic | Index | Regression | Yes | Ecology and Evolution | 2 | 1 | 2 | 5 | Medium |
| 2015 | Bozelli | Floods decrease zooplankton beta diversity and environmental heterogeneity in an Amazonian floodplain system | 10.1007/s10750-015-2209-1 | No | Ecology | Brazil | Invertebrates | Aquatic | Other | Ordination/RDA | Yes | Hydrobiologia | 1 | 1 | 3 | 5 | Medium |
| 2015 | Wang | Plant diversity is associated with the amount and spatial structure of soil heterogeneity in meadow steppe of China | 10.1007/s10980-013-9955-0 | No | Ecology | China | Plants | Soil | Standard deviation | Regression | Yes | Landscape Ecology | 1 | 1 | 2 | 4 | Low |
| 2015 | del Pino | Light heterogeneity interacts with plant-induced soil heterogeneity to affect plant trait expression | 10.1007/s11258-015-0448-x | Yes | Ecology | United States | Plants | Soil, Vegetation | Other | ANOVA | Yes | Plant Ecology | 2 | 1 | 1 | 4 | Low |
| 2015 | Dek | Micro-topographic heterogeneity increases plant diversity in old stages of restored grasslands | 10.1016/j.baae.2015.02.008 | No | Ecology | Hungary | Plants | Topographic | Other | ANOVA | Yes | Basic and Applied Ecology | 1 | 1 | 1 | 3 | Low |
| 2015 | Yang | Transgenerational plasticity provides ecological diversity for a seed heteromorphic species in response to environmental heterogeneity | 10.1016/j.ppees.2015.03.003 | No | Evolutionary biology | China | Plants | Climatic, Soil | Other | Other | Yes | Perspectives in Plant Ecology, Evolution and Systematics | 2 | 1 | 1 | 4 | Low |
| 2015 | Bar-Massada A. (a) | Complex relationships between species niches and environmental heterogeneity affect species co-occurrence patterns in modelled and real communities | 10.1098/rspb.2015.0927 | No | Ecology | United States | Vertebrates, Plants | Other | Other | Other | Yes | Proceedings of the Royal Society B: Biological Sciences | 1 | 1 | 1 | 3 | Low |
| 2015 | Grman | Altered beta diversity in post-agricultural woodlands: Two hypotheses and the role of scale | 10.1111/ecog.01159 | No | Ecology | United States | Plants | Other | Other | Ordination/RDA | No | Ecography | 1 | 1 | 3 | 5 | Medium |
| 2015 | Bertin | Effects of wind-driven spatial structure and environmental heterogeneity on high-altitude wetland macroinvertebrate assemblages with contrasting dispersal modes | 10.1111/fwb.12488 | No | Ecology | Chile | Invertebrates | Aquatic, Climatic | Other | Ordination/RDA | Yes | Freshwater Biology | 2 | 1 | 3 | 6 | High |
| 2015 | Chocron | Bird diversity and environmental heterogeneity in North America: A test of the area-heterogeneity trade-off | 10.1111/geb.12353 | No | Biogeography | United States, Canada | Vertebrates | Topographic, Land cover | Index | Regression | Yes | Global Ecology and Biogeography | 2 | 1 | 2 | 5 | Medium |
| 2015 | Vleminckx | Impact of fine-scale edaphic heterogeneity on tree species assembly in a central African rainforest | 10.1111/jvs.12209 | No | Ecology | Democratic Republic of the Congo | Plants | Soil | Other | Ordination/RDA | Yes | Journal of Vegetation Science | 1 | 1 | 3 | 5 | Medium |
| 2015 | Song S. | Impacts of environmental heterogeneity on moss diversity and distribution of Didymodon (Pottiaceae) in Tibet, China | 10.1371/journal.pone.0132346 | Yes | Ecology | China | Plants | Topographic, Climatic | Other | Ordination/RDA | Yes | PLoS ONE | 2 | 1 | 3 | 6 | High |
| 2015 | Bilia | Influence of environmental heterogeneity on the attributes of Chironomidae community in neotropical floodplain lakes | 10.1590/1678-4766201510512027 | No | Ecology | Brazil | Invertebrates | Aquatic | Index | Correlation | Yes | Iheringia - Serie Zoologia | 1 | 1 | 2 | 4 | Low |
| 2015 | Gouveia | Effects of habitat size and heterogeneity on anuran breeding assemblages in the Brazilian dry forest | 10.1670/13-220 | No | Ecology | Brazil | Vertebrates | Aquatic | Other | Regression | No | Journal Of Herpetology | 1 | 1 | 2 | 4 | Low |
| 2015 | Martnez | Variability of environmental heterogeneity in northern Patagonia, Chile: Effects on the spatial distribution, size structure and abundance of chlorophyll-a | 10.4067/s0718-19572015000100004 | No | Ecology | Chile | Microorganisms | Aquatic, Climatic | Mean | Other | Yes | Revista de Biologia Marina y Oceanografia | 2 | 1 | 1 | 4 | Low |
| 2015 | Massicotte | Testing the influence of environmental heterogeneity on fish species richness in two biogeographic provinces | 10.7717/peerj.760 | No | Conservation biology | United States | Vertebrates | Aquatic | Other | Regression | Yes | PeerJ | 1 | 1 | 2 | 4 | Low |
| 2015 | Bar-Massada A. (b) | Immigration rates and species niche characteristics affect the relationship between species richness and habitat heterogeneity in modeled meta-communities | 10.7717/peerj.832 | No | Ecology | Israel | Other | Other | Other | Other | Yes | PeerJ | 1 | 1 | 1 | 3 | Low |
| 2016 | Xue | Spatial heterogeneity in soil particle size: Does it affect the yield of plant communities with different species richness? | 10.1093/jpe/rtv082 | Yes | Ecology | China | Plants | Soil | Other | ANOVA | No | Journal of Plant Ecology | 1 | 1 | 1 | 3 | Low |
| 2016 | do Carmo | Effects of fine-scale surface heterogeneity on rock outcrop plant community structure | 10.1111/jvs.12342 | No | Ecology | Brazil | Plants | Topographic | Index | Regression | Yes | Journal of Vegetation Science | 1 | 1 | 2 | 4 | Low |
| 2016 | Jimnez-Alfaro | Disentangling vegetation diversity from climate-energy and habitat heterogeneity for explaining animal geographic patterns | 10.1002/ece3.1972 | No | Biogeography | Europe | Vertebrates, Invertebrates, Plants | Topographic, Vegetation, Climatic | Other | Regression | Yes | Ecology and Evolution | 3 | 1 | 2 | 6 | High |
| 2016 | Salmela | Natural quantitative genetic variance in plant growth differs in response to ecologically relevant temperature heterogeneity | 10.1002/ece3.2482 | No | Evolutionary biology | United States | Plants | Climatic | Other | ANOVA | Yes | Ecology and Evolution | 1 | 1 | 1 | 3 | Low |
| 2016 | Xu | Soil phosphorus heterogeneity promotes tree species diversity and phylogenetic clustering in a tropical seasonal rainforest | 10.1002/ece3.2529 | No | Ecology | China | Plants | Soil | Standard deviation | Correlation | Yes | Ecology and Evolution | 1 | 1 | 2 | 4 | Low |
| 2016 | Shirima | The relative importance of vertical soil nutrient heterogeneity, and mean and depth-specific soil nutrient availabilities for tree species richness in tropical forests and woodlands | 10.1007/s00442-016-3696-0 | No | Ecology | Tanzania | Plants | Soil | Standard deviation | Regression | Yes | Oecologia | 1 | 1 | 2 | 4 | Low |
| 2016 | Hauffe | Environmental heterogeneity predicts species richness of freshwater mollusks in sub-Saharan Africa | 10.1007/s00531-014-1109-3 | No | Biogeography | Sub-Saharan Africa | Invertebrates | Aquatic, Climatic | Other | Regression | Yes | International Journal of Earth Sciences | 2 | 1 | 2 | 5 | Medium |
| 2016 | Naveda-Rodrguez | Geographic patterns of species richness of diurnal raptors in Venezuela | 10.1007/s10531-016-1102-1 | No | Conservation biology | Venezuela | Vertebrates | Topographic, Vegetation, Land cover | Other | Regression | Yes | Biodiversity and Conservation | 3 | 1 | 2 | 6 | High |
| 2016 | Fernndez | Influence of environmental heterogeneity on the distribution and persistence of a subterranean rodent in a highly unstable landscape | 10.1007/s10709-016-9937-7 | No | Genetics / Population genetics | Argentina | Vertebrates | Soil, Climatic | Other | Other | Yes | Genetica | 2 | 1 | 1 | 4 | Low |
| 2016 | Burgess | Does the response of bird assemblages to fire mosaic properties vary among spatial scales and foraging guilds? | 10.1007/s10980-015-0275-4 | No | Conservation biology | Australia | Vertebrates | Topographic, Land cover | Other | Regression | Yes | Landscape Ecology | 2 | 1 | 2 | 5 | Medium |
| 2016 | Oricchio | Distinct community dynamics at two artificial habitats in a recreational marina | 10.1016/j.marenvres.2016.09.010 | No | Ecology | Brazil | Invertebrates | Aquatic | Other | Other | Yes | Marine Environmental Research | 1 | 1 | 1 | 3 | Low |
| 2016 | Ampoorter | Driving mechanisms of overstorey-understorey diversity relationships in European forests | 10.1016/j.ppees.2016.02.001 | No | Ecology | Finland, Germany, Italy, Poland, Romania, Spain | Plants | Vegetation | Other | Ordination/RDA | No | Perspectives in Plant Ecology, Evolution and Systematics | 1 | 1 | 3 | 5 | Medium |
| 2016 | Lorenzn | Habitat heterogeneity drives bird species richness, nestedness and habitat selection by individual species in fluvial wetlands of the Parana River, Argentina | 10.1111/aec.12375 | No | Ecology | Argentina | Vertebrates | Vegetation, Aquatic | Index | Regression | Yes | Austral Ecology | 2 | 1 | 2 | 5 | Medium |
| 2016 | Maloufi | Environmental heterogeneity among lakes promotes hyper -diversity across phytoplankton communities | 10.1111/fwb.12731 | No | Ecology | France | Microorganisms | Aquatic | Other | Ordination/RDA | Yes | Freshwater Biology | 1 | 1 | 3 | 5 | Medium |
| 2016 | Arellano | Oligarchic patterns in tropical forests: Role of the spatial extent, environmental heterogeneity and diversity | 10.1111/jbi.12653 | No | Ecology | Bolivia | Plants | Soil, Topographic | Other | Regression | Yes | Journal of Biogeography | 2 | 1 | 2 | 5 | Medium |
| 2016 | Tamme | Co-occurring grassland species vary in their responses to fine-scale soil heterogeneity | 10.1111/jvs.12431 | No | Ecology | Estonia | Plants | Soil | Other | ANOVA | Yes | Journal of Vegetation Science | 1 | 1 | 1 | 3 | Low |
| 2016 | McIntosh | Understory plant community composition is associated with fine-scale above- and below-ground resource heterogeneity in mature lodgepole pine (pinus contorta) forests | 10.1371/journal.pone.0151436 | No | Ecology | Canada | Plants | Soil, Vegetation | Other | Ordination/RDA | Yes | PLoS ONE | 2 | 1 | 3 | 6 | High |
| 2016 | Silva | Environmental heterogeneity and natural regeneration in an Alluvial mixed Araucaria Forest | 10.18671/scifor.v44n112.01 | No | Ecology | Brazil | Plants | Aquatic, Soil, Topographic | Other | Ordination/RDA | Yes | Scientia Forestalis | 3 | 1 | 3 | 7 | High |
| 2016 | Whalen | Grazer diversity interacts with biogenic habitat heterogeneity to accelerate intertidal algal succession | 10.1890/15-1633.1 | No | Ecology | United States | Invertebrates, Plants | Other | Percentage | ANOVA | Yes | Ecology | 1 | 1 | 1 | 3 | Low |
| 2017 | Schuler | Habitat size modulates the influence of heterogeneity on species richness patterns in a model zooplankton community | 10.1002/ecy.1833 | Yes | Ecology | United States | Invertebrates | Aquatic | Other | ANOVA | Yes | Ecology | 1 | 1 | 1 | 3 | Low |
| 2017 | Manenti | Environmental heterogeneity does not affect levels of phenotypic plasticity in natural populations of three Drosophila species | 10.1002/ece3.2904 | No | Evolutionary biology | Italy, Denmark | Invertebrates | Climatic | Other | Other | No | Ecology and Evolution | 1 | 1 | 1 | 3 | Low |
| 2017 | van Schalkwyk | Wide corridors with much environmental heterogeneity best conserve high dung beetle and ant diversity | 10.1007/s10531-017-1299-7 | No | Conservation biology | South Africa | Invertebrates | Topographic, Vegetation | Other | Regression | Yes | Biodiversity and Conservation | 2 | 1 | 2 | 5 | Medium |
| 2017 | Bezerra | Fish diversity in tidepools: Assembling effects of environmental heterogeneity | 10.1007/s10641-017-0584-3 | No | Ecology | Brazil | Vertebrates | Aquatic | Other | Ordination/RDA | Yes | Environmental Biology of Fishes | 1 | 1 | 3 | 5 | Medium |
| 2017 | Aisen | Environmental heterogeneity, not distance, structures montane epigaeic spider assemblages in north-western Patagonia (Argentina) | 10.1007/s10841-017-0034-8 | No | Ecology | Argentina | Invertebrates | Topographic, Vegetation, Climatic | Other | Ordination/RDA | Yes | Journal of Insect Conservation | 3 | 1 | 3 | 7 | High |
| 2017 | Cunha | Impacts of oil palm plantations on changes in environmental heterogeneity and Heteroptera (gerromorpha and nepomorpha) diversity | 10.1007/s10841-017-9959-1 | No | Conservation biology | Brazil | Invertebrates | Aquatic, Land cover | Other | Ordination/RDA | Yes | Journal of Insect Conservation | 2 | 1 | 3 | 6 | High |
| 2017 | Thistle D. | Influence of habitat heterogeneity on the community structure of deep-sea harpacticoid communities from a Canyon and an escarpment site on the continental rise off california | 10.1016/j.dsr.2017.03.005 | Yes | Ecology | United States | Invertebrates | Aquatic, Soil | Other | Other | Yes | Deep-Sea Research Part I: Oceanographic Research Papers | 2 | 1 | 1 | 4 | Low |
| 2017 | David O. | Adaptive diversification in heterogeneous environments | 10.1016/j.tpb.2016.11.003 | Yes | Evolutionary biology | France | Other | Other | Other | Other | Yes | Theoretical Population Biology | 1 | 1 | 1 | 3 | Low |
| 2017 | Keppeler | Influence of protected areas on fish assemblages and fisheries in a large tropical River | 10.1017/s0030605316000247 | No | Conservation biology | Brazil | Vertebrates | Aquatic | Other | Ordination/RDA | Yes | Oryx | 1 | 1 | 3 | 5 | Medium |
| 2017 | Angeletti | Environmental heterogeneity promotes microgeographic genetic divergence in the Mediterranean killifish Aphanius fasciatus (cyprinodontidae) | 10.1080/03949370.2016.1188159 | No | Genetics / Population genetics | Italy | Vertebrates | Aquatic | Other | Other | Yes | Ethology Ecology and Evolution | 1 | 1 | 1 | 3 | Low |
| 2017 | Alves-de-Souza | Does environmental heterogeneity explain temporal Î² diversity of small eukaryotic phytoplankton? example from a tropical eutrophic coastal Lagoon | 10.1093/plankt/fbx026 | No | Ecology | Brazil | Microorganisms | Aquatic, Climatic | Other | Correlation | Yes | Journal of Plankton Research | 2 | 1 | 2 | 5 | Medium |
| 2017 | Corcos | Environmental heterogeneity effects on predator and parasitoid insects vary across spatial scales and seasons: A multi-taxon approach | 10.1111/icad.12249 | No | Ecology | Italy | Invertebrates | Land cover, Vegetation | Other | Regression | Yes | Insect Conservation and Diversity | 2 | 1 | 2 | 5 | Medium |
| 2017 | Zhang | Spatial association and optimum adjacent distribution of trees in a mixed coniferous-broadleaf forest in northeastern China | 10.15666/aeer/1503_15511564 | No | Ecology | China | Plants | Other | Other | Other | Yes | Applied Ecology And Environmental Research | 1 | 1 | 1 | 3 | Low |
| 2017 | Pelez O.E. | Environmental heterogeneity explains species turnover but not nestedness in fish assemblages of a Neotropical basin; [a heterogeneidade ambiental explica o turnover de espÃ©cies, mas nÃ£o seu aninhamento em assembleias de peixes de uma bacia Neotropical] | 10.1590/s2179-975x8616 | No | Ecology | Brazil | Vertebrates | Aquatic | Other | Ordination/RDA | Yes | Acta Limnologica Brasiliensia | 1 | 1 | 3 | 5 | Medium |
| 2017 | Aranda | Preliminary study of species-area, isolation and impact of environmental heterogeneity on insect communities in natural patches in the Brazilian Pantanal | NA | No | Ecology | Brazil | Invertebrates | Vegetation, Land cover | Other | Regression | Yes | Entomological News | 2 | 1 | 2 | 5 | Medium |
| 2017 | Stef?nescu | The importance of climate, productivity, habitat heterogeneity, and human impact on the distribution of bird of prey species richness across two continents latitudinal gradient (Europe and Africa) | NA | No | Biogeography | Europe and Africa | Vertebrates | Topographic, Land cover, Climatic | Other | Regression | Yes | North-Western Journal Of Zoology | 3 | 1 | 2 | 6 | High |
| 2018 | Curd | Bacterial diversity is positively correlated with soil heterogeneity | 10.1002/ecs2.2079 | No | Ecology | United States | Microorganisms | Soil, Vegetation | Standard deviation | Correlation | Yes | Ecosphere | 2 | 1 | 2 | 5 | Medium |
| 2018 | Hammill | Landscape heterogeneity strengthens the relationship between Î²-diversity and ecosystem function | 10.1002/ecy.2492 | No | Ecology | Canada | Microorganisms, Plants | Aquatic, Climatic | Other | Regression | Yes | Ecology | 2 | 1 | 2 | 5 | Medium |
| 2018 | Fajardo | Canopy cover type, and not fine-scale resource availability, explains native and exotic species richness in a landscape affected by anthropogenic fires and posterior land-use change | 10.1007/s10530-017-1539-5 | No | Ecology | Chile | Plants | Vegetation, Soil | Other | Regression | No | Biological Invasions | 2 | 1 | 2 | 5 | Medium |
| 2018 | Lee | Crop heterogeneity and non-crop vegetation can enhance avian diversity in a tropical agricultural landscape in southern China | 10.1016/j.agee.2018.06.016 | No | Conservation biology | China | Vertebrates | Vegetation, Land cover | Index | Regression | Yes | Agriculture, Ecosystems and Environment | 2 | 1 | 2 | 5 | Medium |
| 2018 | Garca-Llamas | Landscape heterogeneity as a surrogate of biodiversity in mountain systems: What is the most appropriate spatial analytical unit? | 10.1016/j.ecolind.2017.10.026 | No | Conservation biology | Spain | Vertebrates | Land cover, Topographic | Index | Regression | Yes | Ecological Indicators | 2 | 1 | 2 | 5 | Medium |
| 2018 | Guo | The role of landscape heterogeneity in regulating plant functional diversity under different precipitation and grazing regimes in semi-arid savannas | 10.1016/j.ecolmodel.2018.04.009 | No | Ecology | Germany | Plants | Soil, Climatic | Other | Other | Yes | Ecological Modelling | 2 | 1 | 1 | 4 | Low |
| 2018 | Miserendino | Biotic diversity of benthic macroinvertebrates at contrasting glacier-fed systems in Patagonia mountains: The role of environmental heterogeneity facing global warming | 10.1016/j.scitotenv.2017.11.320 | No | Ecology | Argentina | Invertebrates | Aquatic, Topographic | Other | Ordination/RDA | Yes | Science of the Total Environment | 2 | 1 | 3 | 6 | High |
| 2018 | Liu | Intensive land-use drives regional-scale homogenization of plant communities | 10.1016/j.scitotenv.2018.07.019 | No | Conservation biology | China | Plants | Land cover, Climatic, Topographic | Index | Regression | Yes | Science of the Total Environment | 3 | 1 | 2 | 6 | High |
| 2018 | Jiang | Geographical isolation and environmental heterogeneity contribute to the spatial genetic patterns of Quercus kerrii (fagaceae) | 10.1038/s41437-017-0012-7 | No | Genetics / Population genetics | China | Plants | Climatic, Topographic | Other | Other | Yes | Heredity | 2 | 1 | 1 | 4 | Low |
| 2018 | Wubs | Plant community evenness responds to spatial plant-soil feedback heterogeneity primarily through the diversity of soil conditioning | 10.1111/1365-2435.13017 | No | Ecology | Europe | Plants | Soil | Other | ANOVA | Yes | Functional Ecology | 1 | 1 | 1 | 3 | Low |
| 2018 | Walker | Designed habitat heterogeneity on green roofs increases seedling survival but not plant species diversity | 10.1111/1365-2664.12970 | No | Conservation biology | Canada | Plants | Soil, Climatic | Other | ANOVA | Yes | Journal of Applied Ecology | 2 | 1 | 1 | 4 | Low |
| 2018 | Cai | Geographical gradients in the biodiversity of Chinese freshwater molluscs: Implications for conservation | 10.1111/ddi.12695 | No | Biogeography | China | Invertebrates | Aquatic, Topographic | Index | Regression | No | Diversity and Distributions | 2 | 1 | 2 | 5 | Medium |
| 2018 | Shrestha | The drivers of High Rhododendron diversity in south-west China: Does seasonality matter? | 10.1111/jbi.13136 | No | Biogeography | China | Plants | Topographic, Climatic | Other | Regression | Yes | Journal of Biogeography | 2 | 1 | 2 | 5 | Medium |
| 2018 | McDonald | Effect of migration and environmental heterogeneity on the maintenance of quantitative genetic variation: A simulation study | 10.1111/jeb.13341 | No | Evolutionary biology | Canada | Other | Other | Other | Other | Yes | Journal Of Evolutionary Biology | 1 | 1 | 1 | 3 | Low |
| 2018 | Maas | Bats in a Cerrado landscape of Northern Brazil: Species occurrence, influence of environmental heterogeneity and seasonality, and eight new records for the State of Tocantins | 10.1515/mammalia-2017-0023 | No | Ecology | Brazil | Vertebrates | Vegetation, Climatic | Other | Regression | Yes | Mammalia | 2 | 1 | 2 | 5 | Medium |
| 2018 | Gutierrez | Fish beta diversity responses to environmental heterogeneity and flood pulses are different according to reproductive guild | 10.1590/1982-0224-20180022 | No | Ecology | Brazil | Vertebrates | Aquatic | Other | Regression | Yes | Neotropical Ichthyology | 1 | 1 | 2 | 4 | Low |
| 2018 | Zhang | Leaf venation variation and phenotypic plasticity in response to environmental heterogeneity in parrotia subaequalis (H. t. chang) R. m. hao et H. t. wei, an endemic and endangered tree species from China | 10.3390/f9050247 | No | Physiology / Ecophysiology | China | Plants | Topographic, Climatic | Mean | Regression | Yes | Forests | 2 | 1 | 2 | 5 | Medium |
| 2018 | Arajo | Influence of habitat heterogeneity on anuran diversity in Restinga landscapes of the Parnaiba River delta, northeastern Brazil | 10.3897/zookeys.757.21900 | No | Ecology | Brazil | Vertebrates | Aquatic, Vegetation | Other | Other | Yes | ZooKeys | 2 | 1 | 1 | 4 | Low |
| 2019 | Liu | Unimodal relationship between three-dimensional soil heterogeneity and plant species diversity in experimental mesocosms | 10.1007/s11104-019-03938-w | Yes | Ecology | Belgium | Plants | Soil | Other | ANOVA | Yes | Plant And Soil | 1 | 1 | 1 | 3 | Low |
| 2019 | Catella | Novel insights into how the mean and heterogeneity of abiotic conditions together shape forb species richness patterns in the allegheny plateau ecoregion | 10.1002/ece3.5508 | No | Ecology | United States | Plants | Soil, Topographic, Climatic | Mean, Standard deviation | Regression | Yes | Ecology and Evolution | 3 | 2 | 2 | 7 | High |
| 2019 | Das Gupta | Resource heterogeneity relationship with understory vegetation in post-fire xeric jack pine forests | 10.1007/s10021-018-0277-6 | No | Ecology | Canada | Plants | Soil, Vegetation | Standard deviation | Regression | Yes | Ecosystems | 2 | 1 | 2 | 5 | Medium |
| 2019 | Aguilar-Medrano | Implications of the environmental heterogeneity on the distribution of the fish functional diversity of the Campeche Bank, Gulf of Mexico | 10.1007/s12526-019-00954-y | No | Ecology | Mexico | Vertebrates | Aquatic, Climatic | Other | Ordination/RDA | Yes | Marine Biodiversity | 2 | 1 | 3 | 6 | High |
| 2019 | Leveau | Primary productivity and habitat diversity predict bird species richness and composition along urban-rural gradients of central Argentina | 10.1016/j.ufug.2019.05.011 | No | Ecology | Argentina | Vertebrates | Vegetation, Land cover | Index | Regression | Yes | Urban Forestry and Urban Greening | 2 | 1 | 2 | 5 | Medium |
| 2019 | Willrich | The role of environmental heterogeneity for the maintenance of distinct bird communities in fragmented forests | 10.1080/01584197.2019.1624577 | No | Conservation biology | Brazil | Vertebrates | Topographic, Vegetation | Other | Ordination/RDA | Yes | Emu | 2 | 1 | 3 | 6 | High |
| 2019 | Pelez (b) | Environmental heterogeneity and dispersal limitation explain different aspects of Î²-diversity in neotropical fish assemblages | 10.1111/fwb.13237 | No | Ecology | Brazil | Vertebrates | Aquatic | Other | Ordination/RDA | Yes | Freshwater Biology | 1 | 1 | 3 | 5 | Medium |
| 2019 | Stover | Interactions between soil heterogeneity and freezing: Implications for grassland plant diversity and relative species abundances | 10.1111/gcb.14645 | No | Ecology | Canada | Plants | Soil, Climatic | Other | ANOVA | Yes | Global Change Biology | 2 | 1 | 1 | 4 | Low |
| 2019 | Miller | Local and regional scale habitat heterogeneity contribute to genetic adaptation in a commercially important marine mollusc (Haliotis rubra) from southeastern Australia | 10.1111/mec.15128 | No | Genetics / Population genetics | Australia | Invertebrates | Aquatic, Topographic | Other | Other | Yes | Molecular Ecology | 2 | 1 | 1 | 4 | Low |
| 2019 | Trevail | Environmental heterogeneity amplifies behavioural response to a temporal cycle | 10.1111/oik.05579 | No | Ecology | United Kingdom | Vertebrates | Aquatic, Climatic | Other | Other | Yes | Oikos | 2 | 1 | 1 | 4 | Low |
| 2019 | Scott | Diversity patterns from sequentially restored grasslands support the 'environmental heterogeneity hypothesis' | 10.1111/oik.05877 | No | Ecology | United States | Plants | Soil, Vegetation | Standard deviation | Correlation | Yes | Oikos | 2 | 1 | 2 | 5 | Medium |
| 2019 | Rodrguez | Environmental heterogeneity explains coarse-scale Î²-diversity of terrestrial vertebrates in Mexico | 10.1371/journal.pone.0210890 | No | Biogeography | Mexico | Vertebrates | Topographic, Climatic, Soil, Vegetation | Other | Regression | Yes | PLoS ONE | 3 | 1 | 2 | 6 | High |
| 2019 | Perini M. | The role of environmental heterogeneity in the seed rain pattern | 10.1590/2179-8087.040618 | No | Ecology | Brazil | Plants | Topographic, Climatic | Mean | Correlation | Yes | Floresta e Ambiente | 2 | 1 | 2 | 5 | Medium |
| 2019 | Wan | Spatial environmental heterogeneity may drive functional trait variation in Hydrocotyle vulgaris (Araliaceae), an invasive aquatic plant | 10.3354/ab00716 | No | Ecology | China | Plants | Climatic | Mean | Correlation | Yes | Aquatic Biology | 1 | 1 | 2 | 4 | Low |
| 2019 | Minicante | Habitat heterogeneity and connectivity: Effects on the planktonic protist community structure at two adjacent coastal sites (the Lagoon and the Gulf of Venice, northern Adriatic Sea, Italy) revealed by metabarcoding | 10.3389/fmicb.2019.02736 | No | Ecology | Italy | Microorganisms | Aquatic | Other | Ordination/RDA | Yes | Frontiers in Microbiology | 1 | 1 | 3 | 5 | Medium |
| 2019 | Wang | The community structures of bacterioplankton and their environmental heterogeneity in the surface seawater of the East China Sea | NA | No | Ecology | China | Microorganisms | Aquatic | Other | Ordination/RDA | Yes | Fresenius Environmental Bulletin | 1 | 1 | 3 | 5 | Medium |
| 2020 | Karbstein | Plant intraspecific functional trait variation is related to within-habitat heterogeneity and genetic diversity intrifolium montanuml. | 10.1002/ece3.6255 | No | Genetics / Population genetics | Germany, Austria | Plants | Soil, Topographic | Standard deviation | Regression | Yes | Ecology and Evolution | 2 | 1 | 2 | 5 | Medium |
| 2020 | Li | Noncrop features and heterogeneity mediate overwintering bird diversity in agricultural landscapes of southwest China | 10.1002/ece3.6319 | No | Conservation biology | China | Vertebrates | Vegetation, Land cover, Aquatic | Index | Regression | Yes | Ecology and Evolution | 3 | 1 | 2 | 6 | High |
| 2020 | Swan | Ground-dwelling mammal diversity responds positively to productivity and habitat heterogeneity in a fire-prone region | 10.1002/ecs2.3248 | No | Conservation biology | Australia | Vertebrates | Vegetation, Land cover | Index | Regression | Yes | Ecosphere | 2 | 1 | 2 | 5 | Medium |
| 2020 | Fernndez-Alez | Environmental heterogeneity drives macrophyte beta diversity patterns in permanent and temporary ponds in an agricultural landscape | 10.1007/s00027-020-0694-4 | No | Ecology | Spain | Plants | Aquatic, Soil | Other | Regression | Yes | Aquatic Sciences | 2 | 1 | 2 | 5 | Medium |
| 2020 | Stover (a) | Soil homogenization modifies productivity, nitrogen retention and decomposition in restored grassland | 10.1007/s10021-019-00401-w | No | Ecology | Canada | Plants | Soil, Topographic | Other | ANOVA | Yes | Ecosystems | 2 | 1 | 1 | 4 | Low |
| 2020 | Nunes | Land cover is the main correlate of phytoplankton beta diversity in subtropical coastal shallow lakes | 10.1007/s10452-020-09790-w | No | Ecology | Brazil | Plants | Aquatic, Soil | Other | Regression | Yes | Aquatic Ecology | 2 | 1 | 2 | 5 | Medium |
| 2020 | Barajas-Barbosa | Use of a broad Î²-diversity measure of pelagic ciliate communities for assessing vertical heterogeneity of water columns in the pacific Arctic Ocean | 10.1007/s11356-020-09753-6 | No | Biogeography | Arctic | Plants | Topographic, Climatic | Index | Regression | Yes | Environmental Science and Pollution Research | 2 | 1 | 2 | 5 | Medium |
| 2020 | Cruz | The efficacy of species-area relationship to indicate fragmentation effects varies with grain size and with heterogeneity | 10.1016/j.ecolind.2019.105904 | No | Genetics / Population genetics | Israel | Plants | Climatic, Aquatic | Other | Other | Yes | Ecological Indicators | 2 | 1 | 1 | 4 | Low |
| 2020 | Zhang (a) | Effects of soil nutrient heterogeneity and parasitic plant infection on an experimental grassland community | 10.1016/j.flora.2020.151666 | No | Genetics / Population genetics | China | Plants | Topographic, Climatic | Other | Ordination/RDA | Yes | Flora: Morphology, Distribution, Functional Ecology of Plants | 2 | 1 | 3 | 6 | High |
| 2020 | Salmela | Mammal functional diversity and habitat heterogeneity: Implications for hominin habitat reconstruction | 10.1016/j.jhevol.2020.102853 | No | Evolutionary biology | Africa | Plants | Soil, Climatic | Other | ANOVA | Yes | Journal Of Human Evolution | 2 | 1 | 1 | 4 | Low |
| 2020 | Stover (b) | Heterogeneity-diversity relationships differ between and within trophic levels in temperate forests | 10.1038/s41559-020-1245-z | No | Ecology | Germany | Plants | Soil, Topographic | Other | ANOVA | Yes | Nature Ecology & Evolution | 2 | 1 | 1 | 4 | Low |
| 2020 | Mogilski | Habitat heterogeneity mediates effects of individual variation on spatial species coexistence | 10.1098/rspb.2019.2436 | No | Conservation biology | China | Plants | Soil, Climatic | Other | Other | No | Proceedings Of The Royal Society B-Biological Sciences | 2 | 1 | 1 | 4 | Low |
| 2020 | Silva V.L. | Environmental heterogeneity and population density affect the functional diversity of personality traits in small mammal populations: Functional diversity of personality | 10.1098/rspb.2020.1713 | No | Ecology | United States | Plants | Soil, Climatic | Other | Ordination/RDA | Yes | Proceedings of the Royal Society B: Biological Sciences | 2 | 1 | 3 | 6 | High |
| 2020 | Pollo R. | Effects of herbivore assemblage on the spatial heterogeneity of soil nitrogen in eastern Eurasian steppe | 10.1111/1365-2664.13655 | No | Conservation biology | China | Vertebrates | Vegetation, Land cover | Index | Other | Yes | Journal of Applied Ecology | 2 | 1 | 1 | 4 | Low |
| 2020 | Liu | The relative role of rivers, environmental heterogeneity and species traits in driving compositional changes in southeastern Amazonian bird assemblages | 10.1111/btp.12793 | No | Biogeography | Brazil | Plants | Topographic, Soil, Climatic | Other | Regression | Yes | Biotropica | 3 | 1 | 2 | 6 | High |
| 2020 | Markgraf | Spatial genetic structuring in a widespread wetland plant on a plateau: Effects of elevation-driven geographic isolation and environmental heterogeneity | 10.1111/fwb.13525 | No | Ecology | China | Plants | Land cover, Vegetation | Index | Regression | Yes | Freshwater Biology | 2 | 1 | 2 | 5 | Medium |
| 2020 | Xu | Environmental heterogeneity influences life-form richness and species composition but not species richness of aquatic macrophytes in tropical coastal rivers | 10.1111/fwb.13586 | No | Ecology | Brazil | Microorganisms | Aquatic | Other | Ordination/RDA | Yes | Freshwater Biology | 1 | 1 | 3 | 5 | Medium |
| 2020 | Giladi | Environmental heterogeneity dynamics drive plant diversity on oceanic islands | 10.1111/jbi.13925 | No | Ecology | Global | Plants | Land cover, Topographic | Other | Regression | Yes | Journal of Biogeography | 2 | 1 | 2 | 5 | Medium |
| 2020 | Adomako | Molecular responses to freshwater limitation in the mangrove tree Avicennia germinans (acanthaceae) | 10.1111/mec.15330 | No | Ecology | Brazil | Plants | Soil | Other | ANOVA | No | Molecular Ecology | 1 | 1 | 1 | 3 | Low |
| 2020 | Barr | Genomic insights into adaptation to heterogeneous environments for the ancient relictualcircaeaster agrestis(circaeasteraceae, ranunculales) | 10.1111/nph.16669 | No | Ecology | China | Vertebrates | Vegetation, Land cover | Other | Regression | Yes | New Phytologist | 2 | 1 | 2 | 5 | Medium |
| 2020 | Heidrich | Seedling traits from root to shoot exhibit genetic diversity and distinct responses to environmental heterogeneity within a tree population | 10.1111/oik.06797 | No | Ecology | Finland | Plants, Invertebrates, Vertebrates, Microorganisms | Topographic, Vegetation | Other | Regression | Yes | Oikos | 2 | 1 | 2 | 5 | Medium |
| 2020 | Chen | Legacy effects of soil homogenization on tallgrass prairie restoration: Toward resolved understanding of the relationship between soil heterogeneity and plant species diversity | 10.1111/rec.13049 | No | Ecology | Canada | Other | Other | Other | Other | Yes | Restoration Ecology | 1 | 1 | 1 | 3 | Low |
| 2020 | Mortelliti A. | Effects of local environmental heterogeneity and provenance selection on two direct seeded eucalypt forest species | 10.1111/rec.13255 | No | Ecology | Australia | Vertebrates | Vegetation | Other | Regression | Yes | Restoration Ecology | 1 | 1 | 2 | 4 | Low |
| 2020 | Zhang (b) | Heterogeneity of fern communities in riparian forest remnants from the south Brazilian campos (pampa); [heterogeneidade de comunidades de samambaias em remanescentes de mata ciliar nos campos (pampa) Sul do Brasil] | 10.1590/1519-6984.221124 | No | Ecology | Brazil | Plants | Soil, Climatic | Other | Regression | Yes | Brazilian Journal of Biology | 2 | 1 | 2 | 5 | Medium |
| 2020 | Maximiano | Monitoring of a community of breeding birds in an agroecosystem with high environmental heterogeneity | 10.30456/avo.2020207 | No | Ecology | Italy | Vertebrates | Vegetation, Topographic, Soil | Other | Other | Yes | Avocetta | 3 | 1 | 1 | 5 | Medium |
| 2020 | Wu | The importance of including soil properties when disentangling the drivers of species richness: The case of the alpine genus saxifraga l. in China | 10.3389/fevo.2020.00244 | No | Genetics / Population genetics | China | Plants | Topographic, Climatic | Other | Other | Yes | Frontiers in Ecology and Evolution | 2 | 1 | 1 | 4 | Low |
| 2020 | Costa | Forest landscape heterogeneity increases shrub diversity at the expense of tree seedling diversity in temperate mixedwood forests | 10.3390/f11020160 | No | Ecology | Canada | Microorganisms | Aquatic, Land cover | Other | Regression | Yes | Forests | 2 | 1 | 2 | 5 | Medium |
| 2021 | Velo-Antn | Landscape resistance constrains hybridization across contact zones in a reproductively and morphologically polymorphic salamander | 10.1038/s41598-021-88349-7 | Yes | Genetics / Population genetics | Spain | Vertebrates | Topographic, Climatic | Other | Other | Yes | Scientific Reports | 2 | 1 | 1 | 4 | Low |
| 2021 | Thompson | Scaling up biodiversity-ecosystem functioning relationships: The role of environmental heterogeneity in space and time | 10.1098/rspb.2020.2779 | Yes | Ecology | Canada | Other | Other | Other | Other | Yes | Proceedings of the Royal Society B: Biological Sciences | 1 | 1 | 1 | 3 | Low |
| 2021 | van Mazijk | Environmental heterogeneity explains contrasting plant species richness between the South African Cape and southwestern Australia | 10.1111/jbi.14118 | No | Biogeography | South Africa, Australia | Plants | Topographic, Climatic, Soil | Other | Regression | Yes | Journal of Biogeography | 3 | 1 | 2 | 6 | High |
| 2021 | Dillon | Habitat heterogeneity, temperature, and primary productivity drive elevational gradients in avian species diversity | 10.1002/ece3.7341 | No | Biogeography | United States | Vertebrates | Topographic, Climatic, Vegetation | Other | Regression | Yes | Ecology and Evolution | 3 | 1 | 2 | 6 | High |
| 2021 | Liu (a) | Soil resource availability is much more important than soil resource heterogeneity in determining the species diversity and abundance of karst plant communities | 10.1002/ece3.8285 | No | Ecology | China | Plants | Soil | Standard deviation | Regression | No | Ecology and Evolution | 1 | 1 | 2 | 4 | Low |
| 2021 | Gansfort | Connectivity of communities interacts with regional heterogeneity in driving species diversity: A mesocosm experiment | 10.1002/ecs2.3749 | No | Ecology | Germany | Invertebrates | Climatic | Other | ANOVA | Yes | Ecosphere | 1 | 1 | 1 | 3 | Low |
| 2021 | Palazzo | Temporal dynamics of rotifers' feeding guilds shaped by chlorophyll-a, nitrate, and environmental heterogeneity in subtropical floodplain lakes | 10.1002/iroh.201902037 | No | Ecology | Brazil | Invertebrates | Aquatic | Other | Correlation | Yes | International Review of Hydrobiology | 1 | 1 | 2 | 4 | Low |
| 2021 | Astorg | Different refuge types dampen exotic invasion and enhance diversity at the whole ecosystem scale in a heterogeneous River system | 10.1007/s10530-020-02374-7 | No | Conservation biology | Canada | Vertebrates, Invertebrates | Aquatic | Other | Ordination/RDA | Yes | Biological Invasions | 1 | 1 | 3 | 5 | Medium |
| 2021 | Zamora-Marn | Small ponds support high terrestrial bird species richness in a Mediterranean semiarid region | 10.1007/s10750-021-04552-7 | No | Conservation biology | Spain | Vertebrates | Aquatic, Land cover | Index | Regression | Yes | Hydrobiologia | 2 | 1 | 2 | 5 | Medium |
| 2021 | Nez-Penichet | High diversity of diurnal Lepidoptera associated with landscape heterogeneity in semi-urban areas of loja city, southern Ecuador | 10.1007/s11252-021-01110-w | No | Conservation biology | Ecuador | Invertebrates | Vegetation | Other | Other | Yes | Urban Ecosystems | 1 | 1 | 1 | 3 | Low |
| 2021 | Liu (b) | Heterogeneity-diversity relationships in natural areas of Yunnan, China | 10.1007/s11769-021-1207-7 | No | Conservation biology | China | Plants, Vertebrates | Topographic, Soil, Climatic | Other | Regression | Yes | Chinese Geographical Science | 3 | 1 | 2 | 6 | High |
| 2021 | Agra | Anthropogenic disturbances alter the relationships between environmental heterogeneity and biodiversity of stream insects | 10.1016/j.ecolind.2020.107079 | No | Ecology | Brazil | Invertebrates | Aquatic | Other | Regression | Yes | Ecological Indicators | 1 | 1 | 2 | 4 | Low |
| 2021 | Voelker | The interaction between spatial variation in habitat heterogeneity and dispersal on biodiversity in a zooplankton metacommunity | 10.1016/j.scitotenv.2020.141861 | No | Ecology | United States | Invertebrates | Aquatic | Other | Other | Yes | Science of the Total Environment | 1 | 1 | 1 | 3 | Low |
| 2021 | Nakajima | A strategic sampling design revealed the local genetic structure of cold-water fluvial sculpin: A focus on groundwater-dependent water temperature heterogeneity | 10.1038/s41437-021-00468-z | No | Genetics / Population genetics | Japan | Vertebrates | Aquatic, Climatic | Other | Other | No | Heredity | 2 | 1 | 1 | 4 | Low |
| 2021 | Petsch | Beta diversity of stream insects differs between boreal and subtropical regions, but land use does not generally cause biotic homogenization | 10.1086/712565 | No | Ecology | Brazil, Finland | Invertebrates | Aquatic, Land cover | Other | Ordination/RDA | Yes | Freshwater Science | 2 | 1 | 3 | 6 | High |
| 2021 | Teittinen | Factors influencing the biodiversity of three microbial groups within and among islands of the Baltic Sea | 10.1093/femsec/fiab049 | No | Ecology | Finland | Microorganisms | Aquatic | Other | Correlation | Yes | FEMS Microbiology Ecology | 1 | 1 | 2 | 4 | Low |
| 2021 | Elsen | Contrasting seasonal patterns of relative temperature and thermal heterogeneity and their influence on breeding and winter bird richness patterns across the conterminous united states | 10.1111/ecog.05520 | No | Biogeography | United States | Vertebrates | Topographic, Climatic | Other | Regression | Yes | Ecography | 2 | 1 | 2 | 5 | Medium |
| 2021 | Al | The macroecology of fish migration | 10.1111/geb.13199 | No | Biogeography | Global | Vertebrates | Climatic | Other | Regression | Yes | Global Ecology and Biogeography | 1 | 1 | 2 | 4 | Low |
| 2021 | Pessa | Unveiling the drivers of local dung beetle species richness in the Neotropics | 10.1111/jbi.14043 | No | Ecology | Neotropics | Invertebrates | Soil, Vegetation | Index | Regression | Yes | Journal of Biogeography | 2 | 1 | 2 | 5 | Medium |
| 2021 | Suissa | Mountains, climate and niche heterogeneity explain global patterns of fern diversity | 10.1111/jbi.14076 | No | Biogeography | Global | Plants | Topographic, Climatic | Other | Regression | Yes | Journal of Biogeography | 2 | 1 | 2 | 5 | Medium |
| 2021 | Glick | Tree-base microsites contribute to physical heterogeneity and herb community structure in a temperate deciduous forest | 10.1111/jvs.12943 | No | Ecology | United States | Plants | Topographic, Soil, Vegetation | Other | Other | Yes | Journal of Vegetation Science | 3 | 1 | 1 | 5 | Medium |
| 2021 | Nunez | From tides to nucleotides: Genomic signatures of adaptation to environmental heterogeneity in barnacles | 10.1111/mec.15949 | No | Evolutionary biology | United States, Norway | Invertebrates | Aquatic, Climatic | Other | Other | Yes | Molecular Ecology | 2 | 1 | 1 | 4 | Low |
| 2021 | Pragai | Scale-independence in beetle species diversity-productivity relationship: The importance of heterogeneity arrangement in space | 10.1163/22244662-bja10023 | No | Ecology | Israel | Invertebrates | Climatic, Vegetation | Index | Regression | Yes | Israel Journal of Ecology and Evolution | 2 | 1 | 2 | 5 | Medium |
| 2021 | Batista | The role of spatial heterogeneity in diversity of squamate reptiles in the Atlantic Forest highlands of Southeastern Brazil | 10.1590/0001-3765202120191522 | No | Ecology | Brazil | Vertebrates | Vegetation, Land cover | Other | Ordination/RDA | Yes | Anais da Academia Brasileira de Ciencias | 2 | 1 | 3 | 6 | High |
| 2021 | Prez-Irineo | Opportunistic use of camera traps to assess the diversity of ground-dwelling birds in relation to environmental and anthropogenic variables in Tehuacan-Cuicatlan, oaxaca, Mexico | 10.1676/19-00144 | No | Conservation biology | Mexico | Vertebrates | Vegetation, Aquatic | Index | Regression | Yes | Wilson Journal of Ornithology | 2 | 1 | 2 | 5 | Medium |
| 2021 | Gao | Effects of soil nutrient heterogeneity on the growth and invasion success of alien plants: A multi-species study | 10.3389/fevo.2020.619861 | No | Ecology | China | Plants | Soil | Other | ANOVA | Yes | Frontiers in Ecology and Evolution | 1 | 1 | 1 | 3 | Low |
| 2021 | Chen | Explaining the geographic pattern of plant invasion in 67 nature reserves in China | 10.3389/fevo.2021.655313 | No | Conservation biology | China | Plants | Topographic, Climatic | Other | Regression | Yes | Frontiers in Ecology and Evolution | 2 | 1 | 2 | 5 | Medium |
| 2021 | Luo | Environmental heterogeneity leads to spatial differences in genetic diversity and demographic structure of Acer caudatifolium | 10.3390/plants10081646 | No | Genetics / Population genetics | Taiwan | Plants | Topographic, Climatic | Other | Other | Yes | Plants | 2 | 1 | 1 | 4 | Low |
| 2021 | Chen (b) | Small herbivores and abiotic heterogeneity promote trait variation of a saltmarsh plant in local communities | 10.7717/peerj.12633 | No | Ecology | Netherlands | Plants | Soil, Topographic | Other | Other | Yes | PeerJ | 2 | 1 | 1 | 4 | Low |
| 2021 | Santamara | Small-scale spatial heterogeneity and diversity of plant-flower visitor interactions in high mountains | 10.7818/ecos.2131 | No | Ecology | Spain | Plants, Invertebrates | Vegetation | Other | Other | Yes | Ecosistemas | 1 | 1 | 1 | 3 | Low |
| 2022 | Shinohara | Similar fish species composition despite larger environmental heterogeneity during severe hypoxia in a coastal ecosystem | 10.1002/ece3.8884 | Yes | Ecology | Japan | Vertebrates | Aquatic, Climatic | Other | Other | No | Ecology and Evolution | 2 | 1 | 1 | 4 | Low |
| 2022 | Gentile | Evaluating the correlation between area, environmental heterogeneity, and species richness using terrestrial isopods (Oniscidea) from the Pontine Islands (west Mediterranean) | 10.1007/s13127-021-00523-x | Yes | Biogeography | Italy | Invertebrates | Topographic, Land cover | Other | Regression | Yes | Organisms Diversity and Evolution | 2 | 1 | 2 | 5 | Medium |
| 2022 | Ahumada-Hernndez | Evaluation of fine-scale environmental heterogeneity and its effect on terrestrial mammal diversity in a grassland in the chihuahuan desert | 10.1016/j.jaridenv.2022.104815 | Yes | Ecology | Mexico | Vertebrates | Vegetation | Index | Regression | Yes | Journal of Arid Environments | 1 | 1 | 2 | 4 | Low |
| 2022 | Daza | Diversity behavior in a community model with spatial heterogeneity | 10.1016/j.physleta.2022.128032 | Yes | Ecology | Brazil | Other | Other | Other | Other | Yes | Physics Letters A | 1 | 1 | 1 | 3 | Low |
| 2022 | Thomsen | Heterogeneity within and among co-occurring foundation species increases biodiversity | 10.1038/s41467-022-28194-y | Yes | Ecology | Global | Invertebrates, Plants | Other | Other | ANOVA | Yes | Nature Communications | 1 | 1 | 1 | 3 | Low |
| 2022 | Lv | Environmental heterogeneity affecting community assembly patterns and phylogenetic diversity of three forest communities at mt. Huangshan, China | 10.3390/f13010133 | Yes | Ecology | China | Plants | Topographic, Climatic | Other | Ordination/RDA | Yes | Forests | 2 | 1 | 3 | 6 | High |
| 2022 | Sorfov | Environmental heterogeneity, dispersal mode and habitat specialisation modify within-site beta diversity of spring macroinvertebrates | 10.1002/iroh.202102112 | No | Ecology | Slovakia | Invertebrates | Aquatic | Other | Ordination/RDA | Yes | International Review of Hydrobiology | 1 | 1 | 3 | 5 | Medium |
| 2022 | de Souza | Understanding the effects of environmental heterogeneity on the morphofunctional structure of the phytoplankton community during the hydrological year in an Amazon floodplain lake, Brazil | 10.1007/s10201-021-00685-9 | Yes | Ecology | Brazil | Microorganisms | Aquatic, Climatic | Other | Ordination/RDA | Yes | Limnology | 2 | 1 | 3 | 6 | High |
| 2022 | Mndez-Rojas | How diverse are sun-grown coffee plantations? local and landscape heterogeneity drives Andean rove beetle diversity | 10.1016/j.agee.2022.108122 | No | Ecology | Colombia | Invertebrates | Vegetation, Land cover | Other | Ordination/RDA | Yes | Agriculture, Ecosystems and Environment | 2 | 1 | 3 | 6 | High |
| 2022 | He (a) | Environmental heterogeneity regulates species-area relationships through the spatial distribution of species | 10.1016/j.fecs.2022.100033 | No | Ecology | China | Plants | Soil, Topographic | Other | Regression | Yes | Forest Ecosystems | 2 | 1 | 2 | 5 | Medium |
| 2022 | Kasel | Concurrent assessment of functional types in extant vegetation and soil seed banks informs environmental constraints and mechanisms of plant community turnover in temperate forests of south-eastern Australia | 10.1016/j.foreco.2022.120321 | No | Conservation biology | Australia | Plants | Soil, Vegetation | Other | Regression | Yes | Forest Ecology and Management | 2 | 1 | 2 | 5 | Medium |
| 2022 | Mannaf | The spatial influences of organic farming and environmental heterogeneity on biodiversity in South Australian landscapes | 10.1016/j.jenvman.2022.116414 | No | Conservation biology | Australia | Plants, Vertebrates | Topographic, Land cover, Vegetation | Index | Regression | Yes | Journal of Environmental Management | 3 | 1 | 2 | 6 | High |
| 2022 | Yu | Community assembly and co-occurrence network complexity of pelagic ciliates in response to environmental heterogeneity affected by sea ice melting in the Ross sea, Antarctica | 10.1016/j.scitotenv.2022.155695 | No | Ecology | Antarctica | Microorganisms | Aquatic, Climatic | Other | Ordination/RDA | Yes | Science of the Total Environment | 2 | 1 | 3 | 6 | High |
| 2022 | Kah | Carbonate fabric diversity and environmental heterogeneity in the late Mesoproterozoic era | 10.1017/s0016756821000406 | No | Ecology | Global | Microorganisms | Aquatic, Other | Other | Other | Yes | Geological Magazine | 2 | 1 | 1 | 4 | Low |
| 2022 | Menezes | Sarcosaprophagous flesh flies (Diptera: Sarcophagidae) are less diverse in Amazon forest than mangroves in Northeast Brazil: Preliminary insights about environmental heterogeneity | 10.1080/00222933.2022.2130835 | No | Ecology | Brazil | Invertebrates | Vegetation, Land cover | Other | Other | No | Journal of Natural History | 2 | 1 | 1 | 4 | Low |
| 2022 | Gomes-Gonalves | Relationships between environmental heterogeneity and fish beta diversity in a tropical Bay | 10.1080/17451000.2022.2063902 | No | Ecology | Brazil | Vertebrates | Aquatic, Soil | Other | Correlation | Yes | Marine Biology Research | 2 | 1 | 2 | 5 | Medium |
| 2022 | Lawrence | Restored saltmarshes have low beta diversity due to limited topographic variation, but this can be countered by management | 10.1111/1365-2664.14179 | No | Conservation biology | United Kingdom | Plants | Topographic | Other | Ordination/RDA | Yes | Journal of Applied Ecology | 1 | 1 | 3 | 5 | Medium |
| 2022 | Ren | Scale-dependent species-area relationship: Niche-based versus stochastic processes in a typical subtropical forest | 10.1111/1365-2745.13924 | No | Ecology | China | Plants | Soil, Topographic | Other | Regression | Yes | Journal of Ecology | 2 | 1 | 2 | 5 | Medium |
| 2022 | Miao B.-G. | Diversity begets diversity: Low resource heterogeneity reduces the diversity of nut-nesting ants in rubber plantations | 10.1111/1744-7917.12964 | Yes | Ecology | China | Invertebrates | Vegetation | Other | Regression | Yes | Insect Science | 1 | 1 | 2 | 4 | Low |
| 2022 | Calvo | Correlates of Odonata species composition in Amazonian streams depend on dissimilarity coefficient and oviposition strategy | 10.1111/een.13188 | No | Ecology | Brazil | Invertebrates | Aquatic, Vegetation | Other | Ordination/RDA | Yes | Ecological Entomology | 2 | 1 | 3 | 6 | High |
| 2022 | Qiao | Spatial asynchrony matters more than alpha stability in stabilizing ecosystem productivity in a large temperate forest region | 10.1111/geb.13488 | No | Ecology | China | Plants | Soil, Climatic, Topographic, Vegetation | Other | Regression | Yes | Global Ecology and Biogeography | 3 | 1 | 2 | 6 | High |
| 2022 | Lima | Does environmental heterogeneity explain Î² diversity of estuarine fish assemblages? example from a tropical estuary under the influence of a semiarid climate, Brazil | 10.1371/journal.pone.0273765 | No | Ecology | Brazil | Vertebrates | Aquatic | Other | Ordination/RDA | Yes | PLoS ONE | 1 | 1 | 3 | 5 | Medium |
| 2022 | Shi | The combined effect of surface water and groundwater on environmental heterogeneity reveals the basis of beta diversity pattern in desert oasis communities | 10.1371/journal.pone.0279704 | No | Ecology | China | Plants | Aquatic, Soil | Other | Ordination/RDA | Yes | PLoS ONE | 2 | 1 | 3 | 6 | High |
| 2022 | Kuchenbecker | Community structure of ants (hymenoptera: Formicidae) in an open habitat: The importance of environmental heterogeneity and interspecific interactions | 10.22201/ib.20078706e.2022.93.3900 | No | Ecology | Brazil | Invertebrates | Vegetation | Other | Regression | Yes | Revista Mexicana de Biodiversidad | 1 | 1 | 2 | 4 | Low |
| 2022 | Noble | Canopy cover and canopy heterogeneity drive plant alpha diversity but not beta diversity in a midwest oak savanna | 10.3375/21-55 | No | Ecology | United States | Plants | Vegetation | Index | Regression | Yes | Natural Areas Journal | 1 | 1 | 2 | 4 | Low |
| 2022 | Thakur | Water-energy, climate, and habitat heterogeneity mutually drives spatial pattern of tree species richness in the Indian Western Himalaya | 10.3389/ffgc.2022.1022082 | No | Biogeography | India | Plants | Topographic, Climatic | Other | Regression | Yes | Frontiers In Forests And Global Change | 2 | 1 | 2 | 5 | Medium |
| 2022 | He (b) | Spatial distribution and species association of dominant tree species in Huangguan plot of Qinling mountains, China | 10.3390/f13060866 | No | Ecology | China | Plants | Topographic, Soil | Other | Other | Yes | Forests | 2 | 1 | 1 | 4 | Low |
| 2022 | Lpez-Gonzlez | Mammals of the JesÃºs MarÃ­a River basin, western Mexico: Alpha and beta diversity in an area of high environmental heterogeneity | 10.3398/064.082.0404 | No | Biogeography | Mexico | Vertebrates | Topographic, Vegetation | Other | Other | Yes | Western North American Naturalist | 2 | 1 | 1 | 4 | Low |
| 2022 | Hui | Full-length 16S rrna amplicon sequencing reveals the variation of epibiotic microbiota associated with two shrimp species of Alvinocarididae: Possibly co-determined by environmental heterogeneity and specific recognition of hosts | 10.7717/peerj.13758 | No | Ecology | China | Microorganisms | Aquatic | Other | Ordination/RDA | Yes | PeerJ | 1 | 1 | 3 | 5 | Medium |
| 2023 | Prez-Snchez | Local and landscape environmental heterogeneity drive ant community structure in temperate seminatural upland grasslands | 10.1002/ece3.9889 | Yes | Ecology | Germany | Invertebrates | Soil, Climatic, Land cover | Other | Ordination/RDA | Yes | Ecology and Evolution | 3 | 1 | 3 | 7 | High |
| 2023 | Gamfeldt | Scaling-up the biodiversity-ecosystem functioning relationship: The effect of environmental heterogeneity on transgressive overyielding | 10.1111/oik.09652 | Yes | Ecology | Sweden | Microorganisms, Plants | Other | Other | Other | Yes | Oikos | 1 | 1 | 1 | 3 | Low |
| 2023 | Tardanico | Effects of compositional heterogeneity and spatial autocorrelation on richness and diversity in simulated landscapes | 10.1002/ece3.10810 | No | Ecology | Germany | Other | Other | Other | Other | Yes | Ecology and Evolution | 1 | 1 | 1 | 3 | Low |
| 2023 | de Oliveira | Effect of altitude and spatial heterogeneity on the host-parasite relationship in anurans from a remnant humid forest in the Brazilian semiarid | 10.1007/s00436-023-07965-6 | No | Ecology | Brazil | Vertebrates | Topographic, Climatic | Other | Ordination/RDA | No | Parasitology Research | 2 | 1 | 3 | 6 | High |
| 2023 | Feng | Elevation range and contemporary climate determine the taxonomic, functional and phylogenetic diversity of forest mammals | 10.1007/s10531-023-02715-7 | No | Conservation biology | China | Vertebrates | Topographic, Climatic, Land cover | Other | Regression | Yes | Biodiversity and Conservation | 3 | 1 | 2 | 6 | High |
| 2023 | Panda B.P. | Influence of crop heterogeneity on avian diversity and trait composition in urban agro-ecosystems | 10.1007/s12595-023-00497-y | No | Conservation biology | India | Vertebrates | Vegetation | Other | Regression | Yes | Proceedings of the Zoological Society | 1 | 1 | 2 | 4 | Low |
| 2023 | Graham | Teasing apart fine- and coarse-scale effects of environmental heterogeneity on tree species richness in Europe | 10.1016/j.baae.2022.12.002 | No | Biogeography | Europe | Plants | Topographic | Other | Regression | Yes | Basic and Applied Ecology | 1 | 1 | 2 | 4 | Low |
| 2023 | Heidrich | Effects of heterogeneity on the ecological diversity and redundancy of forest fauna | 10.1016/j.baae.2023.10.005 | No | Ecology | Germany | Plants, Invertebrates, Vertebrates, Microorganisms | Topographic, Vegetation | Other | Regression | Yes | Basic and Applied Ecology | 2 | 1 | 2 | 5 | Medium |
| 2023 | Jia | Environmental heterogeneity contributes to population genetic diversity and spatial genetic structure of coral-algal symbiosis of platygyra daedalea in the northern South China Sea | 10.1016/j.ecolind.2023.110599 | No | Genetics / Population genetics | China | Microorganisms | Aquatic | Other | Ordination/RDA | Yes | Ecological Indicators | 1 | 1 | 3 | 5 | Medium |
| 2023 | Qiao (b) | Biodiversity contributes to stabilizing ecosystem productivity across spatial scales as much as environmental heterogeneity in a large temperate forest region | 10.1016/j.foreco.2022.120695 | No | Ecology | China | Plants | Soil, Climatic, Topographic, Vegetation, Land cover | Other | Regression | Yes | Forest Ecology and Management | 3 | 1 | 2 | 6 | High |
| 2023 | Sun (a) | Surviving hot summer: Roles of phenotypic plasticity of intertidal mobile species considering microhabitat environmental heterogeneity | 10.1016/j.jtherbio.2023.103686 | No | Physiology / Ecophysiology | China | Invertebrates | Aquatic, Climatic | Mean | Other | Yes | Journal of Thermal Biology | 2 | 1 | 1 | 4 | Low |
| 2023 | Basuri | Influence of environmental heterogeneity on the distribution of micro-zooplankton community along the tropical coastal waters | 10.1016/j.marpolbul.2023.115546 | No | Ecology | India | Microorganisms | Aquatic | Other | Ordination/RDA | Yes | Marine Pollution Bulletin | 1 | 1 | 3 | 5 | Medium |
| 2023 | Huang Y. | Community assemblages and species coexistence of prokaryotes controlled by local environmental heterogeneity in a cold seep water column | 10.1016/j.scitotenv.2023.161725 | Yes | Ecology | China | Microorganisms | Aquatic | Other | Ordination/RDA | Yes | Science of the Total Environment | 1 | 1 | 3 | 5 | Medium |
| 2023 | Daleo | Environmental heterogeneity modulates the effect of plant diversity on the spatial variability of grassland biomass | 10.1038/s41467-023-37395-y | No | Ecology | Global | Plants | Soil, Climatic | Other | Regression | Yes | Nature Communications | 2 | 1 | 2 | 5 | Medium |
| 2023 | Montoya-Snchez | Landscape heterogeneity and soil biota are central to multi-taxa diversity for oil palm landscape restoration | 10.1038/s43247-023-00875-6 | No | Conservation biology | Indonesia | Plants, Invertebrates, Microorganisms | Vegetation, Soil, Land cover | Other | Ordination/RDA | Yes | Communications Earth & Environment | 3 | 1 | 3 | 7 | High |
| 2023 | Ramos | Alpha and beta diversity of planktonic microcrustaceans are associated with environmental heterogeneity in the Frades River basin, Brazil | 10.1080/01650521.2021.1933702 | No | Ecology | Brazil | Invertebrates | Aquatic | Other | Ordination/RDA | Yes | Studies on Neotropical Fauna and Environment | 1 | 1 | 3 | 5 | Medium |
| 2023 | Miller Z.R. | Habitat heterogeneity, environmental feedbacks, and species coexistence across timescales | 10.1086/724821 | Yes | Ecology | United States | Other | Other | Other | Other | Yes | American Naturalist | 1 | 1 | 1 | 3 | Low |
| 2023 | Glasnovic | Areography, environmental heterogeneity and spatial models explain patterns of past and present diversity in Edraianthus (campanulaceae) | 10.1093/botlinnean/boac079 | No | Biogeography | Europe | Plants | Topographic, Climatic | Other | Other | Yes | Botanical Journal of the Linnean Society | 2 | 1 | 1 | 4 | Low |
| 2023 | Karuno | Impacts of climate change on herpetofauna diversity in the Qinghai-Tibetan plateau | 10.1111/cobi.14155 | No | Conservation biology | China | Vertebrates | Topographic, Climatic | Other | Other | Yes | Conservation Biology | 2 | 1 | 1 | 4 | Low |
| 2023 | He | Patterns in aquatic metacommunities are associated with environmental and trait heterogeneity | 10.1111/fwb.14011 | No | Ecology | China | Invertebrates, Plants | Aquatic | Other | Ordination/RDA | Yes | Freshwater Biology | 1 | 1 | 3 | 5 | Medium |
| 2023 | Qiao (c) | Latitudinal patterns of forest ecosystem stability across spatial scales as affected by biodiversity and environmental heterogeneity | 10.1111/gcb.16593 | No | Ecology | China | Plants | Topographic, Soil, Climatic, Vegetation, Land cover | Other | Regression | Yes | Global Change Biology | 3 | 1 | 2 | 6 | High |
| 2023 | Malanson | Area, environmental heterogeneity, scale and the conservation of alpine diversity | 10.1111/jbi.14573 | No | Biogeography | Europe | Plants | Topographic, Soil, Climatic | Index | Regression | Yes | Journal of Biogeography | 3 | 1 | 2 | 6 | High |
| 2023 | Yan | Habitat heterogeneity determines species richness on small habitat islands in a fragmented landscape | 10.1111/jbi.14594 | No | Conservation biology | China | Plants | Vegetation, Land cover | Other | Regression | Yes | Journal of Biogeography | 2 | 1 | 2 | 5 | Medium |
| 2023 | Maskell | Inconsistent relationships between area, heterogeneity and plant species richness in temperate farmed landscapes | 10.1111/oik.09720 | No | Conservation biology | United Kingdom | Plants | Land cover, Topographic, Soil | Index | Regression | Yes | Oikos | 3 | 1 | 2 | 6 | High |
| 2023 | Xue | Soil nutrient heterogeneity alters productivity and diversity of experimental plant communities under multiple global change factors | 10.1111/oik.10189 | No | Ecology | China | Plants | Soil, Climatic | Other | ANOVA | Yes | Oikos | 2 | 1 | 1 | 4 | Low |
| 2023 | Li | Environmental heterogeneity drives population genetic divergence of a key agricultural pest, Empoasca onukii | 10.1127/entomologia/2023/1695 | No | Genetics / Population genetics | China | Invertebrates | Climatic | Other | Other | Yes | Entomologia Generalis | 1 | 1 | 1 | 3 | Low |
| 2023 | Sun (b) | Phytoplankton and microzooplankton community structure and assembly mechanisms in northwestern pacific ocean estuaries with environmental heterogeneity and geographic segregation | 10.1128/spectrum.04926-22 | No | Ecology | China | Microorganisms | Aquatic | Other | Ordination/RDA | Yes | Microbiology Spectrum | 1 | 1 | 3 | 5 | Medium |
| 2023 | Furness | The jigsaw model: A biogeographic model that partitions habitat heterogeneity from area | 10.21425/f5fbg58477 | No | Biogeography | United Kingdom | Other | Other | Other | Other | Yes | Frontiers Of Biogeography | 1 | 1 | 1 | 3 | Low |
| 2023 | Catella | Effects of abiotic heterogeneity on species densities and interaction strengths lead to different spatial biodiversity patterns | 10.3389/fevo.2023.1071375 | No | Ecology | United States | Other | Other | Other | Other | Yes | Frontiers in Ecology and Evolution | 1 | 1 | 1 | 3 | Low |
| 2023 | Dalkiran | Environmental heterogeneity and salinity gradient impacted the alpha and beta diversities of diatom assemblages in a coastal delta wetland | 10.3390/w15193414 | No | Ecology | Turkey | Microorganisms | Aquatic | Other | Ordination/RDA | Yes | Water (Switzerland) | 1 | 1 | 3 | 5 | Medium |
| 2023 | Jiang | Environmental heterogeneity determines beta diversity and species turnover for woody plants along an elevation gradient in subtropical forests of China | 10.48130/fr-2023-0026 | No | Ecology | China | Plants | Topographic, Climatic | Other | Ordination/RDA | Yes | Forestry Research | 2 | 1 | 3 | 6 | High |
| 2023 | Pereira | Influence of environmental heterogeneity on mirmecofauna in different land uses in the municipality of Bom Despacho, Minas Gerais State | 10.5902/1980509864534 | No | Ecology | Brazil | Invertebrates | Vegetation, Land cover | Index | Other | Yes | Ciencia Florestal | 2 | 1 | 1 | 4 | Low |
| 2024 | Ek-Rodrguez | Environmental heterogeneity influences liana community differentiation across a neotropical rainforest landscape | 10.1002/ece3.11170 | Yes | Ecology | Mexico | Plants | Soil, Topographic | Other | Ordination/RDA | Yes | Ecology and Evolution | 2 | 1 | 3 | 6 | High |
| 2024 | Zibold | Fungi and deadwood diversity: A test of the area-heterogeneity trade-off hypothesis | 10.1111/1365-2745.14352 | Yes | Ecology | Germany | Microorganisms | Vegetation | Index | Regression | Yes | Journal of Ecology | 1 | 1 | 2 | 4 | Low |
| 2024 | Chen | (epi)genomic adaptation driven by fine geographical scale environmental heterogeneity after recent biological invasions | 10.1002/eap.2772 | No | Genetics / Population genetics | Canada | Invertebrates | Aquatic, Climatic | Other | Other | Yes | Ecological Applications | 2 | 1 | 1 | 4 | Low |
| 2024 | Wojciechowski | Heterogeneity promotes resilience in restored prairie: Implications for the environmental heterogeneity hypothesis | 10.1002/eap.3006 | No | Conservation biology | United States | Plants | Soil | Other | ANOVA | Yes | Ecological Applications | 1 | 1 | 1 | 3 | Low |
| 2024 | Ospina-Bautista | Environmental heterogeneity at two spatial scales affects litter diversity-decomposition relationships | 10.1002/ecy.4280 | No | Ecology |  | Plants | Topographic, Climatic | Other | ANOVA | Yes | Ecology | 2 | 1 | 1 | 4 | Low |
| 2024 | Rosas | Spatial heterogeneity of zooplankton community in an eutrophicated tropical estuary | 10.1007/s00027-024-01108-4 | No | Ecology | Brazil | Invertebrates | Aquatic | Other | Ordination/RDA | Yes | Aquatic Sciences | 1 | 1 | 3 | 5 | Medium |
| 2024 | Faria | Land use types determine environmental heterogeneity and aquatic insect diversity in Amazonian streams | 10.1007/s10750-023-05190-x | No | Conservation biology | Brazil | Invertebrates | Aquatic, Land cover | Other | Ordination/RDA | Yes | Hydrobiologia | 2 | 1 | 3 | 6 | High |
| 2024 | Cantanhde | Effects of deforestation on environmental heterogeneity and its role in the distribution of fish species and functional groups in Amazonian streams | 10.1007/s10750-023-05201-x | No | Conservation biology | Brazil | Vertebrates | Aquatic, Vegetation, Land cover | Other | Regression | Yes | Hydrobiologia | 3 | 1 | 2 | 6 | High |
| 2024 | da Silva | Influence of environmental heterogeneity on the structure of the dung beetle (coleoptera: Scarabaeidae: Scarabaeinae) assemblage of the Cerrado savanna of northeastern Brazil | 10.1007/s10841-024-00566-3 | No | Ecology | Brazil | Invertebrates | Vegetation, Land cover | Other | Other | Yes | Journal of Insect Conservation | 2 | 1 | 1 | 4 | Low |
| 2024 | Veras | Heterogeneity in altered streams does not increase the richness of stream specialist species of Odonata in the maranhense Cerrado | 10.1007/s10841-024-00582-3 | No | Conservation biology | Brazil | Invertebrates | Aquatic, Vegetation | Other | Regression | No | Journal of Insect Conservation | 2 | 1 | 2 | 5 | Medium |
| 2024 | Havrilla | Soil cover heterogeneity associated with biocrusts predicts patch-level plant diversity patterns | 10.1007/s10980-024-01986-x | No | Ecology | United States | Plants | Soil | Other | Regression | Yes | Landscape Ecology | 1 | 1 | 2 | 4 | Low |
| 2024 | Demartn | High amphibian diversity throughout urban environmental heterogeneity | 10.1007/s11252-024-01574-6 | No | Conservation biology | Argentina | Vertebrates | Vegetation, Land cover, Aquatic | Other | Ordination/RDA | Yes | Urban Ecosystems | 3 | 1 | 3 | 7 | High |
| 2024 | Travassos | Ant (hymenoptera: Formicidae) assemblages in seashore vegetation (restinga) formations along the northern coast of the state of Bahia, Brazil | 10.1007/s11852-024-01069-8 | No | Conservation biology | Brazil | Invertebrates | Vegetation, Land cover | Other | Other | Yes | Journal Of Coastal Conservation | 2 | 1 | 1 | 4 | Low |
| 2024 | Cao | Landscape connection and patch complexity explain plant community similarity in sandy grasslands better than habitat amount and landscape heterogeneity via network analysis | 10.1007/s42974-024-00185-7 | No | Ecology | China | Plants | Land cover, Topographic | Other | Regression | No | Community Ecology | 2 | 1 | 2 | 5 | Medium |
| 2024 | Le Goff M. | Environmental, social and morphological drivers of fissionâ€“fusion dynamics in a social ungulate | 10.1016/j.anbehav.2023.10.014 | No | Ecology | Canada | Vertebrates | Vegetation | Other | Other | Yes | Animal Behaviour | 1 | 1 | 1 | 3 | Low |
| 2024 | Yan | Leaf trait network variations with woody species diversity and habitat heterogeneity in degraded karst forests | 10.1016/j.ecolind.2024.111896 | No | Ecology | China | Plants | Soil, Topographic, Climatic | Other | Ordination/RDA | Yes | Ecological Indicators | 3 | 1 | 3 | 7 | High |
| 2024 | Li (a) | Environmental DNA metabarcoding reveals the influence of environmental heterogeneity on microeukaryotic plankton in the offshore waters of east China sea | 10.1016/j.envres.2024.119921 | No | Ecology | China | Microorganisms | Aquatic | Other | Ordination/RDA | Yes | Environmental Research | 1 | 1 | 3 | 5 | Medium |
| 2024 | Shen | Seasonal dynamics of environmental heterogeneity augment microbial interactions by regulating community structure in different trophic lakes | 10.1016/j.envres.2024.120031 | No | Ecology | China | Microorganisms | Aquatic, Climatic | Other | Other | Yes | Environmental Research | 2 | 1 | 1 | 4 | Low |
| 2024 | Zheng | Soil nutrient heterogeneity affects community stability through changing asynchrony in an alpine meadow on the Qinghai-Tibetan Plateau | 10.1016/j.gecco.2024.e03045 | No | Ecology | China | Plants | Soil | Other | ANOVA | Yes | Global Ecology and Conservation | 1 | 1 | 1 | 3 | Low |
| 2024 | Guo | Soil resource heterogeneity promotes species richness only at a fine scale at the early restoration of karst abandoned farmland | 10.1016/j.isci.2024.111408 | No | Ecology | China | Plants | Soil | Other | Regression | Yes | Iscience | 1 | 1 | 2 | 4 | Low |
| 2024 | Hu | Environmental heterogeneity of cold seep by biological trait analysis of marine nematodes at Site F cold seep in South China sea | 10.1016/j.marpolbul.2023.115932 | No | Ecology | China | Invertebrates | Aquatic, Soil | Other | Ordination/RDA | Yes | Marine Pollution Bulletin | 2 | 1 | 3 | 6 | High |
| 2024 | Guerrina M. | Unveiling the hypotheses of endemic richness: A study case in the Southwestern Alps | 10.1016/j.ppees.2024.125792 | No | Conservation biology | Italy | Plants | Topographic, Climatic | Other | Regression | Yes | Perspectives in Plant Ecology, Evolution and Systematics | 2 | 1 | 2 | 5 | Medium |
| 2024 | Bakker | Remotely sensed habitat diversity predicts species diversity on coral reefs | 10.1016/j.rse.2024.113990 | No | Conservation biology | Global | Vertebrates, Invertebrates | Aquatic | Index | Regression | Yes | Remote Sensing Of Environment | 1 | 1 | 2 | 4 | Low |
| 2024 | Li (b) | Environmental heterogeneity caused by large-scale cultivation of Pyropia haitanensis shapes multi-group biodiversity distribution in coastal areas | 10.1016/j.scitotenv.2024.172692 | No | Ecology | China | Microorganisms | Aquatic | Other | Ordination/RDA | Yes | Science of the Total Environment | 1 | 1 | 3 | 5 | Medium |
| 2024 | Kazmi | Vertical variations and environmental heterogeneity drove the symphony of periphytic protozoan fauna in marine ecosystems | 10.1016/j.scitotenv.2024.173115 | No | Ecology | China | Microorganisms | Aquatic | Other | Ordination/RDA | Yes | Science of the Total Environment | 1 | 1 | 3 | 5 | Medium |
| 2024 | Dek | Meso-scale environmental heterogeneity drives plant trait distributions in fragmented dry grasslands | 10.1016/j.scitotenv.2024.174355 | No | Conservation biology | Hungary, Bulgaria | Plants | Topographic, Soil | Other | Other | Yes | Science of the Total Environment | 2 | 1 | 1 | 4 | Low |
| 2024 | Chan | Microhabitat coverage influences avian species composition more than habitat heterogeneity in Hong Kong urban parks | 10.1016/j.ufug.2024.128519 | No | Conservation biology | Hong Kong | Vertebrates | Vegetation, Land cover | Index | Regression | No | Urban Forestry and Urban Greening | 2 | 1 | 2 | 5 | Medium |
| 2024 | Karimi | Chromosomal evolution, environmental heterogeneity, and migration drive spatial patterns of species richness in Calochortus (Liliaceae) | 10.1073/pnas.2305228121 | No | Evolutionary biology | United States, Mexico | Plants | Topographic, Soil, Climatic | Other | Regression | Yes | Proceedings of the National Academy of Sciences of the United States of America | 3 | 1 | 2 | 6 | High |
| 2024 | Padmanabha | Landscape and environmental heterogeneity support coexistence in competitive metacommunities | 10.1073/pnas.2410932121 | No | Ecology | Italy | Other | Other | Other | Other | Yes | Proceedings of the National Academy of Sciences of the United States of America | 1 | 1 | 1 | 3 | Low |
| 2024 | Reis-Venncio | From light to darkness: The duality of influence of habitat heterogeneity on Neotropical terrestrial cave invertebrate communities | 10.1080/01650521.2022.2095832 | No | Ecology | Brazil | Invertebrates | Other | Other | Regression | Yes | Studies on Neotropical Fauna and Environment | 1 | 1 | 2 | 4 | Low |
| 2024 | Conceio | Guiding seed movement: Environmental heterogeneity drives genetic differentiation in Plathymenia reticulata, providing insights for restoration | 10.1093/aobpla/plae032 | No | Genetics / Population genetics | Brazil | Plants | Soil, Climatic | Other | Other | Yes | AoB PLANTS | 2 | 1 | 1 | 4 | Low |
| 2024 | Mausbach | Large-scale heterogeneity gradient drives the structure and biodiversity of branchiopod (anostraca, cladocera) egg-banks in the Nebraska Sandhills wetlands, USA | 10.1093/jcbiol/ruae045 | No | Ecology | United States | Invertebrates | Aquatic, Soil | Other | Other | Yes | Journal of Crustacean Biology | 2 | 1 | 1 | 4 | Low |
| 2024 | Cui | Contrasting mechanisms of non-vascular and vascular plants on spatial turnover in multifunctionality in the Antarctic continent | 10.1111/1365-2745.14346 | No | Ecology | Antarctica | Plants, Microorganisms | Soil, Climatic | Other | Ordination/RDA | Yes | Journal of Ecology | 2 | 1 | 3 | 6 | High |
| 2024 | Liu (a) | Climate heterogeneity, season variation, and sexual dimorphism modulate the association between MHC II diversity and parasite variation in striped hamster | 10.1111/1749-4877.12791 | No | Ecology | China | Vertebrates | Climatic, Other | Other | Other | Yes | Integrative Zoology | 2 | 1 | 1 | 4 | Low |
| 2024 | Moctezuma | Multifactorial structuring in a local guild of coprophilic scarab beetles from the Mexican transition zone: Analyses of ecological hypotheses | 10.1111/een.13332 | No | Ecology | Mexico | Invertebrates | Vegetation, Climatic | Other | Ordination/RDA | Yes | Ecological Entomology | 2 | 1 | 3 | 6 | High |
| 2024 | Santos | Zooplankton community heterogeneity and trophic interactions in response to environmental drivers in a mesotidal estuary on the Brazilian equatorial margin-lenÃ§Ã³is maranhenses national park | 10.1111/fwb.14303 | No | Ecology | Brazil | Invertebrates | Aquatic | Other | Ordination/RDA | Yes | Freshwater Biology | 1 | 1 | 3 | 5 | Medium |
| 2024 | Zhang (a) | Power law in species-area relationship overestimates bacterial diversity in grassland soils at larger scales | 10.1111/geb.13825 | No | Ecology | China | Microorganisms | Soil | Other | Regression | Yes | Global Ecology and Biogeography | 1 | 1 | 2 | 4 | Low |
| 2024 | Li (c) | Tree diversity across multiple scales and environmental heterogeneity promote ecosystem multifunctionality in a large temperate forest region | 10.1111/geb.13880 | No | Ecology | China | Plants | Topographic, Soil, Climatic, Vegetation | Other | Regression | Yes | Global Ecology and Biogeography | 3 | 1 | 2 | 6 | High |
| 2024 | Vernham | What is the relationship between plant trait diversity and geodiversity? a plot-based, pan-European analysis | 10.1111/geb.13904 | No | Ecology | Europe | Plants | Topographic, Soil | Other | Regression | Yes | Global Ecology and Biogeography | 2 | 1 | 2 | 5 | Medium |
| 2024 | Wang | Diversification in birds is promoted by plant diversity, topographic heterogeneity and stable paleoclimate | 10.1111/jbi.14974 | No | Biogeography | China | Vertebrates | Topographic, Vegetation, Climatic | Other | Regression | Yes | Journal of Biogeography | 3 | 1 | 2 | 6 | High |
| 2024 | Trubina | Role of environmental heterogeneity in the species distribution of vascular plants in periods of high and low emissions from a Copper Smelter | 10.1134/s1995425524010141 | No | Conservation biology | Russia | Plants | Soil, Climatic | Other | Other | Yes | Contemporary Problems Of Ecology | 2 | 1 | 1 | 4 | Low |
| 2024 | Prez-Pedraza | The effects of environmental heterogeneity on the morphological similarity of toothed-leafed red oak species (Acutifoliae ; Quercus) in Mexico and central america | 10.17129/botsci.3526 | No | Evolutionary biology | Mexico, Guatemala | Plants | Topographic, Climatic | Mean | Regression | Yes | Botanical Sciences | 2 | 1 | 2 | 5 | Medium |
| 2024 | Liu (b) | Spatial heterogeneity affects the spatial distribution patterns of caragana tibetica scrubs | 10.3390/f15122072 | No | Ecology | China | Plants | Soil, Topographic | Other | Other | Yes | Forests | 2 | 1 | 1 | 4 | Low |
| 2024 | de Almeida | Environmental heterogeneity and its influence on the taxonomic richness and diversity of the Tropical Dry Forest in Pernambuco, Brazil | 10.5902/1980509873821 | No | Ecology | Brazil | Plants | Topographic, Climatic | Other | Ordination/RDA | Yes | Ciencia Florestal | 2 | 1 | 3 | 6 | High |
| 2024 | Vilanova | Interrelations between environmental heterogeneity, competition, and phylogeny in the structure of a lizard assemblage in a hypoxerophilic Caatinga area of Brazil | NA | No | Ecology | Brazil | Vertebrates | Vegetation | Other | Other | Yes | Herpetological Conservation and Biology | 1 | 1 | 1 | 3 | Low |
| 2025 | Woods | Microtopographic heterogeneity affects habitat specialization and diversity of understory plants in a northern temperate rainforest | 10.1007/s11258-024-01469-8 | Yes | Ecology | United States | Plants | Topographic, Vegetation | Index | Regression | Yes | Plant Ecology | 2 | 1 | 2 | 5 | Medium |
| 2025 | Wan | Environmental heterogeneity drives the dark diversity of global plant communities | 10.1111/jbi.70043 | Yes | Biogeography | Global | Plants | Topographic, Climatic, Soil, Vegetation | Other | Regression | Yes | Journal of Biogeography | 3 | 1 | 2 | 6 | High |
| 2025 | Antonio | Environmental heterogeneity as a determinant of bee diversity patterns in the Atlantic Forest | 10.21425/fob.18.142410 | Yes | Biogeography | Brazil | Invertebrates | Topographic, Climatic | Other | Regression | Yes | Frontiers Of Biogeography | 2 | 1 | 2 | 5 | Medium |
| 2025 | Arroyo | Intraspecific trait variation in tree species responds to environmental heterogeneity at range-wide but not local scales | 10.1002/ece3.72224 | No | Ecology | Puerto Rico | Plants | Topographic, Climatic | Other | Regression | Yes | Ecology and Evolution | 2 | 1 | 2 | 5 | Medium |
| 2025 | Gasperini | Environmental heterogeneity as a differential driver for density of two sympatric rodent species | 10.1002/ece3.72268 | No | Ecology | Italy | Vertebrates | Vegetation | Other | Regression | Yes | Ecology and Evolution | 1 | 1 | 2 | 4 | Low |
| 2025 | Tang | Environmental heterogeneity among microhabitats shapes the diversity and community composition of soil eukaryotes in the Gurbantunggut Desert | 10.1002/ldr.70068 | No | Ecology | China | Microorganisms | Soil | Other | Ordination/RDA | Yes | Land Degradation and Development | 1 | 1 | 3 | 5 | Medium |
| 2025 | Katz L. | Using Bayesian network Inference and underwater imagery to understand the influence of environmental heterogeneities on benthic community structure in the Antarctic Peninsula | 10.1007/s00300-025-03407-4 | No | Ecology | Antarctica | Invertebrates | Aquatic, Topographic | Other | Other | Yes | Polar Biology | 2 | 1 | 1 | 4 | Low |
| 2025 | Saboy-Acosta | Heterogeneity does matter: Estimating relationships between anuran diversity and the environment at different spatial scales in a protected pÃ¡ramo of Colombia | 10.1007/s10980-024-02015-7 | No | Conservation biology | Colombia | Vertebrates | Vegetation, Aquatic, Land cover | Other | Regression | Yes | Landscape Ecology | 3 | 1 | 2 | 6 | High |
| 2025 | Jiang | Environmental heterogeneity governing river macrophyte beta diversity in Europe is scale- and context-dependent | 10.1007/s10980-025-02212-y | No | Ecology | Portugal, Poland, Germany, Denmark, Finland | Plants | Aquatic, Topographic, Climatic | Other | Regression | Yes | Landscape Ecology | 3 | 1 | 2 | 6 | High |
| 2025 | Finand | Perceived urban microhabitat heterogeneity impacts carabid beetle communities | 10.1007/s10980-025-02260-4 | No | Conservation biology | Finland | Invertebrates | Vegetation, Land cover | Other | Regression | No | Landscape Ecology | 2 | 1 | 2 | 5 | Medium |
| 2025 | Akachukwu | Effects of environmental heterogeneity, spatial extent, and seasonality on phytoplankton beta diversity in Niger State reservoirs in north central Nigeria | 10.1007/s11356-025-36814-5 | No | Ecology | Nigeria | Microorganisms | Aquatic | Other | Ordination/RDA | Yes | Environmental Science and Pollution Research | 1 | 1 | 3 | 5 | Medium |
| 2025 | Ponzio | Beyond classical invasions hypotheses: Investigating the impact of agricultural landscape simplification on wild pig populations | 10.1016/j.biocon.2025.111321 | No | Conservation biology | Brazil | Vertebrates | Land cover, Vegetation | Other | Regression | Yes | Biological Conservation | 2 | 1 | 2 | 5 | Medium |
| 2025 | Barrett | Fine-scale surface complexity promotes temperature extremes but reduces the spatial extent of refugia on coastal rocks | 10.1016/j.ecolmodel.2024.110969 | No | Physiology / Ecophysiology | Israel | Invertebrates | Topographic | Other | Other | Yes | Ecological Modelling | 1 | 1 | 1 | 3 | Low |
| 2025 | Liu | Energy drives multi-faceted diversity patterns of benthic macroinvertebrates in China with important conservation implications | 10.1016/j.gecco.2025.e03951 | No | Biogeography | China | Invertebrates | Aquatic, Climatic, Topographic, Soil | Other | Regression | No | Global Ecology and Conservation | 3 | 1 | 2 | 6 | High |
| 2025 | Hu | Aridity-driven soil community heterogeneity enhances biodiversity-multifunctionality relationships in dryland ecosystems | 10.1016/j.geoderma.2025.117635 | No | Ecology | China | Microorganisms | Soil, Climatic | Other | Regression | Yes | Geoderma | 2 | 1 | 2 | 5 | Medium |
| 2025 | Zhang | Environmental heterogeneity and geographic isolation drive prokaryotic community succession in acid mine drainage from abandoned coal mines | 10.1016/j.jenvman.2025.127780 | No | Ecology | China | Microorganisms | Aquatic, Soil | Other | Ordination/RDA | Yes | Journal of Environmental Management | 2 | 1 | 3 | 6 | High |
| 2025 | Veldkornet | Environmental heterogeneity as a driver of species and phylogenetic diversity in coastal salt marshes | 10.1016/j.sajb.2025.01.024 | No | Conservation biology | South Africa | Plants | Aquatic, Topographic, Soil | Other | Regression | Yes | South African Journal of Botany | 3 | 1 | 2 | 6 | High |
| 2025 | Lee | Environmental heterogeneity and its influence on fern diversity in a low-altitude mountain forest in central Taiwan | 10.1038/s41598-025-28048-9 | No | Ecology | Taiwan | Plants | Topographic, Soil, Vegetation | Other | Ordination/RDA | Yes | Scientific Reports | 3 | 1 | 3 | 7 | High |
| 2025 | Mndez-Rojas | Local and landscape heterogeneity promotes high functional structure of rove beetle community in Andean coffee crops | 10.1111/1365-2664.70078 | No | Ecology | Colombia | Invertebrates | Vegetation, Land cover | Other | Ordination/RDA | Yes | Journal of Applied Ecology | 2 | 1 | 3 | 6 | High |
| 2025 | Musthafa | Climate heterogeneity shapes the diversity of specialist beetle species across mountains in Malaysia | 10.1111/btp.70001 | No | Biogeography | Malaysia | Invertebrates | Topographic, Climatic | Other | Other | Yes | Biotropica | 2 | 1 | 1 | 4 | Low |
| 2025 | Wurz | Effects of species richness and turnover on ecosystem functioning in heterogeneous environments of two tropical mountains | 10.1111/ddi.70093 | No | Ecology | Ecuador, Tanzania | Plants, Vertebrates, Invertebrates | Topographic, Climatic | Other | Other | Yes | Diversity and Distributions | 2 | 1 | 1 | 4 | Low |
| 2025 | Surez | Environmental heterogeneity, rather than stability, explains spider assemblage differences between ecosystems | 10.1111/ecog.07454 | No | Ecology | Spain | Invertebrates | Vegetation, Climatic | Other | Other | Yes | Ecography | 2 | 1 | 1 | 4 | Low |
| 2025 | Tsamba | Fine scale patterns and drivers of plant species richness on a sub-Antarctic Island | 10.1111/jvs.70015 | No | Conservation biology | Antarctica | Plants | Topographic, Climatic | Other | Regression | Yes | Journal of Vegetation Science | 2 | 1 | 2 | 5 | Medium |
| 2025 | Magurran | Spatial and temporal heterogeneity foster high fish biodiversity in an Amazonian floodplain | 10.1126/sciadv.adv9672 | No | Ecology | Brazil | Vertebrates | Aquatic | Other | Other | Yes | Science Advances | 1 | 1 | 1 | 3 | Low |
| 2025 | Xiang H. | Spatial heterogeneity of microbial community structure and its environmental drivers in surface sediments of Erhai lake | 10.1371/journal.pone.0322915 | No | Ecology | China | Microorganisms | Aquatic, Soil | Other | Ordination/RDA | Yes | PLoS ONE | 2 | 1 | 3 | 6 | High |
| 2025 | Pozas-Franco | Environmental heterogeneity plays a bigger role than diet quality in driving divergent California sea lion population trends | 10.1371/journal.pone.0324108 | No | Conservation biology | United States | Vertebrates | Aquatic, Climatic | Other | Other | No | PLoS ONE | 2 | 1 | 1 | 4 | Low |
| 2025 | Liborio C.H.L. | Empirical evaluations between beta-diversity, environmental heterogeneity, and spatial extent among multiple taxonomic groups; [testes empÃ­ricos das relaÃ§Ãµes entre diversidade beta, heterogeneidade ambiental e extensÃ£o espacial entre mÃºltiplos grupos taxonÃ´micos] | 10.1590/s2179-975x10124 | No | Ecology | United States | Microorganisms, Invertebrates | Aquatic | Other | Regression | Yes | Acta Limnologica Brasiliensia | 1 | 1 | 2 | 4 | Low |
| 2025 | Sousa F.D.R. | Environmental heterogeneity in wetlands increases alpha and beta diversity of cladocerans (crustacea, branchiopoda) at local and regional scale; [a heterogeneidade ambiental em Ã¡reas Ãºmidas aumenta a diversidade alfa e beta de cladÃ³ceros (crustacea, branchiopoda) em escala local e regional] | 10.1590/s2179-975x2624 | Yes | Ecology | Brazil | Invertebrates | Aquatic, Vegetation | Other | Regression | Yes | Acta Limnologica Brasiliensia | 2 | 1 | 2 | 5 | Medium |
| 2025 | Wu Q. | Heterogeneity of the horizontal environment drives community assemblages and species coexistence of prokaryotic communities in cold seep sediments | 10.3389/fmicb.2025.1687453 | No | Ecology | China | Microorganisms | Aquatic, Soil | Other | Ordination/RDA | Yes | Frontiers in Microbiology | 2 | 1 | 3 | 6 | High |
| 2025 | Wang (a) | Geographic isolation and environmental heterogeneity shape population genetic differentiation of a medicinal plant, amla (Phyllanthus emblica L.) in river valleys of Yunnan, China | 10.3389/fpls.2025.1648822 | No | Genetics / Population genetics | China | Plants | Topographic, Climatic | Other | Other | Yes | Frontiers in Plant Science | 2 | 1 | 1 | 4 | Low |
| 2025 | Wei | Spatial distribution and intraspecific and interspecific associations of dominant tree species in a deciduous broad-leaved forest in Shennongjia, China | 10.3390/d17050335 | No | Ecology | China | Plants | Topographic, Soil | Other | Other | Yes | Diversity | 2 | 1 | 1 | 4 | Low |
| 2025 | He | Environmental heterogeneity drives diversity across forest strata in Hopea hainanensis communities | 10.3390/d17080556 | No | Ecology | China | Plants | Soil, Topographic | Other | ANOVA | Yes | Diversity | 2 | 1 | 1 | 4 | Low |
| 2025 | Kadawatha | Edge feathering across forest-meadow ecotones increases light heterogeneity and understory plant diversity | 10.3390/f16030441 | No | Conservation biology | United States | Plants | Vegetation | Index | Regression | Yes | Forests | 1 | 1 | 2 | 4 | Low |
| 2025 | Santos-Silva | Fine-scale environmental heterogeneity shapes post-fire macrofungal richness in a Mediterranean relict forest | 10.3390/fire8110438 | No | Conservation biology | Portugal | Microorganisms | Topographic, Vegetation, Climatic | Other | Regression | Yes | Fire | 3 | 1 | 2 | 6 | High |
| 2025 | Zhao | Environmental heterogeneity and altitudinal gradients drive darkling beetle diversity in an alluvial fan | 10.3390/insects16040388 | No | Ecology | China | Invertebrates | Topographic, Soil, Vegetation | Other | Ordination/RDA | Yes | Insects | 3 | 1 | 3 | 7 | High |
| 2025 | Bartha | Fine-scale spatiotemporal variability of plant species composition in old-field succession-the cycling heterogeneity hypothesis revisited | 10.3390/land14122381 | No | Ecology | Hungary | Plants | Other | Other | Other | Yes | Land | 1 | 1 | 1 | 3 | Low |
| 2025 | Liu (b) | Environmental heterogeneity and host genotype jointly shape endophytic bacterial community composition associated with an endemic Chinese Sphagnum species | 10.3390/microorganisms13112538 | No | Ecology | China | Microorganisms | Soil, Climatic | Other | Regression | Yes | Microorganisms | 2 | 1 | 2 | 5 | Medium |
| 2025 | Arajo | Assessing the effect of local heterogeneity on anuran diversity in the Serra da Capivara National Park, Piaui State, Brazil | 10.3897/zookeys.1236.138858 | No | Conservation biology | Brazil | Vertebrates | Vegetation, Aquatic | Other | Regression | Yes | ZooKeys | 2 | 1 | 2 | 5 | Medium |
| 2025 | Pereira | Landscape environmental heterogeneity shaping tree community composition and structure in riparian forests | 10.53661/18069088202549263747 | No | Ecology | Brazil | Plants | Topographic, Aquatic, Soil | Other | Ordination/RDA | Yes | Revista Arvore | 3 | 1 | 3 | 7 | High |

Supporting Information S2. PRISMA flow diagram for the semi-systematic narrative synthesis. Records were retrieved from Web of Science (*n* = 3,188) and Scopus (*n* = 2,689). After deduplication (2,317 duplicates removed), language and date filtering (112 excluded), and two-stage screening (automated + manual review of 750 records), 360 studies met all inclusion criteria and were retained for data extraction. WoS = Web of Science.
